# 9p21 loss confers a cold tumor immune microenvironment and primary resistance to immune checkpoint therapy

**DOI:** 10.1038/s41467-021-25894-9

**Published:** 2021-09-23

**Authors:** Guangchun Han, Guoliang Yang, Dapeng Hao, Yang Lu, Kyaw Thein, Benjamin S. Simpson, Jianfeng Chen, Ryan Sun, Omar Alhalabi, Ruiping Wang, Minghao Dang, Enyu Dai, Shaojun Zhang, Fengqi Nie, Shuangtao Zhao, Charles Guo, Ameer Hamza, Bogdan Czerniak, Chao Cheng, Arlene Siefker-Radtke, Krishna Bhat, Andrew Futreal, Guang Peng, Jennifer Wargo, Weiyi Peng, Humam Kadara, Jaffer Ajani, Charles Swanton, Kevin Litchfield, Jordi Rodon Ahnert, Jianjun Gao, Linghua Wang

**Affiliations:** 1 Department of Genomic Medicine, The University of Texas MD Anderson Cancer Center, Houston, TX USA; 2 Department of Genitourinary Medical Oncology, The University of Texas MD Anderson Cancer Center, Houston, TX USA; 3 Department of Nuclear Medicine, The University of Texas MD Anderson Cancer Center, Houston, TX USA; 4 Department of Investigational Cancer Therapeutics, The University of Texas MD Anderson Cancer Center, Houston, TX USA; 5 Tumour Immunogenomics and Immunosurveillance Laboratory, University College London Cancer Institute, London, UK; 6 Department of Biostatistics, The University of Texas MD Anderson Cancer Center, Houston, TX USA; 7 Department of Pathology, The University of Texas MD Anderson Cancer Center, Houston, TX USA; 8 Department of Medicine, Epidemiology and Population Science, Baylor College of Medicine, Houston, TX USA; 9 Department of Translational Molecular Pathology, The University of Texas MD Anderson Cancer Center, Houston, TX USA; 10 Department of Clinical Cancer Prevention, The University of Texas MD Anderson Cancer Center, Houston, TX USA; 11 Department of Surgical Oncology, The University of Texas MD Anderson Cancer Center, Houston, TX USA; 12 Department of Biology and Biochemistry, University of Houston, Houston, TX USA; 13 Department of Gastrointestinal Medical Oncology, The University of Texas MD Anderson Cancer Center, Houston, TX USA; 14 Cancer Evolution and Genome Instability Laboratory, The Francis Crick Institute, London, UK; 15 Cancer Research UK Lung Cancer Centre of Excellence, University College London Cancer Institute, London, UK; 16 The University of Texas MD Anderson Cancer Center UTHealth Graduate School of Biomedical Sciences (GSBS), Houston, TX USA

**Keywords:** Cancer microenvironment, Cancer immunotherapy

## Abstract

Immune checkpoint therapy (ICT) provides substantial clinical benefits to cancer patients, but a large proportion of cancers do not respond to ICT. To date, the genomic underpinnings of primary resistance to ICT remain elusive. Here, we performed immunogenomic analysis of data from TCGA and clinical trials of anti-PD-1/PD-L1 therapy, with a particular focus on homozygous deletion of 9p21.3 (9p21 loss), one of the most frequent genomic defects occurring in ~13% of all cancers. We demonstrate that 9p21 loss confers “cold” tumor-immune phenotypes, characterized by reduced abundance of tumor-infiltrating leukocytes (TILs), particularly, T/B/NK cells, altered spatial TILs patterns, diminished immune cell trafficking/activation, decreased rate of PD-L1 positivity, along with activation of immunosuppressive signaling. Notably, patients with 9p21 loss exhibited significantly lower response rates to ICT and worse outcomes, which were corroborated in eight ICT trials of >1,000 patients. Further, 9p21 loss synergizes with PD-L1/TMB for patient stratification. A “response score” was derived by incorporating 9p21 loss, PD-L1 expression and TMB levels in pre-treatment tumors, which outperforms PD-L1, TMB, and their combination in identifying patients with high likelihood of achieving sustained response from otherwise non-responders. Moreover, we describe potential druggable targets in 9p21-loss tumors, which could be exploited to design rational therapeutic interventions.

## Abstract (web summary)

The molecular mechanisms of resistance to immune checkpoint therapy remain elusive. Here, the authors perform immunogenomic analysis of TCGA data and data from clinical trials for antiPD-1/PD-L1 therapy and highlight the association of 9p21 loss with a cold tumor microenvironment and resistance to therapy.

## Introduction

Immune checkpoint therapy (ICT) has revolutionized cancer care, leading to remarkable response and improved survival in some patients^1^. Yet, a large proportion of cancers do not respond to the approved immune checkpoint inhibitors (e.g. those targeting PD-1, PD-L1, CTLA-4), especially as monotherapy^2^. It is therefore important to elucidate the mechanistic basis of unresponsiveness to ICT. A key factor leading to primary resistance to ICB is the exclusion or absence/paucity of pre-existing T-cell infiltration in tumors, characteristics of the so-called “non-T-cell-inflamed” or “cold” tumors^3,4^. “Cold” tumor-immune phenotypes can be attributed to many factors including loss of tumor antigen expression, defective recruitment of antigen presenting cells (APCs), absence of antigen presentation, absence of or failed T-cell priming/activation, and impaired T-cell trafficking (i.e. failure to infiltrate the tumor beds)^2,4^. Cellular mechanisms such as activated cancer-associated fibroblasts (CAFs)^5,6^ and other suppressive immune cells^7,8^ that render the tumor microenvironment less permeable to CD8 T cells have also been described. In addition, recent studies have demonstrated that activation of tumor-intrinsic oncogenic pathways including β-catenin^9^, TGF-β^5,6^, and PI3K-AKT-mTOR^7,8^ signaling pathways can promote T-cell exclusion. A higher burden of copy number loss has also been linked to poor response to CTLA-4 and PD-1 blockade in patients with melanoma^10^. However, a universal, tumor-cell intrinsic mechanisms that confer “cold” tumor-immune phenotypes and modulate responses to ICT have not been systematically studied, particularly in the context of large-scale cancer cohorts and clinical trials of ICT.

By increasing the activity of the immune system, ICT can trigger severe immune-related adverse events^11^. Since response rates for ICT are generally low in cancer patients, identifying a non-responder prior to ICT is crucial for: (1) choosing effective therapy for patients with limited treatment and survival time window; (2) sparing patients from unnecessary toxicities; and (3) reducing treatment-related costs. Currently, clinically validated biomarkers that predict response to ICT include high microsatellite instability (MSI-H, occurs in only ~4% of human cancer)^12,13^, tumor-cell PD-L1 expression^14^, and tumor mutational burden (TMB)^15–17^. However, in some large-scale ICT trials, no significant association was observed between levels of tumor-cell PD-L1 expression or TMB and clinical outcomes^6,18^. Stratification by PD-L1 expression or TMB alone is insufficient to identify responders and non-responders in some tumor types^17^. A composite of PD-L1 expression and TMB showed improved but suboptimal performance in identifying patients (e.g., with non-small cell lung cancer, NSCLC) who could achieve durable clinical benefit and was not sufficient to identify patients that are most likely fail to respond to (or derive no benefit from) ICT^17^, highlighting the need for more robust approaches for identifying new biomarkers.

Since homozygous deletion of the chromosomal region 9p21.3 (hereafter referred to as 9p21 loss) represents one of the most frequent somatic copy number alterations (SCNAs) that occur in human cancers^19–21^, attention has been focused on its role in cell cycle regulation due the loss of *CDKN2A/B* in the 9p21 locus. However, the role of 9p21 loss in the modulation of tumor-immune milieu and responses to ICT has not been comprehensively investigated, especially in the context of large cohorts of patients receiving ICT.

Here, we perform integrated immunogenomic analysis of clinical specimens from TCGA study and ICT trials across various cancer types and demonstrate 9p21 loss as a ubiquitous genomic correlate of the “cold” tumor-immune phenotype and primary resistance to ICT. Based upon this finding, we propose a pan-cancer biomarker to predict lack of response to ICT that may guide stratification of cancer patients for appropriate clinical management.

## Results

### 9p21 loss is frequently observed in human cancer and associated with shorter survival

**Fig. 1:**
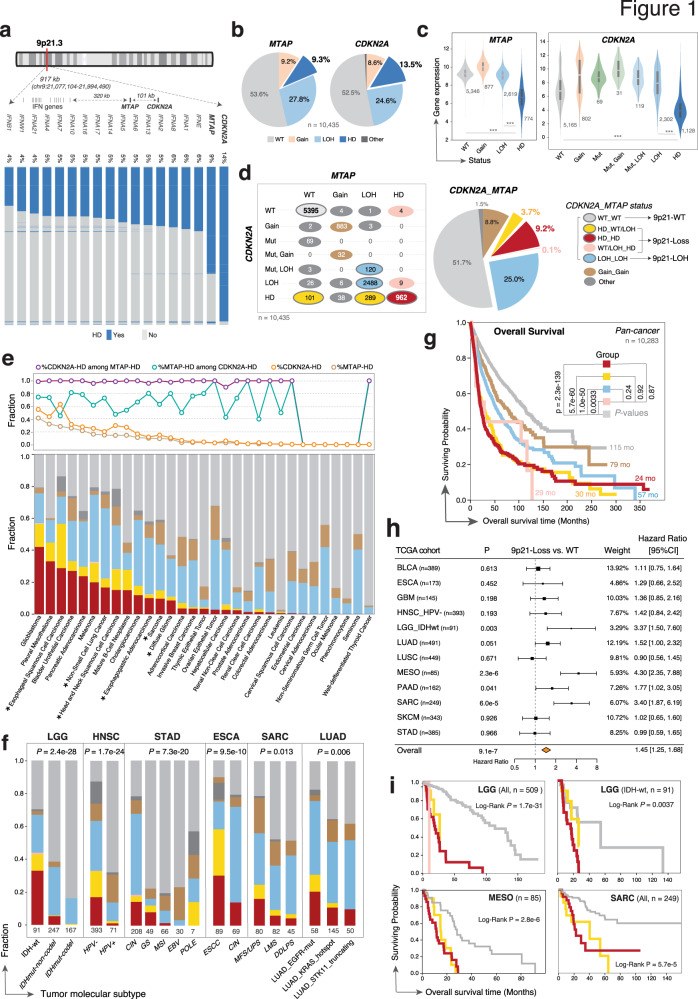
9p21 loss is frequently observed in human cancer and associated with significantly shortened survival. **a** Schematic view of the chromosomal region 9p21.3 showing genes mapped to this focal region, their relative genomic locations, and frequency of 9p21 homozygous deletion (HD) observed in human cancer, based on data from the TCGA studies. **b** Pie charts showing the relative proportions of different types of somatic copy number variations (SCNAs) identified in *MTAP* and *CDKN2A*, respectively. The genomic data of 10,435 tumors from the TCGA program were analyzed. WT wildtype and diploid, LOH loss of heterozygosity (hemizygous deletion), HD homozygous deletion, Gain copy number gain or amplification. **c** The mRNA expression levels of *MTAP* (left) and *CDKN2A* (right) were markedly reduced in tumors with homozygous deletion of the genes. The Numbers of biologically independent samples were labeled on the violinplots. *P* values were calculated by two-sided Wilcoxon rank-sum test and adjusted for multiple testing. Box, median ± interquartile range; whiskers, 1.5× interquartile range. ***P value < 0.001. Exact *P* values were *P* < 2 × 10^−16^ for all comparisons. **d** (left) The relationship of different types of SCNAs between *MTAP* and *CDKN2A* and their relative frequencies (right). Mut mutation. **e** The landscape of 9p21 SCNAs across TCGA cohorts. The colors are the same as shown in the panel **d**. (bottom) Histogram showing the fraction of different types of 9p21 SCNAs (as defined in panel **d**) across TCGA cancer types (see Supplementary Data 1 for a complete list). (top) Line plot showing the fraction of *MTAP* and *CDKN2A* specific events and co-deletions. **f** Representative tumor types demonstrating great variation in the frequencies of 9p21 loss across previously defined molecular subtypes (see Supplementary Data 2 for the abbreviations of disease codes). *P* values were calculated by two-tailed Fisher’s exact tests. **g** The prognostic significance of 9p21 loss at pan-cancer level in TCGA cohorts. A total of 10,283 patients with available survival data were included in survival analysis. The line colors are the same as shown in the panel **d**. Log-Rank *P* values and the median overall survival time (in months) are shown. mo, months. **h** Univariate Cox regression analysis of 9p21 loss for overall survival across 12 TCGA cohorts with frequent 9p21 loss (>10%, see Supplementary Data 3). Numbers within the parentheses indicate the sample size. *P* values were calculated by Cox proportional hazards (PH) regression model. Error bars indicate the estimated 95% confidence interval of the hazard ratio. **i** Representative examples showing that 9p21 loss is associated with significantly shortened overall survival in individual cancer cohorts. The cancer type, molecular subtype, sample size, and Log-Rank *P* values are labeled on each plot. *P* values were calculated by two-sided Log-rank test.

We first analyzed the frequency of 9p21 loss across 33 TCGA (The Cancer Genome Atlas) studies (*n* = 10,435 patients, Supplementary Data 1 and 2) using genomic and transcriptomic datasets from the TCGA program. Among genes mapping to the chromosomal region 9p21.3, *CDKN2A* was most frequently deleted (13.5%), followed by *MTAP* (9.3%) (Fig. 1a, b). In addition to homozygous deletion (HD), loss of heterozygosity (9p21 LOH) due to hemizygous deletion of *CDKN2A* and *MTAP* was observed in 24.6% and 27.8% of cancers, respectively (Fig. 1b). While only subtle changes (vs. wild type) were observed in mRNA expression of *CDKN2A*/*MTAP* in 9p21-LOH tumors, homologous deletion of the genes in tumor cells led to a marked decrease in their mean gene expression levels in bulk tumor tissues (Fig. 1c). *CDKN2A* and *MTAP* were ~100 kb apart on 9p21.3 and commonly co-deleted in human cancers (Fig. 1a). Approximately 9.2% of cancers exhibited homozygous co-deletion of *CDKN2A* and *MTAP*, 3.7% of cancers had *CDKN2A* HD with wildtype or heterozygous *MTAP*, and 0.1% of cancers had *MTAP* HD with wildtype or heterozygous *CDKN2A* (Fig. 1c, e). Twelve cancer types with frequent (>10%) 9p21 loss were selected for subsequent analyses (Fig. 1e and Supplementary Data 3). In 7 out of these 12 cancer cohorts, the frequency of 9p21 loss varied greatly across previously defined molecular subtypes (Fig. 1f and Supplementary Fig. 1).

We next determined the pan-cancer prognostic significance of 9p21 loss (Fig. 1g, h and Supplementary Figs. 2–3). Consistently in multiple TCGA cancer cohorts, patients whose tumors had homozygous co-deletion of *CDKN2A/MTAP* and those who had HD of either gene had significantly shorter survival (Fig. 1g), with no statistical difference observed in the overall survival (OS) time among these three groups (Supplementary Fig. 2). The differences in OS time remained significant in individual cancer cohort and when stratified by previously defined molecular subtypes (Fig. 1i and Supplementary Fig. 3) and after adjustment for potential confounding factors such as SCNA burden and TMB (Supplementary Fig. 4). In addition, although LOH of 9p21 did not lead to massive changes in *CDKN2A/MTAP* expression (Fig. 1c), it conferred significantly shorter OS in comparison with tumors with diploid/wildtype 9p21 (9p21-WT) (Fig. 1g and Supplementary Fig. 5). The genomic loci of type I interferon gene cluster, located ~320 kb upstream of *MTAP* on 9p21 (Fig. 1a), is often co-deleted with *CDKN2A/MTAP* in a subset of cancers. Survival analysis stratified by CNV status of *CDKN2A/MTAP* and type I interferon genes showed no statistical difference in OS time among the groups (Supplementary Fig. 6).

### 9p21 loss correlates with “cold” tumor-immune phenotypes in TCGA Cancers

**Fig. 2:**
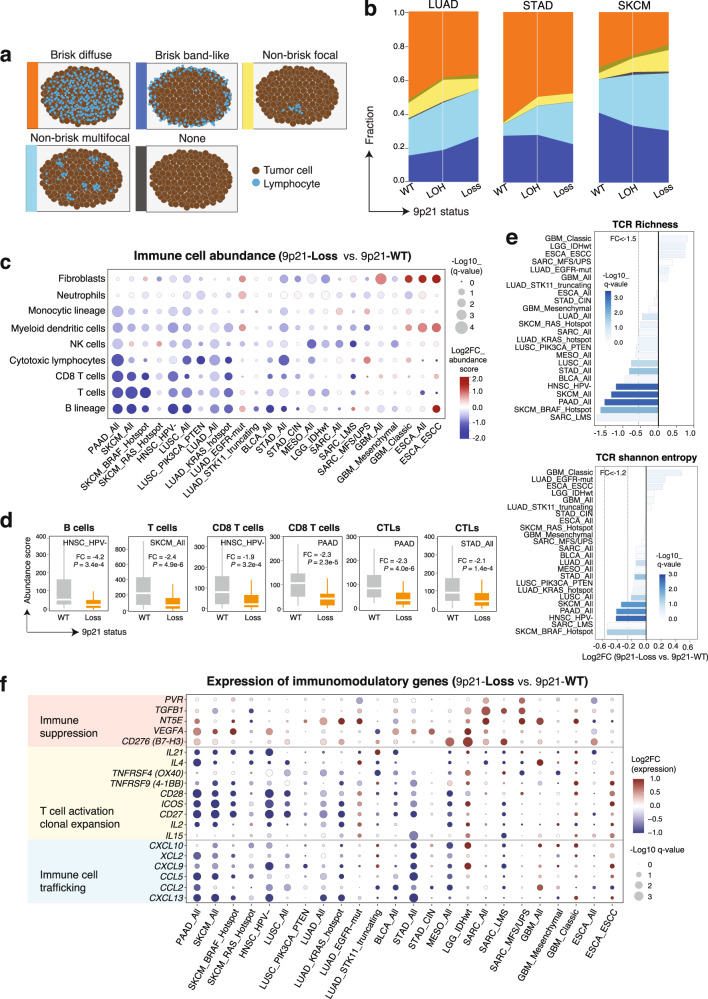
9p21 loss is associated with ‘cold’ tumor-immune phenotypes. **a** Schema showing the patterns of spatial distribution of TILs defined by a previous TCGA study by Saltz et al. **b** Gradient changes in the spatial TILs patterns among 9p21-WT tumors, 9p21-LOH tumors, and 9p21-loss tumors were observed, which corresponded to progressive copy number loss of 9p21. The plots of three representative cancer types are shown (see more details in Supplementary Fig. 7). The FDR *q*-values did not reach significance level at 0.05. **c** 9p21 loss in shaping the immune cell abundance and cell composition in tumor microenvironment. Immune deconvolution was performed by applying MCP-counter^24^ to the bulk RNA-seq data, similarly as described in our recent studies^27,28^. The data is shown for 12 TCGA cohorts (14 molecular subtypes) with frequent 9p21 loss (>10%, see Supplementary Data 3). The bubble plot is drawn using computed log2-transformed fold change (9p21-Loss vs. 9p21-WT) and adjusted *p*-values (FDR q-value). The size of the bubble indicates statistical difference, the bigger the more significant. The color of the bubble indicates change in the immune cell abundance in 9p21-loss tumors (vs. 9p21-WT), with blue denotes depletion and red denotes enrichment. **d** Box plots of representative examples selected from the panel **c** (see more details in Supplementary Fig. 8). *P* values were calculated by two-sided Wilcoxon rank-sum test. Number of samples: B cells in HNSC_HPV-: WT (*n* = 57); Loss (*n* = 135); T cells in SKCM: WT (*n* = 71); Loss (*n* = 112); CD8 T cells in HNSC_HPV-: WT (*n* = 57); Loss (*n* = 135); CD8 T cells in PAAD: WT (*n* = 40); Loss (*n* = 44); CTLs in PAAD: WT (*n* = 42); Loss (*n* = 44); CTLs in STAD: WT (*n* = 163); Loss (*n* = 46). Box, median ± interquartile range; whiskers, 1.5× interquartile range. **e** The richness and diversity of T-cell receptor (TCR) repertoire was decreased in tumors with 9p21 loss in multiple TCGA cohorts. The diversity of TCR repertoire is indicated by the Shannon entropy. The color of the bars indicates the significance level of changes in 9p21-loss tumors (vs. 9p21-WT). **f** Changes in immunomodulatory gene expression in 9p21-loss tumors in comparison with 9p21-WT tumors. A list of 28 immunomodulatory genes (see a full list in Supplementary Data 9) were analyzed and the most significant ones are shown. The color of the bubble corresponds to Log2 fold change in gene expression levels in 9p21-loss tumors (vs. 9p21-WT), with blue denotes decrease and red denotes increase in 9p21-loss tumors.

We next assessed the immunomodulatory effects of 9p21 loss on TME (Fig. 2). According to published data, the spatial organization of tumor-infiltrating lymphocytes (TILs) is an important pathological feature of tumor with prognostic values^22^. A recent pan-cancer study of TIL patterns derived from standard pathology cancer images analysis revealed high degrees of spatial heterogeneity across TCGA cancers^23^. To examine whether 9p21 loss influences TIL density and spatial lymphocytic patterns, we analyzed the TIL map structure patterns characterized by Saltz et al.^23^ (Fig. 2a), which were available for 4337 TCGA cancers from 13 cancer types (Supplementary Data 4 and 5). Our analysis was focused on six cancer types [melanoma (SKCM), bladder (BLCA), pancreatic (PAAD) and gastric (STAD) cancer, lung adeno- (LUAD), and squamous-cell carcinoma (LUSC)] that had frequent 9p21 loss (>10%). Overall, we observed decreased density of TILs (fewer TIL patches) in 9p21-loss tumors compared to 9p21-WT tumors. For example, there was a trend towards decreased proportion of the “brisk diffuse” structural pattern (with diffusely infiltrative TILs scattered throughout at least 30% of the area of the tumor) in 9p21-loss tumors when compared to 9p21-WT tumors, particularly in LUAD, STAD, and SKCM (Fig. 2b), whereas the “non-brisk, multi-focal” pattern (with loosely scattered TILs present in <30% but >5% of the area of the tumor) was increased in SKCM and STAD with 9p21 loss. We also observed a trend towards decreased proportion of the “non-brisk focal” pattern (with TILs scattered throughout <5% but >1% of the area of the tumor) in SKCM with *BRAF* hotspot mutations (Supplementary Fig. 7), and increased proportion of the “brisk band-like” pattern (with TILs mostly localized to the invasive margin of the tumor without entering the tumor body) in 9p21-loss LUAD with somatic *EGFR* or *STK11* mutations (Supplementary Fig. 7). Interesting, we observed gradient changes in the spatial TILs patterns that correspond to progressive copy number loss of 9p21 (from WT to LOH then to HD) in LUAD, STAD, and SKCM (Fig. 2b and Supplementary Fig. 7), supporting the regulatory interplay between 9p21 loss and the spatial immune landscape of cancer.

To further examine the role of 9p21 loss in shaping immune cell abundance and cellular composition, we performed immune deconvolution analysis of the bulk RNA-seq data from TCGA by applying MCP-counter^24^, CIBERSORT^25^, and CIBERSORTx^26^ the same way as described in our recent studies^27,28^. Consistently across most (10/12) cancer types with frequent 9p21 loss (versus 9p21-WT tumors), we observed remarkable decrease in abundance of B cells, T cells, NK cells, T follicular helper cells, memory CD4 T cells, CD8 T-cells, and cytotoxic lymphocytes revealed by both MCP-counter and CIBERSORTx, and such changes remained significant in stratified analysis based on previously defined molecular subtypes (Fig. 2c, d, Supplementary Fig. 8 and Supplementary Data 6). In line with this observation, the leukocyte fractions inferred from DNA methylation signatures^29^, the richness and diversity of immune cell receptor repertoires, in particular the T-cell receptor (TCR) CDR3 repertoire derived from RNA-seq data were decreased significantly in a subset of tumors with 9p21 loss (Fig. 2e and Supplementary Fig. 8). In contrast, in GBM, esophageal carcinoma (ESCA), and *EGFR*-mutant LUAD, we observed distinct features showing significantly increased abundance of myeloid dendritic cells (DCs), neutrophils, and fibroblasts (Fig. 2c) in 9p21-loss tumors and this observation was also supported by independent analysis of marker gene expression. For example, we analyzed myeloid DC subsets including plasmacytoid dendritic cells (pDCs), LAMP3 + DCs, cDC1, and cDC2^30^ and observed an overall decrease in all DC subsets in 9p21-loss tumors, except the cDC2 population, which was enriched in the esophageal squamous-cell carcinoma (ESCA_ESCC) and *EGFR*-mutant LUAD with 9p21 loss (Supplementary Fig. 10a). Consistently, the expression levels of *CD1C*, a marker of cDC2 subset, were significantly increased in 9p21-loss ESCA_ESCC and *EGFR*-mutant LUAD (Supplementary Fig. 10b). Similarly, elevated marker gene expression for fibroblasts and neutrophils in 9p21-loss ESCA_ESCC and *EGFR*-mutant LUAD was also consistent with increased fibroblast abundance inferred by MCP-counter (Supplementary Fig. 10c, d). In addition, we also observed increased fractions of M2-like macrophages in sarcoma (SARC), IDH-wildtype LGG, and GBM (Supplementary Fig. 11). These results indicate that the immunomodulatory effects of 9p21 loss on TME (e.g. depletion of B/T cells or enrichment of myeloid or stromal cells) varied depending on the cancer type.

Moreover, we performed correlation analysis to examine whether 9p21 loss affects PD-L1 expression. A significant positive correlation was observed between gene expression of *CD274* (PD-L1) and *CDKN2A/MTAP* in a subset of TCGA cancers (Supplementary Fig. 12), which indicates decreased PD-L1 expression in cancers with 9p21 loss. Similar analyses were also performed in 9p21-LOH cancers. Compared to the 9p21-WT tumors, we observed a similar trend (significant but with less magnitude) in the changes of immune cell abundance and cellular compositions as seen in the 9p21-loss tumors, such as decreased abundance of B, T, CD8 T, NK cells and cytotoxic lymphocytes, increased fractions of macrophages, and reduced TCR CDR3 repertoire abundance and diversity (Supplementary Fig. 13), as well as decreased PD-L1 expression (Supplementary Fig. 14).

To further understand the biological processes associated with the cold immune phenotypes in cancers with 9p21 loss, gene set enrichment analysis (GSEA) was employed for functional enrichment analyses of tumors in TCGA, focusing on 41 curated immune-related gene sets (Supplementary Data 7). Compared to 9p21-WT tumors, 9p21-loss tumors demonstrated a significant decrease in a number of immune-related pathways including antigen processing and presentation, BCR/TCR signaling, interferon alpha/beta/gamma-mediated immune response, CTLs pathway, and such changes were ubiquitous in 9 out of 12 examined cohorts, with exception of GBM, LGG, and ESCA (Supplementary Fig. 15 and Supplementary Data 8). Consistently, we observed a similar trend (significant but at lower magnitude) in the changes of immune pathway activity in 9p21-LOH tumors (Supplementary Fig. 16).

Finally, to further elucidate the potential mechanisms that govern the cold immune phenotypes in 9p21-loss cancers, we further analyzed the expression of a list of immunomodulatory genes including the cytokines/chemokines regulating immune cell trafficking, T-cell co-stimulatory genes, inhibitory immune checkpoints, genes regulating T-cell activation, expansion and differentiation, and immune suppression (Supplementary Data 9). Among them, 20 genes showed significant differences in their expression levels between the 9p21-loss and 9p21-WT tumors in at least one tumor type/subtype (Fig. 2f). We observed increased expression of 5 immune suppressive genes including *PVR (CD155), TGFB1, NT5E (CD73), VEGFA*, and *CD276 (B7-H3)* in 9p21-loss tumors across multiple tumor types/subtypes when compared to 9p21-WT tumors. The ligand *CD155* expressed on tumor cells can interact with its receptors on immune cells (e.g. T cells, NK cells) and exert an inhibitory signal^31^. Recently, stromal TGFβ signaling has been linked to T-cell exclusion from human and mouse tumors^6,32^. *CD73* encodes an immune checkpoint mediator that is highly expressed on tumor or stromal cells in TME and it functions to catalyze AMP to adenosine, which subsequently impairs anti-tumor T-cell responses^33^. Numerous studies have highlighted a direct or an indirect impact of *VEGFA* on the T-cell-based immunosuppression^34^. B7-H3 inhibits APCs and stimulates Tregs which results in IL-2 suppression. In contrast, the expression of genes regulating immune cell trafficking (e.g., *CXCL13*, *CXCL9, XCL2, CCL5*), T-cell activation and clonal expansion (e.g., *CD27, CD28, ICOS, IL21, IL2*) were massively decreased in 9p21-loss tumors across multiple cancer types/subtypes (Fig. 2f). Among them, *CXCL9* is crucial for recruiting immune T cells into the TME^35^, *XCL2* plays a role in recruitment of DCs^36^, and *CXCL13* can recruit both T cells and B cells into tumor tissues to enhance tumor immunity^35^. Taken together, downregulation of these immunomodulatory factors regulating immune cell recruitment, T-cell activation and clonal expansion alongside with upregulation of the immune suppressive signaling can collectively lead to “cold” immune phenotypes in 9p21-loss tumors.

### 9p21 loss is associated with primary resistance to anti-PD-1/PD-L1 monotherapy: data from eight solid tumor cohorts

Given the evidence that “cold” tumors are unlikely to respond to immunotherapy, we therefore hypothesized that patients whose pre-treatment tumors harboring 9p21 loss may demonstrate primary resistance to immune checkpoint inhibitors and hence manifest low clinical response rates to ICT. To determine the impact of 9p21 loss on clinical outcomes in patients treated with ICT, we performed integrated analyses of the immunogenomic and clinical data of patients receiving ICT (monotherapy) from 8 solid tumor cohorts (>1000 patients) (Table 1 and Supplementary Data 10).

**Table 1:** Summary of the 8 anti-PD-1/L1 ICT trials included in this study.

#	Cohort	Cancer type	Therapy	Cohort size (original)	Cohort size (selected)	ICT monotherapy agents used	Response rate, 9p21-WT group (*n*)	Response rate, 9p21-loss group (*n*)	*P* value	Data source
1	MDA solid tumors	Solid tumors	Anti-PD-1/PD-L1	123	94	Pembrolizumab, Nivolumab, Atezolizumab, CX-072 (anti-PD-L1), FAZ053 (anti-PD-L1)	27%* (8/30)	4% (1/25)	0.03	MDACC
2	High-risk resectable melanoma	Melanoma	Anti-PD-1	22	12	Nivolumab	75% (3/4)	0% (0/5)	0.048	MDACC
3	Metastatic melanoma	Melanoma	Anti-PD-1	144	58	Pembrolizumab, Nivolumab	61% (11/18)	28% (4/14)	0.087	Liu et al.
4	Metastatic melanoma	Melanoma	Anti-PD-1	120	41	Pembrolizumab, Nivolumab	69% (9/13)	31% (4/13)	0.23	Gide et al.
5	Unresectable or advanced melanoma	Melanoma	Anti-PD-1	65	23	Nivolumab	40% (4/10)	11% (1/9)	0.30	Riaz, et al.
	Combined melanoma cohort (#2-#5)	Melanoma	Anti-PD-1	351	134	Pembrolizumab, Nivolumab	60% (27/45)	22% (9/41)	0.0004	
6	MSKCC advanced NSCLC	Lung cancer	Anti-PD-1/PD-L1	240	151	n/a	29%** (40/137)	7% (1/14)	0.048	Rizvi, et al.
7	MDA metastatic urothelial cancer (mUC)	Urothelial cancer	Anti-PD-1/PD-L1	86	80	Pembrolizumab, Atezolizumab	29% (17/58)	9% (2/22)	0.009	MDACC
8	mUC IMvigor210 trial	Urothelial cancer	Anti-PD-L1	358	298	Atezolizumab	32% (40/124)	12% (15/127)	7.0E-05	Mariathasan et al.
Total				1098	757		34% (132/394)	12% (28/229)	<0.0001	

*P* values was calculated using two-tailed Fisher’s exact test. Refer to Supplementary Data 6 for further details.

*MDACC* MD Anderson Cancer Center.

*Calculated for the ICT responsive cohort; **response was defined by the original study.

**Fig. 3:**
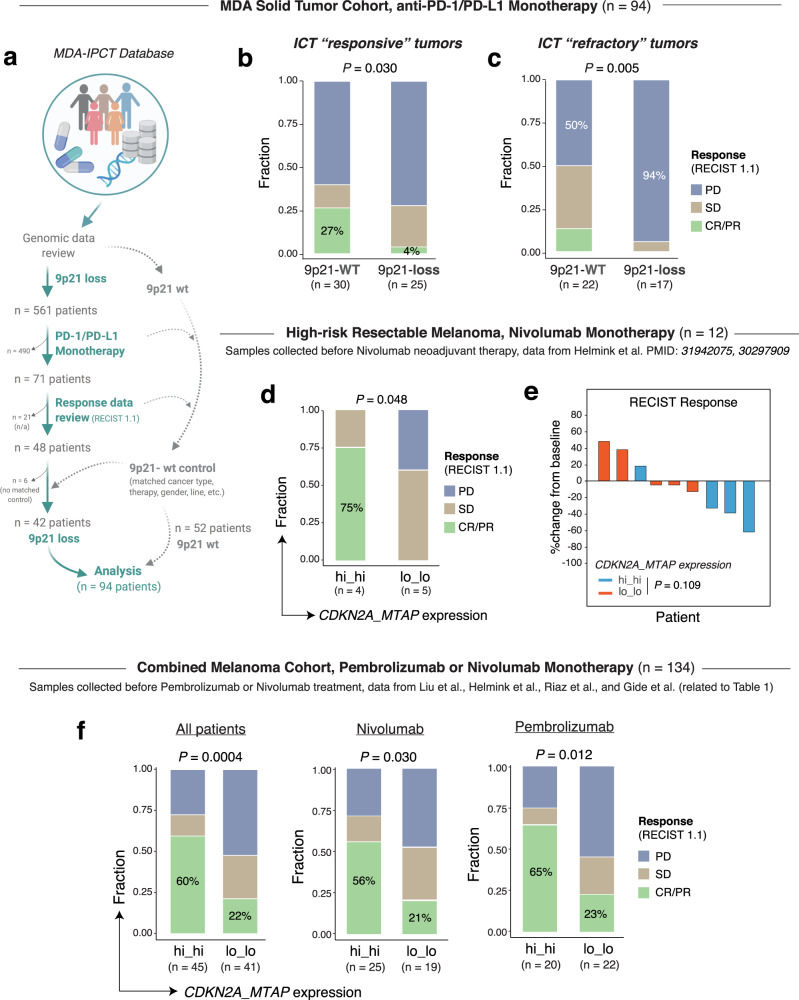
9p21 loss is associated with immune resistance to anti-PD-1/L1 monotherapy in solid tumors. **a**–**c** the MDA (MD Anderson Cancer Center) solid tumor cohort (*n* = 94 patients). **a** Schematic view of the information collection and analysis flow. **b** 9p21 loss is associated with lack of response to anti-PD-1/L1 monotherapy in the ICT “responsive” tumor cohort. The response rates (percentages of CR/PR) were compared between the two groups. **c** 9p21 loss is associated with disease progression following anti-PD-1/L1 monotherapy in the ICT “refractory” tumor cohort. The progression rates (percentages of PD) were compared between the two groups. *P* values were calculated by two-tailed Fisher’s exact tests. **d**, **e** the high-risk resectable melanoma cohort from Helmink et al. *hi_hi*, tumors with *mRNA* expression levels of both *CDKN2A* and *MTAP* above the group median and *lo_lo*, tumors with expression levels of both genes below the group median. **d** Comparison of the response rates (percentages of CR/PR) to ICT between the hi_hi and lo_lo groups. *P* values were calculated with two-sided Fisher-exact test. **e** Waterfall plot showing the RECIST response calculated based on the percentage of change in tumor volume relative to baseline. *P* value was calculated using the two-sided Mann–Whitney *U* test. **f** The combined melanoma cohort from 4 studies (see Table 1 and Supplementary Data 10 for details). The response rates were compared between the hi_hi and lo_lo groups for all patients together (left), and in individual patient subpopulations receiving nivolumab (middle) and pembrolizumab (right), respectively. *P* values were calculated using the two-tailed Fisher’s Exact tests.

First, we screened the clinical trial database of Institute for Personalized Cancer Therapy (IPCT) at MD Anderson Cancer Center and identified 561 patients whose pre-treatment cancers had 9p21 loss (Fig. 3a), which was determined by the SCNA profiles derived from the FoundationOne CDx panel and/or MTAP protein expression by immunohistochemistry (IHC). Among these patients, 71 received anti-PD-1/PD-L1 monotherapy and 48 had response data available for review. Six patients were further filtered out due to rare cancer types, leading to a group of 42 patients with 9p21 loss. Concurrently, we identified another group of patients (*n* = 52) who received anti-PD-1/PD-L1 monotherapy and whose pre-treatment cancers were 9p21-WT with largely matched cancer type, gender and therapy as the control cohort, resulting in a solid tumor cohort consisting of 94 patients (Fig. 3a and Supplementary Data 11) for subsequent analysis. The clinical responses were assessed by the Response Evaluation Criteria in Solid Tumors (RECIST) version 1.1 guideline. We categorized the solid tumors into ICT “responsive” (including melanoma, lung, renal, head and neck, and esophageal cancers that have PD-1/PD-L1 therapy already FDA-approved) and ICT “refractory” (including breast, pancreatic, and prostate cancer, and glioblastoma that have no FDA-approved ICT) cohorts, and performed comparative analysis between the 9p21-WT and 9p21-loss tumors within each cohort. For the cohort of ICT “responsive” tumors, 27% of patients in the control group (with 9p21-WT tumors) achieved complete or partial response (CR/PR), whereas the response rate dropped to 4% (>6-fold decrease, *P* = 0.030) in patients whose pre-treatment tumors harboring 9p21 loss (Fig. 3b). For ICT “refractory” tumors, the disease progression (PD) rate increased 1.9-fold (94% vs. 50%, *P* = 0.005) in patients whose pre-treatment tumors had 9p21 loss compared to those with 9p21-WT tumors (Fig. 3c).

We next assessed the impact of 9p21 loss on clinical responses to ICT in four published melanoma cohorts: the high-risk resectable melanoma cohort from Helmink et al.^27^, the two metastatic melanoma cohorts from Liu et al.^37^ and Gide et al.^38^, respectively, and the unresectable or advanced melanoma from Riaz et al.^39^. (Fig. 3d, e and Supplementary Figs. 17–18; and Supplementary Data 10). As the transcriptomic data were available and easily accessible for all these cohorts, we inferred 9p21 status based on expression levels of *CDKN2A* and *MTAP*: tumors with expression levels of both *CDKN2A* and *MTAP* below their group medians were classified as “lo_lo” *(CDKN2A_MTAP*: lo_lo*)*, and tumors with expression levels of both genes above their group medians were classified as “hi_hi” *(CDKN2A_MTAP*: hi_hi*)*. For all four cohorts, patients received ICT as monotherapy (without prior history of ICT) and with immunogenomics data generated on pre-treatment tumors were selected (Supplementary Data 10). For the high-risk resectable melanoma cohort with limited sample size^27^, we observed significant differences in the response rates to Nivolumab monotherapy between the hi_hi and lo_lo groups (Fig. 3d). The data showed a trend towards greater RECIST response in the hi_hi group (Fig. 3e). Consistently, similar trend was observed in three additional melanoma cohorts (Supplementary Figs. Supplementary Data 17–18). Patients whose pre-treatment tumors had low expression of both *CDKN2A* and *MTAP (*lo_lo*)* showed on average 2.7-fold lower response rate to ICT, compared to that observed in the hi_hi group. To increase the statistical power, we further examined the impact of 9p21 loss on clinical response to ICT in melanoma patients by combining these four datasets together, similarly as described in a previous study^40^. In this combined melanoma cohort (*n* = 134), 22% of patients in the lo_lo group achieved CR/PR following pembrolizumab or nivolumab monotherapy, and the response rate was 2.7-fold lower in the hi_hi group (60%, *P* = 0.0004) (Fig. 3f, left). The difference in response rate remained significant in individual pembrolizumab or nivolumab subpopulations (Fig. 3f, right).

**Fig. 4:**
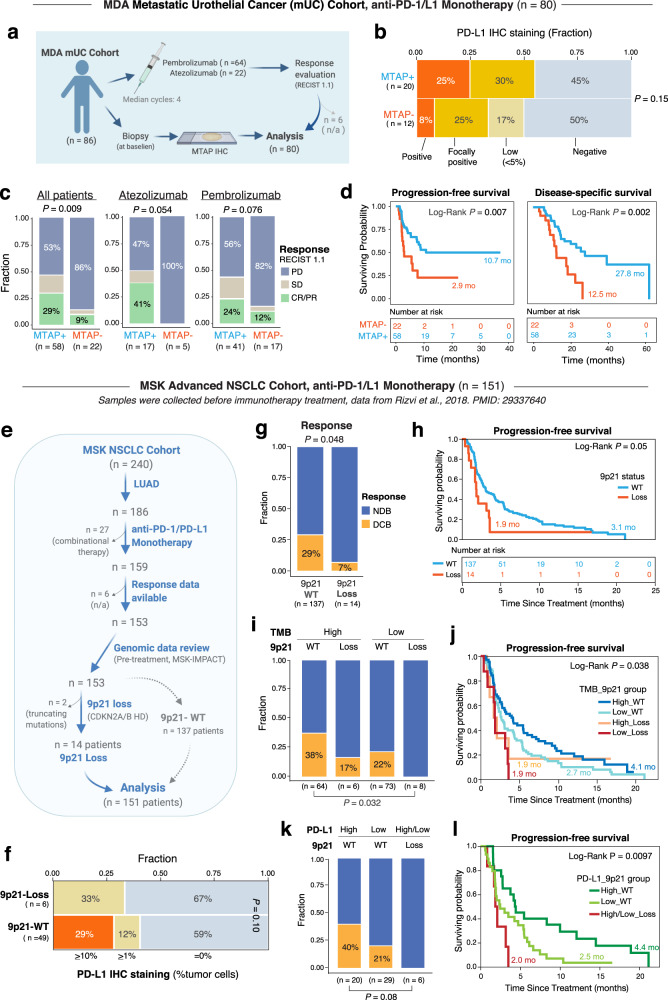
9p21 loss is associated with immune resistance to anti-PD-1/L1 monotherapy in large metastatic urothelial cancer (mUC) and advanced non-small-cell lung cancer (NSCLC) cohorts. **a**–**d** the MDA mUC cohort. A total of 86 mUC patients who received either pembrolizumab or atezolizumab monotherapy were included and 80 patients with available response and MTAP IHC data were taken into subsequent analyses. Samples were collected prior to ICT. **a** Schematic view of the information collection and analysis flow. **b** Decreased trend of PD-L1 stain positivity in MTAP-negative tumors. Colors in this plot indicates the four categories of PD-L1 IHC staining results. **c** MTAP loss is associated with primary resistance to ICT and disease progression following pembrolizumab or atezolizumab monotherapy. *P* values were calculated using the two-tailed Fisher’s Exact tests by comparing the rates of disease progression (percentages of PD) between two groups. **d** MTAP loss is associated with worse progression-free survival (PFS) and disease-specific survival (DSS) in mUC patients received pembrolizumab or atezolizumab monotherapy. **e**–**l** the MSK NSCLC cohort from Rizvi et al.^17^. **e** Schematic view of the information collection and analysis flow. A total of 151 LUAD patients received PD-1/L1 as monotherapy with available genomic and response data were included in subsequent analyses. **f** Decreased trend of PD-L1 positivity in tumors with 9p21 loss. Colors in this plot indicates the categorized PD-L1 IHC staining results. **g** 9p21 loss is associated with a lower rate of DCB (response defined and shorter PFS (**h**). DCB, durable clinical benefit, defined as complete/partial response or stable disease that lasted >6 months by the original study^17^ (the detailed classification of CR, PR, SD, PD were not available). NDB no durable benefit. Integration of 9p21 status with TMB (**i**, **j**) or PD-L1 expression (**k**, **l**) in patient stratification for response and PFS. TMB tumor mutation burden. PD-L1 expression was measured by immunohistochemistry staining by the original study. *P* value in panel H was calculated with two-sided Log-rank test. *P* values in panels **g**, **i** and **k** were calculated by two-tailed Fisher’s exact tests.

We next sought to evaluate the validity of these findings across additional cancer types in a large independent series. We first assessed the metastatic urothelial cancer (mUC) cohort from MD Anderson Cancer Center, which is composed of 86 patients who received pembrolizumab (*n* = 64) or atezolizumab (*n* = 22) monotherapy (Fig. 4a and Supplementary Data 12). Eighty of 86 patients with available response and follow-up data were considered in subsequent analysis. Due to data availability, the protein level positivity of MTAP was used as a surrogate biomarker of 9p21 loss based upon the observations that p16 does not stain well by IHC; TCGA genomics data showed that all of the *MTAP*-HD bladder cancers were also *CDKN2A*-HD (Fig. 1d, e); and HD of *MTAP* led to a marked decrease in its mean gene expression levels in bulk tumor tissues (Fig. 1c) which was further reflected at the protein level. The MTAP protein level positivity status was determined through a CLIA-certified IHC test. MTAP positivity was performed on the baseline biopsies and based on which, patients were stratified into MTAP positive (MTAP+, *n* = 58) and MTAP negative (MTAP−, *n* = 22) groups. PD-L1 IHC staining in tumor cells was performed in a subset of patients, showing a trend of decreased fraction of PD-L1 positivity in tumors of the MTAP- group (Fig. 4b). Overall, 9% (2/22) of patients in the MTAP− group achieved CR/PR following pembrolizumab or atezolizumab monotherapy, which was more than three times lower than that observed in the MTAP+ group (29%, *P* = 0.078). On the contrary, the fraction of patients that experienced disease progression was significantly increased in the MTAP− group as compared to that in the MTAP+ group (86% vs. 53%, Fisher’s Exact test two-sided, *P* = 0.009) (Fig. 4c left and Supplementary Fig. 19a). The difference in response and disease progression rates were marginal in individual pembrolizumab or atezolizumab cohort (Fig. 4c middle and right and Supplementary Fig. 19b). Furthermore, patients of the MTAP- group exhibited significantly reduced survival for both the progression-free survival (PFS) and disease-specific survival (DSS), compared to those of the MTAP+ group (Fig. 4d), and the difference in survival probability remained significant in individual pembrolizumab and atezolizumab cohorts (Supplementary Fig. 19c, d).

The association between 9p21 loss and lack of response to ICB was further corroborated in a large cohort of non-small-cell lung cancer (NSCLC) patients from Rizvi et al.^17^. Among 240 patients, 151 were LUADs, received PD-1/PD-L1 monotherapy and had both genomic and response data available for subsequent analysis (Fig. 4e and Supplementary Data 13). The copy number status of 9p21 was determined using genomic data from the MSK-IMPACT panel^41^. PD-L1 protein expression on tumor cells was available for 55 patients, of whom 33 (60%) had negative PD-L1 staining. PD-L1 expression at a high intensity (≥10%) was present in 29% of samples in 9p21-WT tumors, however, it was not detected in 9p21-loss tumors (Fig. 4f). Compared to the 9p21-WT group, patients in the 9p21-loss group showed more than 4-fold decrease (7% vs. 29%, likelihood-ratio chi-squared test, *P* = 0.048) in the rate of durable clinical benefit (DCB), defined by the original study^17^ (Fig. 4g) and reduced PFS (Fig. 4h). It was shown in the original study that patients with low TMB (lower than the group median) had a 20% rate of DCB, compared to a 36% rate with high TMB^17^ (Supplementary Fig. 20a), whereas the presence of 9p21 loss was associated with a lower rate of DCB (Fig. 4i), independent of the TMB level (Supplementary Fig. 20b): patients with high TMB and 9p21 loss had a 17% rate of DCB and none of the patients (0/8) with low TMB and 9p21 loss had DCB. Similarly, patients with PD-L1 negativity had a 18% rate of DCB, compared to a 36% rate with PD-L1 positivity (≥1% expression) (Supplementary Fig. 20c), whereas none of the patients (0/6) with 9p21-loss tumors, irrespective of PD-L1 expression levels, had DCB (Fig. 4k). Given the fact that 10% of patients with low TMB and PD-L1 negative staining achieved DCB in the original study (Supplementary Fig. 20d), these results suggest that 9p21 loss may serve as a biomarker that can compensate for other biomarkers including PD-L1 expression and TMB level, particularly in identifying NSCLC patients that are unlikely to benefit from PD-1/PD-L1 monotherapy. In line with this, we showed that patients whose pre-treatment tumors had 9p21 loss, irrespective of TMB level or PD-L1 expression, had a shorter PFS (Fig. 4j, l), indicating the potential value of 9p21 loss as a biomarker for poor outcome in the setting of PD-1/PD-L1 monotherapy.

**Fig. 5:**
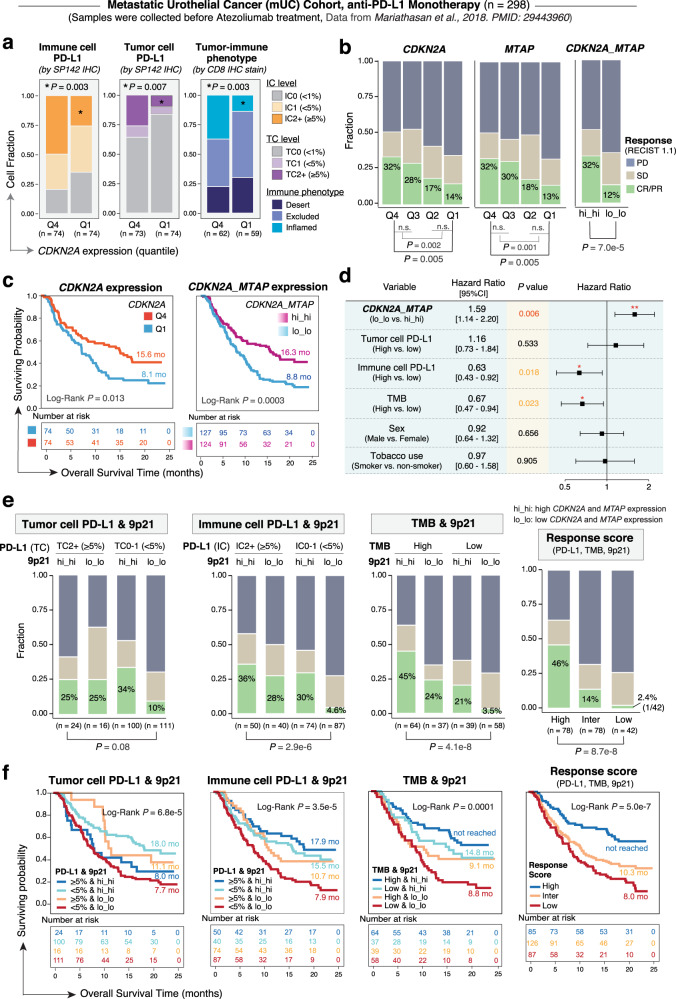
Validation of the translational impact of 9p21 loss on ICT in large-scale metastatic urothelial cancer (mUC) cohort. Patients were from the IMvigor210 trial investigating Atezolizumab (anti-PD-L1) blockade in the mUC cohort (*n* = 298 patients). Pre-treatment samples were collected for bulk RNA-seq and immune profiling and the data was downloaded from a published study from Mariathasan et al.^6^. **a** The proportions of immune (left) and tumor (middle) cells that were positive for PD-L1 staining (by SP142 immunohistochemistry) were significantly lower in tumors with decreased *CDKN2A* expression (top and bottom quantiles, Q4 vs. Q1), and the fraction of “inflamed” immune phenotype (right) was also significantly lower in low (Q1) than those with high (Q4) *CDKN2A* expression. The immune phenotypes were defined by CD8 IHC staining by the original study. *P* values were calculated by two-tailed Fisher’s exact tests. **b** (left, middle) The *CDKN2A* and *MTAP* expression levels, respectively, were stratified into decreasing quantiles, and the response rates (percentages of CR/PR) decreased significantly with decreasing *MTAP/CDKN2A* expression. (right) *CDKN2A* and *MTAP* co-expression patterns can better stratify patients for response (hi_hi, tumors with *mRNA* expression levels of both *CDKN2A* and *MTAP* above the group median, *n* = 124, and lo_lo, tumors with expression levels of both genes below the group median, *n* = 127), and overall survival (**c**) following PD-L1 blockade by Atezolizumab. *P* values in panel **b** were calculated by two-tailed Fisher’s exact tests. *P* value in panels **c** was calculated with two-sided Log-rank test. **d** Multivariable Cox regression analysis showing that 9p21 loss was a strong prognosticator of short survival, independent of other variables listed. Cox proportional hazards (PH) regression model was used to calculate the Hazard Ratio (HR), the 95% confidence interval (95%CI) and *P* values. Error bars indicate the estimated 95%CI of the HR. Number of samples: *CDKN2A*_*MTAP*, lo_lo (*n* = 127), hi_hi (*n* = 124); Tumor-cell PD-L1, High (*n* = 42), Low (*n* = 255); Immune cell cell PD-L1, High (*n* = 102), Low (*n* = 195); TMB, High (*n* = 120), Low (*n* = 114); Sex, Male (*n* = 233), Female (*n* = 65); Tobacco use, Smoker (*n* = 32), and non-smoker (*n* = 266). **e** 9p21 status can compensate PD-L1 expression and TMB in identifying the responders and non-responders to Atezolizumab and showed significant correlates with survival (**f**). The cut off of PD-L1 expression was 5% as suggested by the original study, and the median value of TMB was used to split patients into TMB-high and TMB-low groups. Patients without PD-L1 IHC data (*n* = 1) and those without TMB data (*n* = 64) were excluded from corresponding analysis. Response scores were calculated by incorporating three factors (9p21, PD-L1 expression on immune cells, TMB), which stratified patients into three groups, with high^3,4^, intermediate^1,2^, and low(0) response score. Log-Rank *P* values and the median overall survival time (in months) are shown. mo months. *P* values in panel **e**, **f** were calculated by two-tailed Fisher’s exact tests.

Finally, these findings were replicated in an additional large-scale phase-2 trial (IMvigor210) investigating PD-L1 blockade (atezolizumab) in metastatic urothelial cancer (mUC) patients. RNA-seq and PD-L1 staining data generated on the pre-treatment tumors with overall response data available (*n* = 298, Supplementary Data 14) were downloaded from the prior published report by Mariathasan et al.^6^. 9p21 status was inferred based on transcriptional expression levels of both *CDKN2A* and *MTAP*, the same as described above in the melanoma cohorts. We stratified *CDKN2A/MTAP* expression into increasing quartiles and first examined whether changes in *CDKN2A/MTAP* expression were associated with TME immune cell composition, PD-L1 expression on immune and tumor cells and the immune phenotypes. The abundance of CD8 T-cells, NK cells, cytotoxic lymphocytes inferred by MCP-counter (Supplementary Data 15), and the relative proportion of M1-like macrophages estimated by CIBERSORT (Supplementary Data 16) was significantly lower in tumors with low (Q1) than those with high (Q4) *CDKN2A* expression (Supplementary Fig. 21a). *PD-L1* mRNA expression was downregulated in tumors of the lo_lo group (Supplementary Fig. 21b). PD-L1 protein expression levels on both immune and tumor cells measured by IHC staining were decreased in the lo_lo group, especially in tumors with low (Q1) *CDKN2A* expression (Fig. 5a, left). In addition, the fraction of “inflamed” immune phenotype was significantly lower in low (Q1) than those with high (Q4) *CDKN2A* expression (Fig. 5a, right).

We next examined how decreasing cut points of *CDKN2A/MTAP* expression affects response rates to anti-PD-L1 (atezolizumab) treatment. When *MTAP/CDKN2A* expression levels were stratified into decreasing quantiles, CR/PR rates dropped significantly with diminished levels of *MTAP/CDKN2A* expression (Fig. 5b, left and middle) and a composite of *CDKN2A* plus *MTAP* expression further segregated patients by their response rates (hi_hi: 32% versus lo_lo: 12%, *P* = 7.0e-5) (Fig. 5b, right), as well as survival in the setting of atezolizumab therapy (Fig. 5c, Supplementary Fig. 21d). Multivariable Cox regression analysis showed that 9p21 loss was a strong prognosticator of short survival, independent of other variables such as immune cell PD-L1 expression or TMB levels (Fig. 5d).

We further assessed whether 9p21 loss can synergize with D-L1 expression or TMB in identifying non-responders to atezolizumab (Fig. 5e). Patients with high PD-L1 expression (≥5%, IC2+) on immune cells and high *CDKN2A/MTAP* expression (hi_hi) in their pre-treatment tumors had the best response rate (36%), and those with low PD-L1 expression (<5%, IC0/1) on immune cells but high *CDKN2A/MTAP* expression in their pre-treatment tumors also responded well (30%), whereas those with low PD-L1 expression on immune cells and low *CDKN2A/MTAP* expression exhibited the lowest response rate (4.6%, 4/87), which was 7.8-fold lower than that in the first group −high immune cell PD-L1 expression and high *CDKN2A/MTAP* expression in tumor cells, and 3.7-fold lower than the rate in PD-L1-low patients that stratified solely based on the immune cell PD-L1 expression. Similarly, patients with low TMB and low *CDKN2A/MTAP* expression in their pre-treatment tumors had only a 3.5% (2/58) rate of CR/PR, which was 12.9-fold lower than that in the best group (high TMB and high *CDKN2A/MTAP* expression, 45%), and 3.4-fold lower than the rate in TMB-low patients stratified solely based on TMB levels.

To evaluate the translational relevance of the findings, a logistic regression model was built with these three factors (9p21, PD-L1 expression on immune cells, TMB) and tested in the mUC cohort from Mariathasan et al.^6^, which showed marginal significance for all three variables (*P* = 0.05, 0.06, 0.05, respectively). We then built a “response score” incorporating these factors and stratified patients into 3 groups, with high^3,4^, intermediate^1,2^, and low(0) response score. Our data demonstrated that only 2.4% (1/42) of patients with a response score = 0 had a response, whereas 14% (11/78) of patients with a response score of 1–2 and 46% (36/78) of patients with a response score of 3–4 achieved CR/PR, respectively (*P* = 8.7e-8, Fig. 5e, right). This model allowed us to stratify patients into a bottom group (response score = 0) that composed of patients who were nearly exclusive non-responders (CR/PR: 2.4%, 1/42), a middle group (response score = 1–2) that exhibited ~6-fold (CR/PR: 14%, 11/78) higher response rate than patients in the bottom group, and a top group (response score = 3–4) that showed ~20-fold (CR/PR: 46%, 36/78) higher response rate than patients in the bottom group, i.e., composed of patients who derived the greatest therapeutic benefit from ICT.

In accordance with this, survival analysis showed that patients with low *CDKN2A/MTAP* expression in their pre-treatment tumors had poor outcome, demonstrating that our proposed composite of 9p21 status plus PD-L1 or TMB can better stratify patients (Fig. 5f).

Taken together, our analyses of 757 patients across different tumor types (Table 1) demonstrate that 9p21 loss is associated with poor clinical response to ICT in the group of patients who would otherwise already have poor prognosis, further highlighting the urgent needs of identifying other potential therapeutic targets, which is explored in the following section.

### Therapeutic vulnerabilities and potential targets in tumors with 9p21 loss

**Fig. 6:**
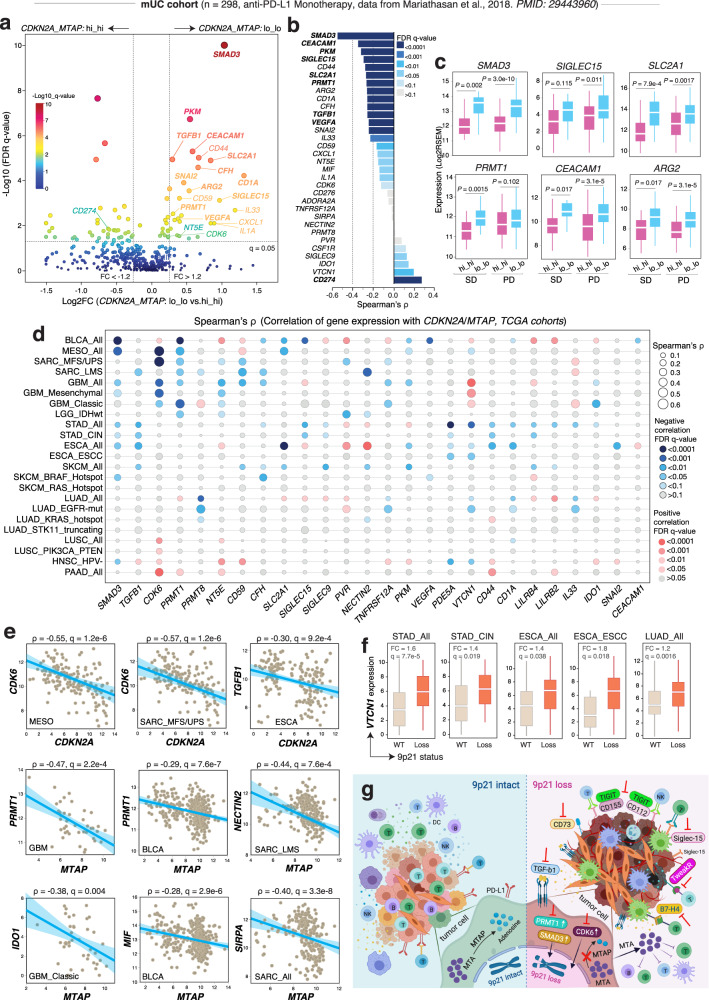
Therapeutic vulnerability and potential immunotherapy targets in tumors with 9p21 loss. **a**–**c** Identification of potential immunotherapy targets in the mUC cohort from Mariathasan et al.^6^. **a** Differentially expressed immune-related genes in the *CNKN2A_MTAP: lo_lo* tumors. A curated list of ~500 genes (including known and emerging viable immunomodulatory targets and other druggable targets of cancer and cytokines, see Supplementary Data 17 for the complete list) were analyzed the most significant genes that upregulated in the lo_lo group (except CD274 which was downregulated) were labeled on the plot. Two vertical lines indicate gene expression fold change (lo_lo vs. hi_hi) >1.2 and <−1.2, respectively, and the horizontal line indicates the adjusted *P* value (FDR *q*-value) of 0.05. *P* values were calculated by two-sided Wilcoxon rank-sum test. The color of the dot represents the FDR (*q*-value) levels. **b** Spearman correlation analysis identified potential immunotherapy targets that were reversely correlated with *CDKN2A/MTAP* expression, i.e. upregulated in tumors with low *CDKN2A/MTAP* expression. The Spearman correlation ecoefficiency is shown on the x axis and the bars are color coded by FDR *q*-value. Two vertical lines indicate Spearman’s *ρ* < −0.2 and <−0.4, respectively. The color of the bar represents the FDR (*q*-value) levels. **c** Box plots showing representative genes displayed in panels **a** and **b**. The expression levels were compared in the pre-treatment tumors between the lo_lo and hi_hi groups and stratified by patient’s response status (SD and PD). Sample size: SD, hi_hi (*n* = 24), lo_lo (*n* = 18); PD, hi_hi (*n* = 57), and lo_lo (*n* = 67). *P* values were calculated by two-sided Wilcoxon rank-sum test. Box, median ± interquartile range; whiskers, 1.5× interquartile range. **d**–**f** Identification of potential immunotherapy targets in the TCGA cohorts. **d** Spearman correlation of gene expression with *CDKN2A/MTAP* across 12 TCGA cohorts (14 molecular subtypes) with frequent 9p21 loss (>10%, see Supplementary Data 3). The size of the bubble represents the correlation levels. The color of the bubble represents the FDR levels. Red: positive correlation. Blue: negative correlation. **e** Scatter plots showing representative genes displayed in the panel **d**. The cancer type and molecular subtype, Spearman correlation ecoefficiency and FDR *q*-value are labeled on each plot. Error bands indicate the estimated interval of correlation level. **f** Box plot showing *VTCN1 (B7-H4)* expression between the 9p21-loss and 9p21-WT groups. Sample size: STAD_All, WT (*n* = 171), Loss (*n* = 50); STAD_CIN, WT (*n* = 45), Loss (*n* = 39); ESCA_All, WT (*n* = 32), Loss (*n* = 64); ESCA_ESCC, WT (*n* = 12), Loss (*n* = 52); LUAD_All, WT (*n* = 162), and Loss (*n* = 87). Box, median ± interquartile range; whiskers, 1.5× interquartile range. **g** Schema summaries the immunological modulation of 9p21 to the TME and potential immunotherapy targets identified in this study.

In an attempt to develop alternative strategies to overcome ICT resistance and poor clinical outcomes in patients with 9p21-loss cancers, we explored potential druggable targets by mining the available biological datasets. We first analyzed bulk RNA-seq data generated on the pre-treatment tumors from patients in the mUC cohort by Mariathasan et al.^6^. Differential gene expression (DEG) analysis focusing on a curated list of ~500 genes (including known and emerging viable immunomodulatory targets and other druggable targets of cancer, see Supplementary Data 17) identified 26 significantly upregulated genes (expression FC > 1.2 and adjusted *P*-value < 0.05) in tumors with low *CDKN2A/MTAP* expression (lo_lo tumors) (Fig. 6a and Supplementary Data 18). Among them, some are promising therapeutic targets in cancer immunotherapy such as TGF-β signaling (*TGFB1, SMAD3*)^6,42,43^, Siglec-15 (*SIGLEC15*)^44,45^, *CEACAM1*^46,47^, *VEGFA*^48^, and other druggable targets such as *PRMT1*^49–51^, and pyruvate kinase M2 (*PKM*)^52–54^, and glucose transporter 1 (*SLC2A1/GLUT1*)^55,56^. Consistently, we observed strong negative correlations between mRNA expression of *MTAP/CDKN2A* and many of these upregulated genes (Fig. 6b and Supplementary Data 19), and interestingly, these genes were significantly upregulated in the pre-treatment tumors of the lo_lo group that progressed following atezolizumab therapy (Fig. 6c).

To examine whether these potential targets are widely applicable to other cancer types with 9p21 loss, we performed a pan-cancer analysis of these ~500 genes (Supplementary Data 20) focusing on 12 cancer types that showed frequent 9p21 loss (Supplementary Data 3). We found that some druggable targets such as TGF-β signaling, *CDK6, PRMT1*, *Siglect-15*, *CD73* (*NT5E*), glucose transporter 1 (*SLC2A1/GLUT1*), TIGIT pathway *CD155/CD112* (*PVR/NECTIN2*), PKM, the TWEAK receptor Fn14 (*TNFRSF12A*)^57,58^, and *VTCN1* (B7-H4) were present in multiple cancer types, demonstrating significant inverse correlation with *CDKN2A/MTAP* expression (Fig. 6d, e and Supplementary Data 19) and/or upregulated in tumors with 9p21 loss (Fig. 6f), while some others were tumor-type specific such as *CEACAM1*, *IDO1*, and *SIRPa*. These results indicate that tumors with 9p21 loss should be treated as a heterogenous group and necessitate tailored therapy, due to the differential expression of these druggable targets across distinct tumor types/subtypes.

## Discussion

9p21 loss is one of the most frequent SCNAs observed in human cancers^19–21^. However, the molecular consequences of 9p21 loss, in particular, its role in modulating the tumor-immune microenvironment and consequently, patient response to ICT, are not fully characterized. In this study, we systematically characterized 9p21 loss in large independent datasets from TCGA and 8 clinical trials of immune checkpoint inhibitors across various cancer types. High-dimensional integration of the molecular, immunogenomic, and clinical data allowed us to elucidate how 9p21 loss shapes the anti-tumor-immune response and influences efficacy of ICT. We demonstrated that 9p21 loss is associated with “cold” tumor-immune phenotypes, primary resistance to immune checkpoint inhibitors and poor outcomes following ICT. Primary resistance to ICT is a significant barrier to efficacy in current treatment of cancer^2^, and elucidation of the molecular cues may thus facilitate the design of effective therapeutic interventions to improve clinical outcomes.

We demonstrate that 9p21-loss tumors were immunologically “cold”, exhibiting much lower densities of TILs, reduced abundance of tumor-infiltrating immune cells of both the adaptive (e.g. B and T cells) and innate (e.g. NK cells) immune systems, altered spatial TILs patterns, shifted immune cell compositions, impaired TCR, antigen presentation, interferon signaling, and a lower rate of PD-L1 positivity. Such alterations in TME were consistently observed across 9 out of 12 tumor types analyzed in this study, suggesting a global phenomenon in the setting of data heterogeneity. The “cold” immune phenotypes in 9p21-loss cancers were likely attributed to both the downregulation of factors regulating immune cell recruitment, T-cell activation, clonal expansion, and the upregulation of immune suppressive pathways. For example, expression of *CXCL13, CXCL9, XCL2, CCL5*, cytokines regulating immune cell recruitment and *CD27, CD28, ICOS, IL21*, the stimulatory signaling of T-cell activation and clonal expansion were significantly decreased, whereas expression of *PVR (CD155), TGFB1, NT5E (CD73), VEGFA, CD276 (B7-H3)* the immune suppressive genes were upregulated in 9p21-loss cancers. At the metabolic level, the association between 9p21 loss and cold immune phenotypes is also supported by several lines of experimental evidence. For example, it has been shown in cancer cell lines that *MTAP* loss (present in >99% of tumors with *CDKN2A* loss) results in an accumulation of the metabolite 5′-methylthioadenosine (MTA) in tumor cells and the extracellular environment. MTA is a structural analog of the negative immune regulator adenosine that acts through the adenosine A_2B_ receptor (*ADORA2B*)^59–61^. Published reports indicate that tumor-derived MTA metabolite acts to suppress T-cell functions^62^ and to inhibit arginine methylation of STAT1, thus leading to diminution of the biological responses to interferons (IFNs)^63^, which is essential for T-cell function and PD-L1 expression. Other than impaired T-cell function and interferon signaling, *MTAP* loss has been shown to promote the immunosuppressive alternative activation of M2-like macrophages in GBM cell lines^64^. In addition, *CDKN2A* deletion leads to constitutive CDK4/6 activity, which is although best known for its function in promoting cell cycle progression, emerging evidence indicates its roles in regulating T-cell biology^65^. CDK4/6 have been shown as master regulators of the immune resistance program in melanoma and inhibition of CDK4/6 represses the resistance program and improves responses to ICT in vivo^66^. Taken together, these various mechanistical insights highlight an intimate link between 9p21 loss and unfavorable reprogramming of the TME.

Another important finding of this study is that 9p21 loss is strongly associated with primary resistance to ICT. Despite utilizing different approaches to infer 9p21 copy number status limited by the availability of genomic, transcriptomic, or IHC data, our integrated analysis of the immunogenomic and clinical data from 8 clinical trials (~800 patients) with anti-PD-1/PD-L1 therapy consistently show a compelling relationship between 9p21 loss and reduced clinical response rates. The response rate to ICT was decreased significantly in large-scale independent studies such as the mUC, advanced NSCLC, and miscellaneous solid tumor cohorts, as well as the combined melanoma cohorts. Compared to 9p21-WT tumors, 9p21-loss tumors exhibited on average a 2.8-fold lower response rate to ICT. Notably, 9p21 loss may serve as a potential biomarker that synergize with PD-L1 expression and TMB (outperforms PD-L1 or TMB alone or in combination), in identifying both patients who have great potential to benefit from ICT and the likely non-responders. The ability of stratifying patients to match a specific therapy through clinical biomarkers has several important implications encompassing improved overall therapeutic efficacy, reduction of economic burden. What’s more importantly, identification of potential non-responders prior to ICT can guide early and more effective interventions in these patients by targeting other potential druggable vulnerabilities of the tumors.

9p21 loss correlates with the worst prognosis across both TCGA cancers and other public cohorts receiving ICT. Therefore, there is an unmet need to develop effective therapies for this patient population that accounts for 13% of patients with cancer. With the available datasets, we identified multiple potential druggable targets (Fig. 6g) including TGF-β signaling^6,42,43^, *CDK6, PRMT1*^49–51^, *CD73*, glucose transporter 1^55,56^, *Siglec-15*^44,45^, *TIGIT* pathway*, VEGFA*^48^, pyruvate kinase M2 (*PKM2*)^52–54^, and *B7-H4* (*VTCN1*) that were upregulated in multiple cancer types, and *CEACAM1*, *IDO1*, and *SIRPa* that were tumor-type specific. Further preclinical and functional studies are warranted to assess their therapeutic potential and build rationale for developing effective combination therapies. These results also highlight the heterogeneous nature of 9p21-loss tumors which necessitate tailored therapy.

Although this study is focused on 9p21 loss, we note that across multiple cancer cohorts, hemizygous deletion (9p21 LOH) is also associated with significantly shorter survival, reduced T-cell abundance and TCR repertoire diversity, lower abundance of T, B, CD8 T cells, cytotoxic lymphocytes, and lower rate of PD-L1 positivity, but with less magnitude compared to the corresponding levels observed in the 9p21-loss tumors. 9p21 LOH may also influence patient response to ICT, as indicated in the mUC cohort showing that the rates of CR/PR diminished significantly with decreasing *MTAP/CDKN2A* expression. A recent clinical trial investigating nivolumab in advanced clear cell renal cell carcinoma (ccRCC) demonstrated that 9p21 deletion (LOH) (*n* = 57) was associated with worse outcomes with PD-1 blockade, however 9p21 LOH was enriched in the infiltrated tumors^18^. Homozygous deletion of 9p21 was not observed in this cohort (0%) and rarely seen in the TCGA (2.9%) ccRCC cohorts, but 9p21 LOH occurs frequently in both cohorts, with a frequency of 25.6% and 26.7%, respectively. However, given that 9p21 LOH did not lead to massive changes in *MTAP/CDKN2A* expression but conferred significantly shorter overall survival, we further conducted a systematic screening of genes, including both coding and non-coding ones, located at the 9p21.3 locus (*n* = 31, Supplementary Data 21) to identify targets for the phenotypic correlates. Our integrative analysis showed that among these 31 genes, *CDKN2A* and *MTAP* were the only two genes displaying significant correlation with tumor-immune phenotypes, patient responses to ICT, and patient survival (Supplementary Fig. 22). We therefore speculate that phenotypic changes observed in 9p21-LOH tumors could be partially due to the haploinsufficiency of *CDKN2A*, as described in a previous study^67^. Given the fact that 9p21 LOH is generally an arm-level event which is different from the focal 9p21 loss, we also acknowledge that other genes located elsewhere on chromosome 9p may have some functional relevance, e.g. *CD274* (encoding the PD-L1) at 9p24.1, which may be co-lost along with 9p21 in some patients. Nevertheless, further investigation will be needed to elucidate the detailed mechanisms. It is noteworthy that most of the potential druggable targets identified in 9p21-loss tumors were also significantly increased in 9p21-LOH tumors (though less magnitude) in comparison with 9p21-WT tumors (Supplementary Fig. 23). Across TCGA cancer studies, tumors with 9p21 LOH account for 25% of patients with cancer, which highlights a broader population of cancer patients who may potentially benefit from 9p21-directed risk stratification and tailored therapies.

Finally, it is important to note that this study was focused on characterizing the molecular consequences and phenotypic correlates of a frequent SCNA event, 9p21-loss, in human cancer, and it was not designed to screen for the best SCNA event correlating with ICT therapy success. There is no doubt that comparing 9p21-loss with other frequent SCNA events can help better define its clinical significance, however in this study, such analysis was limited due to the availability of the genomic datasets. In the mUC cohort from Mariathasan et al., we were able to compare 9p21 loss with other known factors associated with ICT therapy response reported by a recent study^68^. As expected, the levels of TMB, Clonal TMB, APOBEC and UV signatures, *CD8A*, *CXCL9* and *CXCL13* expression were associated with superior response, whereas the presence of 9p21 loss, especially downregulation of *CDKN2A*/*MTAP* expression, was the most significant marker associated with inferior response (Supplementary Fig. 24).

In summary, our data demonstrate that 9p21 loss is a pan-cancer genomic determinant of the cold immune phenotypes and contributes to primary resistance to ICT. 9p21 loss can serve as a potential biomarker of inferior response to ICT and guide patient stratification for therapy and the development of alternative therapeutic interventions.

## Methods

### Patient cohorts, clinical characteristics, sample collection, and filtering

#### MDA metastatic urothelial cancer (mUC) cohort (*n* = 80)

Consecutive patients from MD Anderson Cancer Center who were treated with atezolizumab or pembrolizumab as monotherapy between December 2016 and July 2019 were included in this retrospective analysis. All patients signed an informed consent for use of clinical data for research purposes. This study was approved by the Internal Review Board of MD Anderson Cancer Center. Patient eligibility criteria included histologically confirmed urothelial carcinoma, presence of metastatic disease, treatment with at least one dose of atezolizumab or pembrolizumab, and with available clinical and imaging data prior to initiation of atezolizumab or pembrolizumab. Patients enrolled in any clinical trial investigating atezolizumab or pembrolizumab during the study period were excluded. Initially, 86 mUC patients were identified, 6 of them who had no available PET/CT images for response evaluation were excluded. Finally, a total of 80 mUC patients were identified and included in this study, including 22 patients who received Atezolizumab monotherapy and 58 patients who received pembrolizumab monotherapy. 9p21 status was determined through a CLIA-certified immunohistochemistry (IHC) test of MTAP positivity by IHC staining. An experienced nuclear medicine radiologist (Y.L), blinded to genomic and clinical data, performed tumor measurements using Response Evaluation Criteria in Solid Tumors version 1.1 (RECIST 1.1). Disease-specific survival (DSS) was calculated from the date of first diagnosis of metastasis until recoded death from UC. Progression-free survival (PFS) was calculated from the time of first subsequent immunotherapy dose infusion to the date of radiological progression or death, whichever occurred first. Clinicopathological characteristics of the patients are summarized in the Supplementary Data 12.

#### MDA solid tumor cohort (*n* = 94)

To determine the impact of 9p21 loss on clinical outcomes in patients treated with immune checkpoint inhibitors, we screened the clinical trial database of Institute for Personalized Cancer Therapy (IPCT) at MD Anderson and identified 561 patients with 9p21 loss. 9p21 copy number status was determined based on the copy number profiles inferred from the targeted FoundatioOne CDx panel (through standard bioinformatics pipeline) and/or MTAP protein expression indicated by MTAP immunohistochemistry staining. Pre-treatment tumors with homozygous deletion of 9p21 (i.e. CDKN2A/B homozygous deletion) and/or loss of MTAP protein expression were classified as 9p21-loss and tumor with diploid 9p21 and MTAP stain positive were classified as 9p21-WT. Among 561 patients with 9p21 loss, 71 received anti-PD-1/PD-L1 monotherapy and 48 of them had response/follow-up data available for review. Using the same database, we tried to match (largely but not completely) the cancer type, gender, age, therapy received, and lines of therapy of patients included in the 9p21-loss group, and identified a group of patients (*n* = 52) who were treated with anti-PD-1/PD-L1 monotherapy and whose pre-treatment tumors were 9p21-WT as the control. Six patients were filtered out from the 9p21-loss group due to rare cancer types, leading to a cohort of 94 patients (9p21-Loss = 42, 9p21-WT = 52). The diagnosis of the disease was verified independently by experienced pathologists and the response was confirmed by an experienced radiologist by reading the PET/CT images following the Response Evaluation Criteria in Solid Tumors (RECIST) version 1.1 guideline. A detailed summary of 94 patients was provided in the Supplementary Data 11.

### Public datasets, data processing, sample selection and filtering

#### TCGA datasets

The DNA copy number and bulk mRNA-seq expression data (normalized) generated by The Cancer Genome Atlas (TCGA) Program on 33 tumor types were downloaded from the NCI Cancer Genomic Data Commons (NCI-GDC: https://gdc.cancer.gov). The mRNA-seq expression data were processed and normalized by the NCI-GDC bioinformatics team using their transcriptome analysis pipeline. The clinical annotation of TCGA patients were downloaded from recent TCGA Pan-cancer studies^69,70^. The patients whose survival data were not available (*n* = 152) were excluded from survival analysis. The copy number status of *CDKN2A, MTAP*, and Interferon genes was determined based on the gene-level copy number calls (downloaded from NCI-GDC) inferred by the GISTIC algorithm^71^. The copy number status at chromosomal region 9p21 was carefully investigated and based on which, samples were classified into different groups. Briefly, the tumors with wildtype and diploidy 9p21 were classified into the “9p21-WT” group, which was used as control for subsequent analysis. Tumors that had LOH (loss of heterozygosity) at both CDKN2A and MTAP loci were classified into the “9p21-LOH” group, and tumors had homozygous deletion (HD) of either CDKN2A or MTAP were classified into the “9p21-Loss” group. The copy number status of interferon-alpha family genes was also evaluated and based on which, the samples were further classified into subgroups. The Supplementary Data 1 provides a full list of TCGA samples and their corresponding cancer types and 9p21 status included in this study. The spatial organization of tumor-infiltrating lymphocytes (TILs) and TIL map structure patterns for *n* = 4337 tumors from 13 tumor types were downloaded from Saltz et al.^23^. The Supplementary Data 4 provides a full list of these tumors with 9p21 status.

#### Public datasets of anti-PD-1/L1 clinical trials

A total of six additional public datasets were downloaded from published studies (Table 1). *The MSK advanced NSCLC cohort (n* *=* *151)*: The genomic, PD-L1 expression, and clinical data of *n* = 240 non-small-cell lung cancer (NSCLC) patients were downloaded from Rizvi et al.^17^. Among 240 patients, 186 were lung adenocarcinoma (LUAD) and 27 of them received combinational therapy. The patients with lung adenocarcinoma (LUAD), received anti-PD-1/L1 monotherapy and with response data available (*n* = 151) were then selected for subsequent analysis (Supplementary Data 13). The 9p21 copy number status was determined using the GISTIC copy number calls downloaded from cBioPortal. Somatic mutations identified by the targeted MSK-IMPACT panel^41^ were carefully reviewed and two tumors with truncating mutations in CDKN2A were further excluded. PD-L1 protein expression score (by IHC staining) was available for 55 tumors, of whom 22 had ≥1% expression. For tumor mutation burden (TMB) analysis, tumors with TMB greater than the group median were categorized into “TMB-high” group and that with TMB less than the group median were categorized into “TMB-low” group. The efficacy was assessed by RECIST 1.1 and durable clinical benefit (DCB) was defined by the original study as partial response/stable disease that lasted >6 months^17^. A detailed summary of patients and corresponding immunogenomic features was provided in the Supplementary Data 13.

*The mUC cohort from IMvigor210 trial (n* *=* *298)*: The clinical, bulk RNA-seq, and immune profiling data including PD-L1 protein expression in tumor and immune cells and tumor-immune phenotypes were downloaded from Mariathasan et al.^6^ by following the link (http://research-pub.gene.com/IMvigor210CoreBiologies). The genomic data was not available and 9p21 status was inferred based on the transcriptional expression levels of both *CDKN2A* and *MTAP*. The tumors with high (above group median) expression of both *CDKN2A* and *MTA*P (hi_hi), and that with low (below group median) expression of both *CDKN2A* and *MTA*P (lo_lo) were taken into subsequent analysis. PD-L1 protein expression in tumor and immune cells (by SP142 IHC staining) was available for 297 out of 298 tumors, of whom 102 had ≥5% expression and 112 had ≥1% expression in immune cells, and 42 had ≥5% expression and 17 had ≥1% expression in tumor cells. The immune phenotype data defined by CD8 IHC was available for 244 out of 298 patients. The TMB data was available for 234 patients. A detailed summary of patients and corresponding immunogenomic features was provided in the Supplementary Data 14. To demonstrate the translational relevance of 9p21 loss in the mUC cohort, we built a response score incorporating all 3 factors (9p21, TMB, PD-L1), where a subject gets 2 points for high TMB (because the regression coefficient for TMB is twice the magnitude of the coefficients for the other factors), 1 point for high expression of *CDKN2A* and *MTAP* (hi_hi), and 1 point for high immune cell PD-L1 expression (≥5%, IC2+).

*The metastatic melanoma cohort (n* *=* *58) from Liu* et al.: The clinical and bulk RNA-seq data were downloaded from Liu et al.^37^. Among 144 patients, 60 received ipilimumab before anti-PD-1 treatment and 84 were ipilimumab-naïve. Patients (*n* = 2) with mixed response and tumors (*n* = 7) with ultra-high mutation burden (>1500 nonsynonymous mutations) were excluded. The 58 ipilimumab-naïve tumor specimens collected prior to pembrolizumab or nivolumab monotherapy were then selected. The 9p21 status was inferred based on the transcriptional expression levels of both *CDKN2A* and *MTAP*: tumors with expression levels of both *CDKN2A* and *MTAP* below the group median were classified as “lo_lo”, and tumors with expression levels of both genes above the group median were classified as “hi_hi”. The best overall response rate (per RECIST 1.1 criteria) were compared between the lo_lo and hi_hi groups.

*The metastatic melanoma cohort (n* *=* *41) from Gide* et al.: The clinical data was downloaded from Gide et al.^38^, and the FASTQ files were downloaded from EBI (URL: https://www.ebi.ac.uk/ena/browser/home, accession number PRJEB23709). STAR 2-pass alignment (v2.7.2b)^72^ was performed with default parameters to generate RNA-seq BAM files. Gene-level expression quantification was performed using HTSeq-count (v0.11.0)^73^. The raw read counts generated from HTSeq-count were normalized into fragments per kilobase of transcript per million mapped reads (FPKM) using the RNA-seq quantification approach suggested by the bioinformatics team of NCI Genomic Data Commons (GDC; https://gdc.cancer.gov/about-data/data-harmonization-andgeneration/genomic-data-harmonization/high-level-data-generation/rna-seq-quantification). Among 120 patients, 63 were treated with anti-PD-1 (pembrolizumab or nivolumab) monotherapy and 13 out of 63 were excluded due to lack of RNA-seq data. Among these 50 patients, 9 cases with pre-treatment tumor samples unavailable were further excluded, resulting 41 patients for subsequent analyses. The 9p21 status was inferred based on the transcriptional expression levels of both *CDKN2A* and *MTAP*: tumors with expression levels of both *CDKN2A* and *MTAP* below the group median were classified as “lo_lo”, and tumors with expression levels of both genes above the group median were classified as “hi_hi”.

*The high-risk resectable melanoma cohort (n* *=* *12)*: the clinical, response, and RNA-seq were downloaded from our recent studies^27,74^. The 12 baseline samples prior to nivolumab monotherapy were selected. Similarly, as described above, the 9p21 status was inferred based on the transcriptional expression levels of both *CDKN2A* and *MTAP*. The response was assessed by RECIST 1.1 criteria^74^.

*The unresectable or advanced melanoma cohort (n* *=* *23) from Riaz* et al.: the clinical, response, and RNA-seq of this unresectable/advanced melanoma cohort were downloaded from Riaz et al.^39^. (GSE91061). Among 109 samples (51 pre-treatment and 58 on-treatment) from 65 patients, the 51 pre-nivolumab biopsies (from 51 patients) were selected. Patients progressed on ipilimumab prior to nivolumab therapy (*n* = 26) and those lack of response data (*n* = 2) were further excluded, resulting a cohort of 23 patients from downstream analyses. The 9p21 status was inferred based on the transcriptional expression levels of both *CDKN2A* and *MTAP* as described above. Tumor response for patients was defined by RECIST v1.1 by the original study.

### Analysis of bulk RNA-seq data

#### Immune deconvolution

The R package MCP-counter^24^ was applied to the normalized log2-transformed expression matrix to infer the absolute abundance scores for eight major immune cell types (B lineage, T cells, CD8 T cells, cytotoxic lymphocytes, NK cells, monocytic lineage, myeloid dendritic cells, and neutrophils), endothelial cells, and fibroblasts. In addition, another computational approach CIBERSORT^25^ was applied to the normalized RNA-seq data to estimate the relative proportions of 22 immune cell subpopulations using compartment-specific gene expression signatures. For TCGA cohorts, the CIBERSORT deconvolution results, TCR richness and Shannon entropy derived from bulk RNA-seq data, and the leukocyte fraction inferred from DNA methylation signatures were downloaded from a recent TCGA PanCanAtlas study^69^. The deconvolution results from MCP-counter and CIBERSORT were compared between the 9p21-loss and 9p21-WT groups using Wilcoxon rank-sum test. We applied the Benjamini–Hochberg method to correct the *P*-values and the false discovery rate (FDR q-values) were calculated.

#### Differential gene expression and pathway enrichment analysis

Wilcoxon rank-sum test was used to identify differentially expressed genes between the 9p21-loss and 9p21-WT groups. A cutoff gene expression fold change of ≥1.2 or ≤−1.2 and a FDR *q*-value of <0.05 was applied to select the most significant DEGs. For pathway analysis, the curated gene sets of 41 immune signaling pathways (from the Biocarta, Hallmark, KEGG, PID, Reactome databases) (Supplementary Data 7) were downloaded from the Molecular Signature Database (MSigDB: http://software.broadinstitute.org/gsea/msigdb/index.jsp). Single-sample gene set enrichment analysis (ssGSEA) was applied and pathway scores were calculated for each sample using *the* GSVA software package^75^. The pathway scores were then compared between the 9p21-loss and 9p21-WT groups. Pathway enrichment analysis was done with the limma R software package. A cutoff fold change of ≥1.2 or ≤−1.2 and a FDR q-value of < 0.05 was applied to select the most significantly enriched signaling pathways.

### Survival analysis

For survival analysis, including overall survival (OS), progression-free survival (PFS), and disease-specific survival (DSS), we used the log-rank test to calculate *P*-values between the stratified patient groups (e.g. 9p21-loss, 9p21-LOH, 9p21-WT, hi_hi, lo_lo) and the Kaplan-Meier method to plot survival curves. The numbers at risk, median survival times or times since treatment were calculated for each group. The survival data of TCGA patients were downloaded from a recent TCGA Pan-cancer study^70^. For other public datasets, the survival data were downloaded from their corresponding published studies. The patients whose survival data were not available were excluded from survival analysis. Cox proportional hazards (PH) regression model was used to calculate the Hazard Ratio (HR), the 95% confidence interval (95%CI), and *P* values.

### Statistical analysis

In addition to the bioinformatics approaches described above, Fisher’s Exact test was applied to determine the proportion differences between groups, and Spearman’s correlation analysis was used to identify genes significantly correlated with *CDKN2A/MTAP* expression. The logistic regression model was used to calculate the correlation between the potential biomarkers and patient response. All other statistical analyses were performed using statistical software R v3.4.3. JMP Pro (v14) was used for data visualization and illustration. To control for multiple hypothesis testing, we applied the Benjamini-Hochberg method to correct *P*-values and the false discovery rates (*q-*values) were calculated. All statistical significance testing in this study was two-sided and results were considered statistically significant at *P*-values or FDR *q-*values < 0.05. When a *P* value reported by R (v3.4.3) was smaller than 2e-16, it was reported as “*P* < 2 × 10^−16^”.

### Reporting summary

Further information on research design is available in the Nature Research Reporting Summary linked to this article.

## Supplementary information

Supplementary Information

Description of Additional Supplementary Files

Supplementary Data 1-21

Reporting Summary

## Data Availability

For TCGA cohorts, the genomic and clinical data can be retrieved from NCI Genomic Data Commons (NCI-GDC: https://gdc.cancer.gov). For the melanoma cohorts from Helmink et al. (GSE120575) and Riaz et al. (GSE91061), the data can be obtained from the Gene Expression Omnibus (GEO) database [https://www.ncbi.nlm.nih.gov/geo/]. Data of the Urothelial cancer cohort from Mariathasan et al. (mUC IMvigor210 trial) can be downloaded from http://research-pub.gene.com/IMvigor210CoreBiologies. Data of the MSKCC advanced NSCLC cohort from Rizvi et al. can be obtained from the cBioPortal [https://www.cbioportal.org/]. The clinical response data of MDA solid tumor cohort and MDA metastatic urothelial cancer cohort were shared in Supplementary Data 11 and 12, respectively. The data that support the main findings of this study are provided in Supplementary Data 3, 4, 6, 8,11–16, 18–20. For MDA mUC cohort and MDA solid tumor cohort, the patient related data (sex, age, diagnosis, and date of last follow up) not included in the paper are subjected to patient confidentiality. Further information and requests should be directed to and will be fulfilled by the Lead Contact, Dr. Linghua Wang (LWang22@mdanderson.org). All requests for data and materials will be promptly reviewed by The University of Texas MD Anderson Cancer Center to verify if the request is subject to any intellectual property or confidentiality obligations. Any data and materials that can be shared will be released via a Material Transfer Agreement.
