# Supplementary material for: 9p21 loss confers a cold tumor immune microenvironment and primary resistance to immune checkpoint therapy
Source: Nat Commun. 2021 Sep 23;12:5606. doi: 10.1038/s41467-021-25894-9 (PMC8460828; doi:10.1038/s41467-021-25894-9)
Supplement: Supplementary file 1 — Supplementary Information [file 41467_2021_25894_MOESM1_ESM.pdf]

Supplementary Figure 1

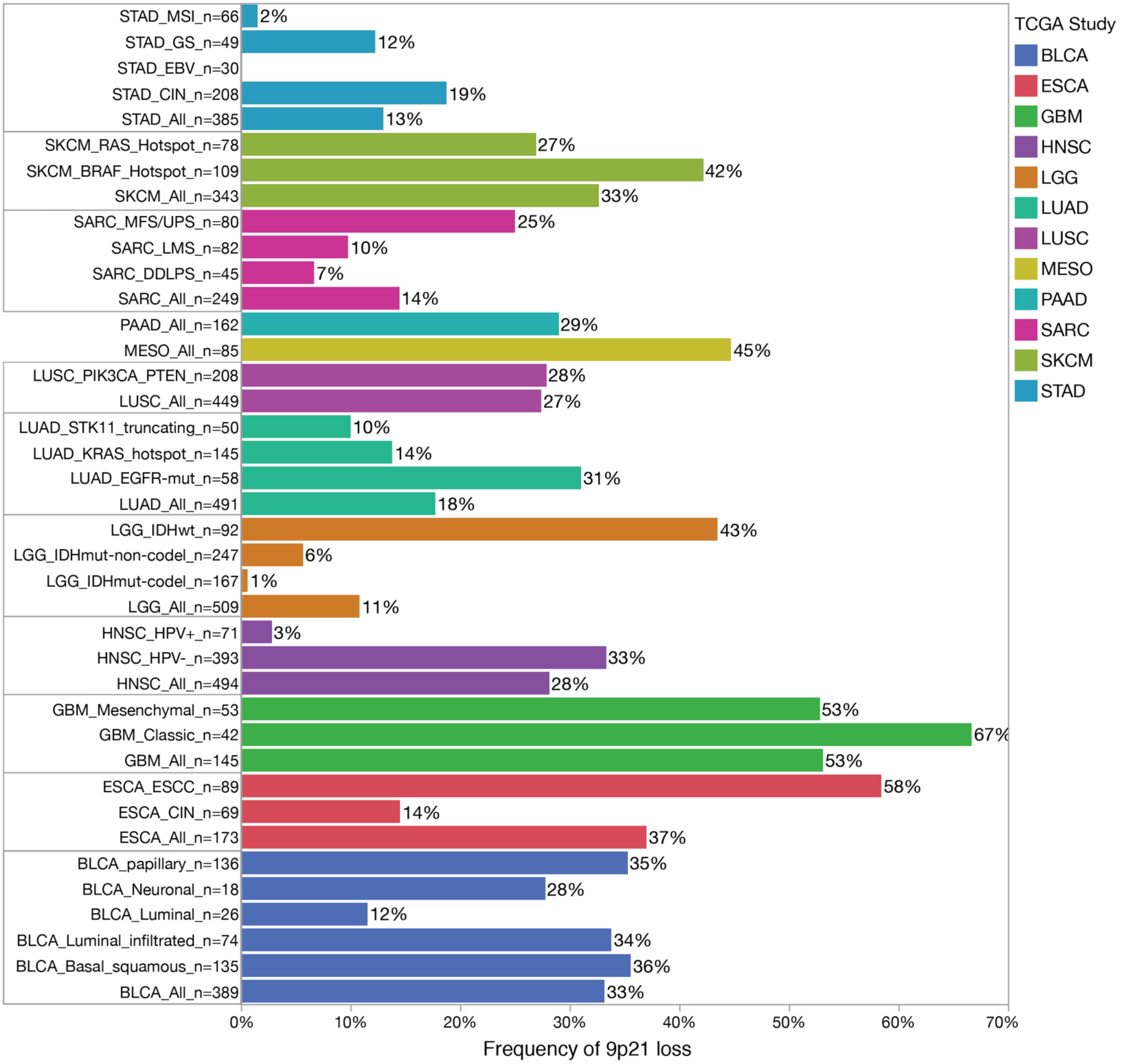

Supplementary Figure 1. Chromosome 9p21 loss is frequently observed in human cancers. The frequency of 9p21 loss varied greatly across TCGA cohorts and their molecular subtypes. The top 12 TCGA cancer types with most frequent 9p21 loss (frequency>10%) were included in the analysis and the frequency of 9p21 loss were compared across their molecular subtypes. The histogram is colored by tumor type and the frequency of 9p21 loss in each cohort (and their molecular subtypes) is labeled on the plot. The TCGA disease codes and other abbreviations were listed in Supplementary Data 2.

Supplementary Figure 2

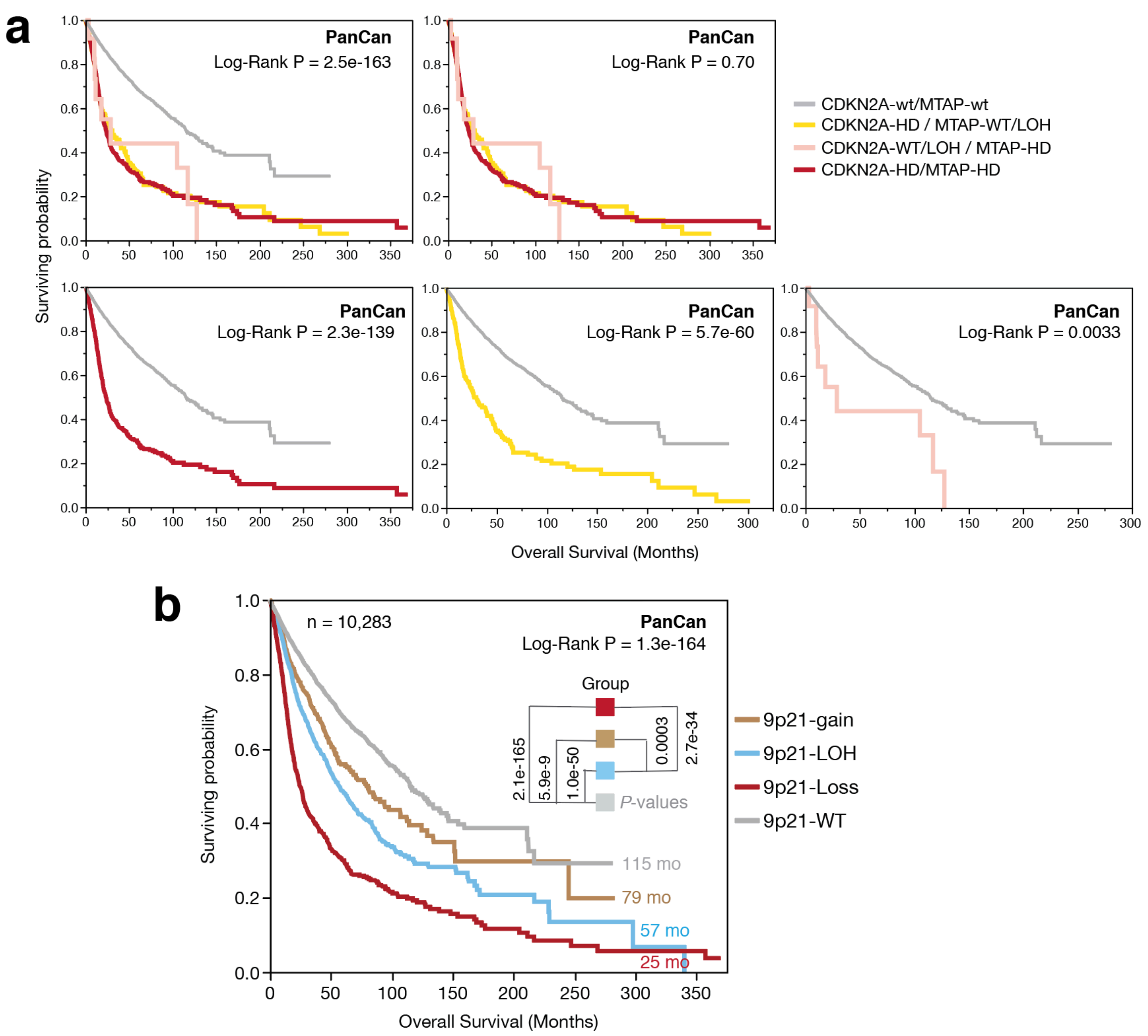

Supplementary Figure 2. Chromosome 9p21 loss is associated with significantly shortened patient survival. In total, 10,283 out of 10,435 patients with available survival data were included in the overall survival analysis. The analysis was stratified by *MTAP/CDKN2A* (**a**) and 9p21 status (**b**), respectively. The patient groups are the same as shown in the main Figure 1g and Supplementary Data 1. The median survival time for each stratified patient group, and the Log-rank P-values were labelled on the plots. PanCan, pan-cancer analysis for TCGA studies. P values were calculated with two-sided Logrank test.

Supplementary Figure 3

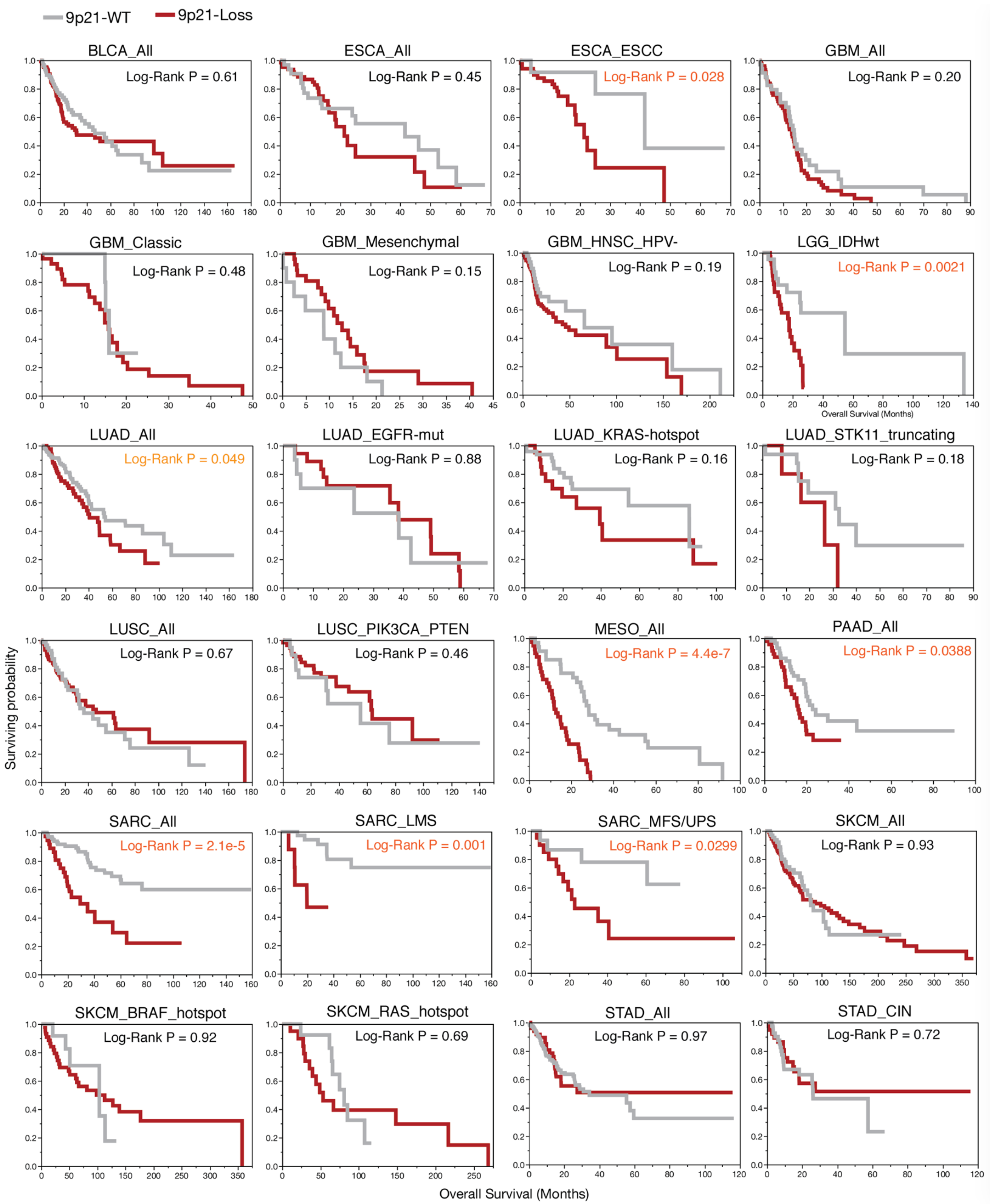

Supplementary Figure 3. Homozygous deletion of 9p21 (9p21-Loss) is associated with worse patient survival in TCGA cohorts. The overall survival analysis was stratified by 9p21 status (Loss vs. WT) across 12 TCGA cohorts with frequent 9p21 loss. The list of 12 cohorts and 14 molecular subtypes and their corresponding sample size are shown in the Supplementary Data 3. P values were calculated with two-sided Logrank test.

Supplementary Figure 4

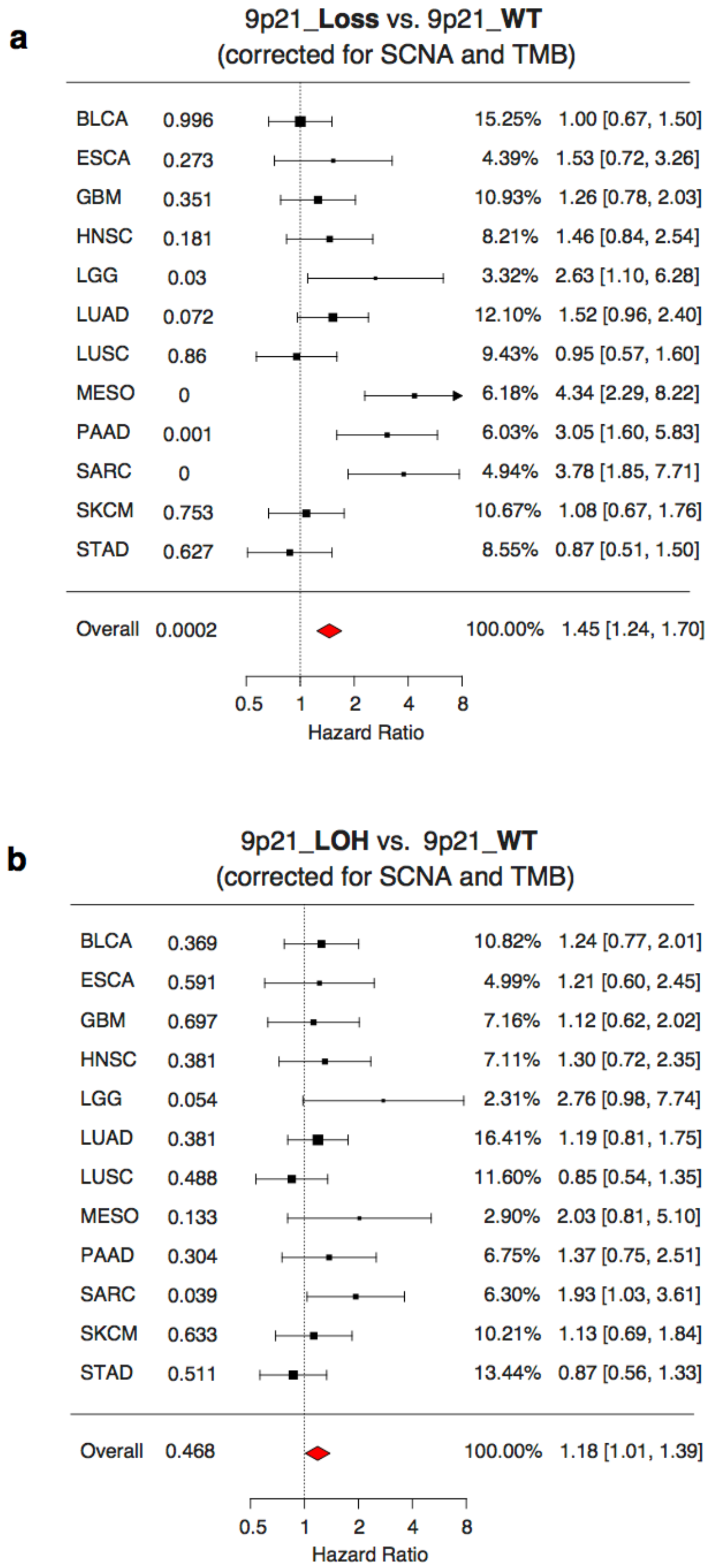

Supplementary Figure 4. Prognostic significance for 9p21 loss and 9p21 LOH across 12 tumor types adjusted for SCNA burden and TMB. **a**, The differences in overall survival (OS) time between 9p21-loss tumors and 9p21-WT tumors. Cohorts sample size: BLCA (231), ESCA (160), GBM (183), HNSC (180), LGG (68), LUAD (372), LUSC (258), MESO (72), PAAD (107), SARC (223), SKCM (278), STAD (305). **b**, The differences in overall survival (OS) time between 9p21-LOH tumors and 9p21-WT tumors. Sample size: BLCA (n = 201), ESCA (n = 118), GBM (n = 98), HNSC (n = 168), LGG (n = 49), LUAD (n = 533), LUSC (n = 401), MESO (n = 46), PAAD (n = 104), SARC (n = 277), SKCM (n = 326), STAD (n = 439). Cohort name, p values, weights and 95%CI estimate of OR were shown in forest plots. P values were calculated and adjusted for SCNA burden, TMB using multivariable Cox regression model.

Supplementary Figure 5

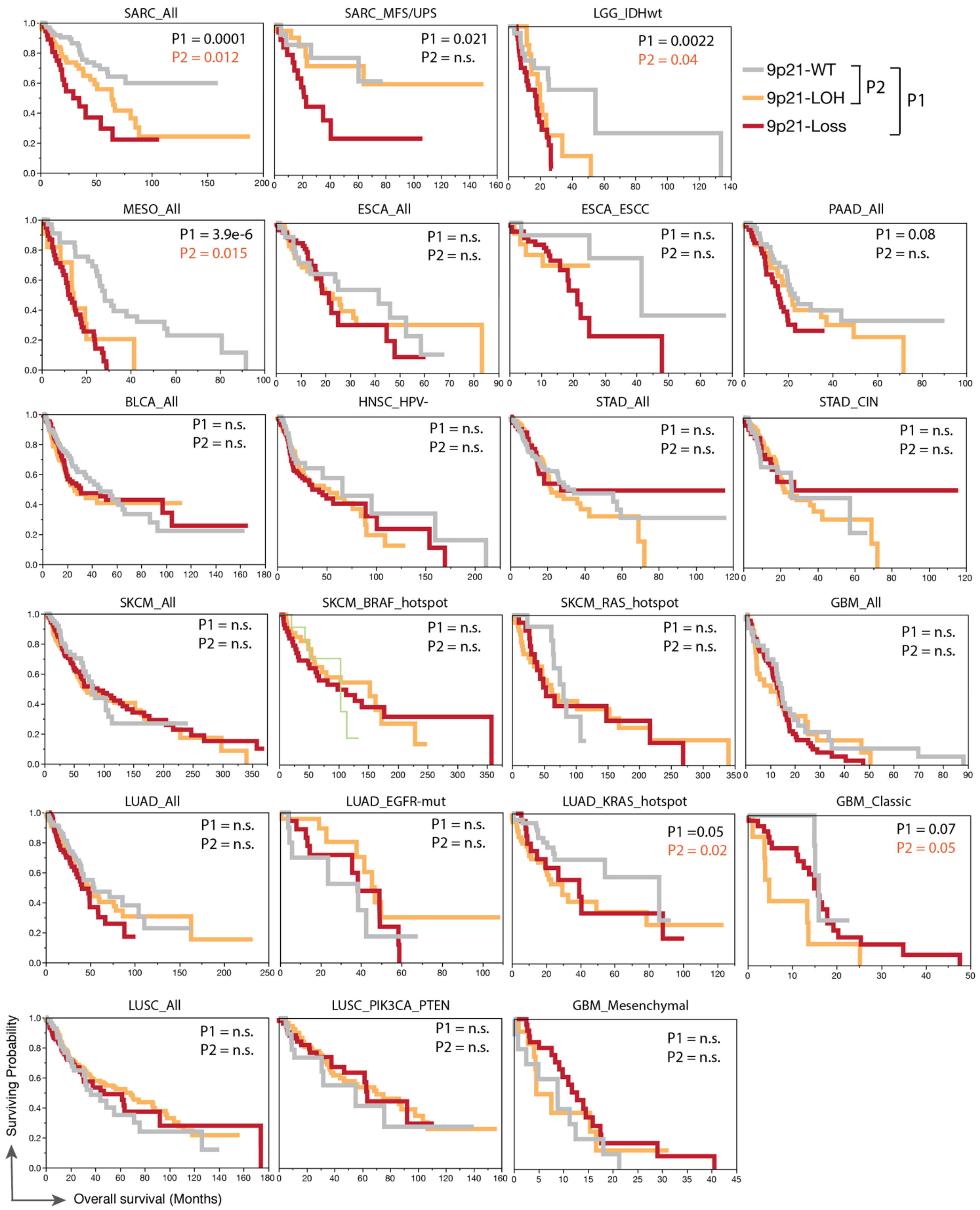

Supplementary Figure 5. Hemizygous deletion of 9p21 (9p21-LOH) is associated with worse patient survival in multiple TCGA cohorts. For each tumor type and molecular subtype, the overall survival time was compared across 3 patient groups: patients who had homozygous deletion of 9p21 *(*9p21-Loss*)*, patients who had hemizygous deletion of 9p21 (9p21-LOH), and patients who had intact and wild-type 9p21 (9p21-WT). The list of 12 TCGA cohorts, 14 molecular subtypes and their corresponding sample size are shown in the Supplementary Data 3. P values for corresponding comparisons were labelled. P values were calculated with two-sided Logrank test.

Supplementary Figure 6

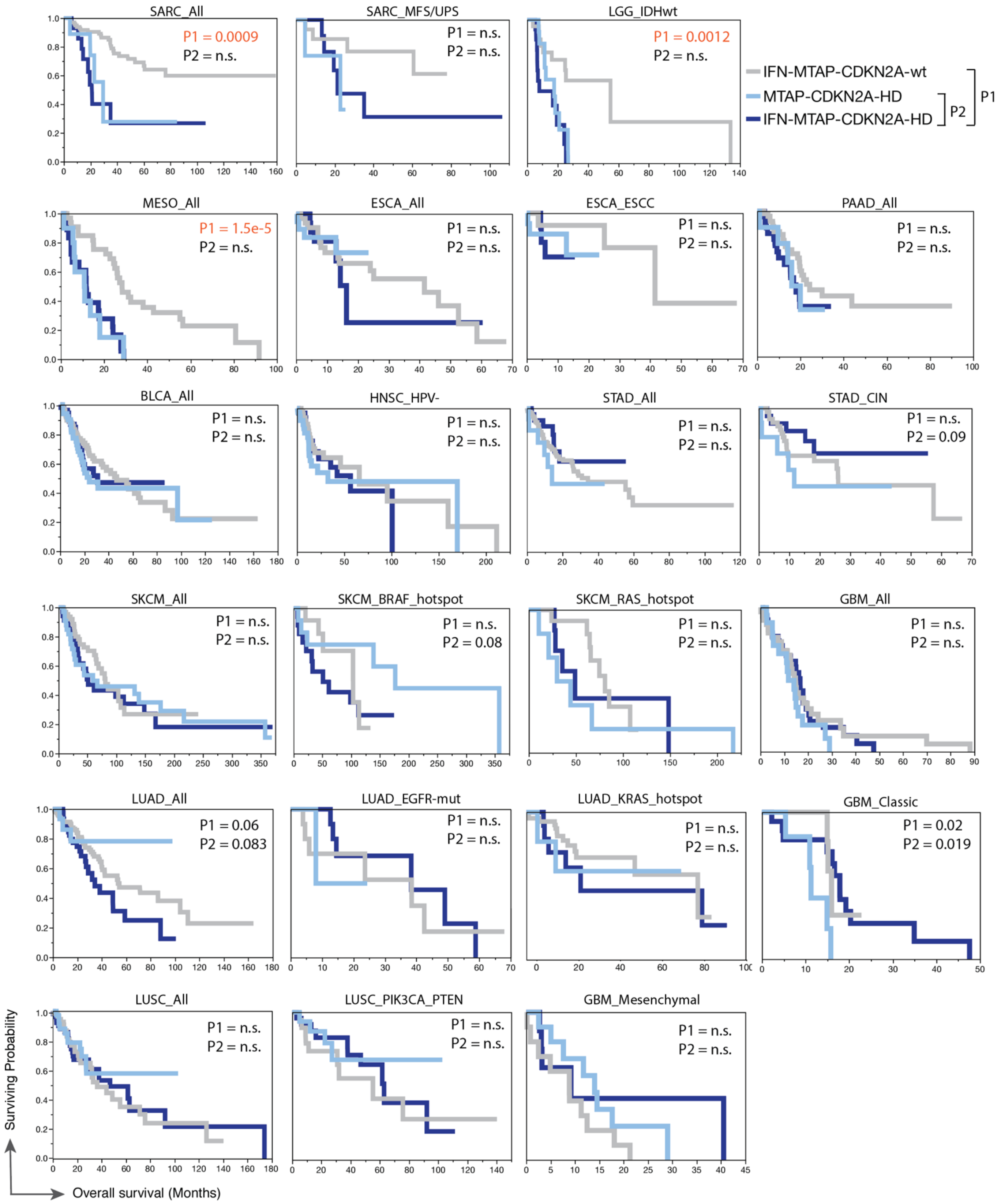

Supplementary Figure 6. Deletion of additional IFN genes showed no difference in patient survival in tumors with co-deletion of *CDKN2A and MTAP*. The overall survival was compared between patients who had tri-deletion of *CDKN2A*/*MTAP*/*IFN* genes (IFN-MTAP-CDKN2A-HD), and those with co-deletion of *CDKN2A*/*MTAP* (MTAP-CDKN2A-HD), and those had intact and wild-type 9p21 (IFN-MTAP-CDKN2A-wt) in their tumors. P values for corresponding comparisons were labelled. P values were calculated with two-sided Logrank test.

Supplementary Figure 7

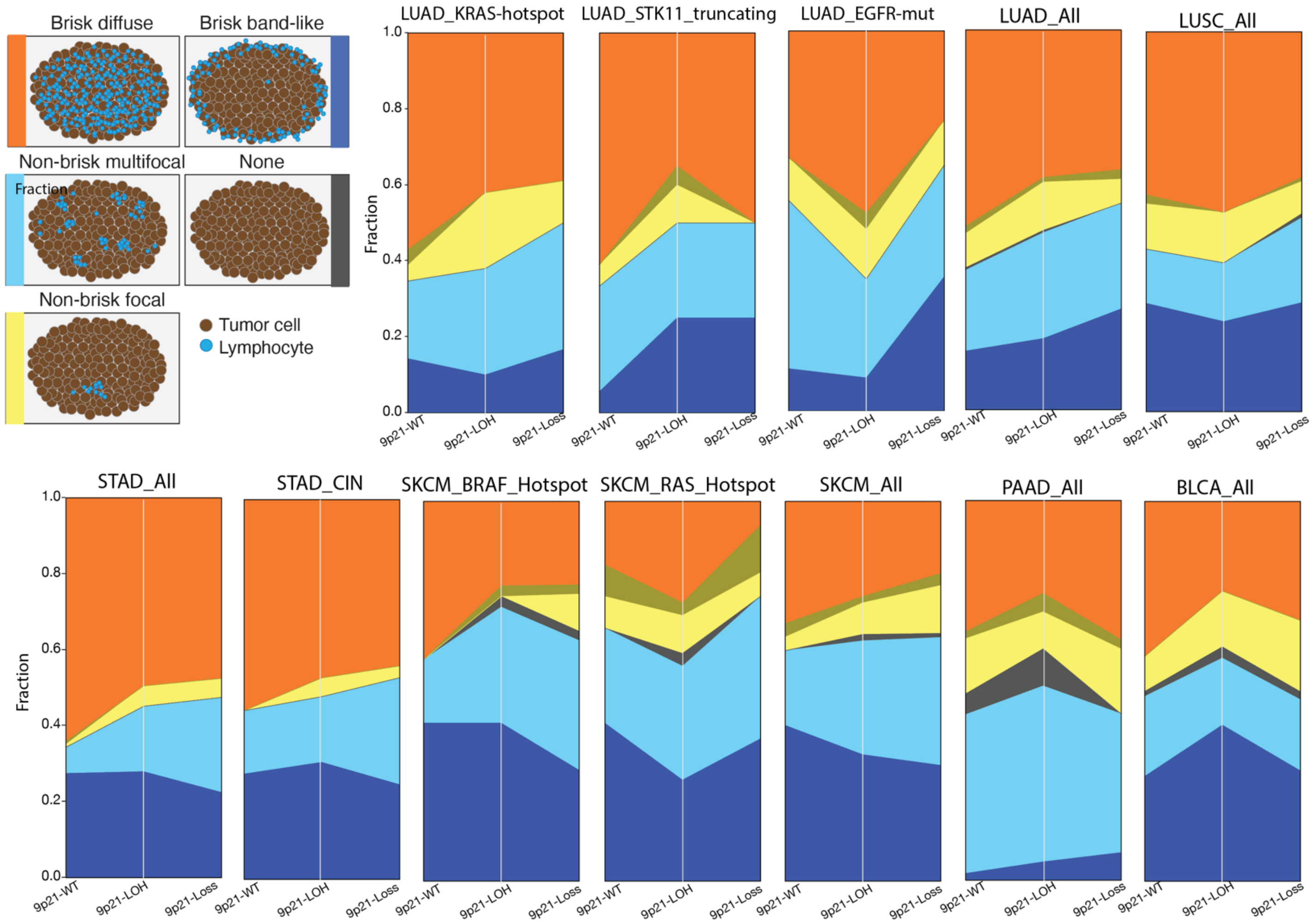

Supplementary Figure 7. The fraction of immunologically 'inflamed' phenotype was decreased, and the "cold" immune phenotypes were increased in tumors with 9p21 loss. The patterns of spatial distribution of tumor-infiltrating lymphocytes (TILs) were compared across 3 groups: tumors with homozygous deletion of 9p21 *(*9p21-Loss*)*, tumors with hemizygous deletion of 9p21 (9p21-LOH), and tumors with intact and wild-type 9p21 (9p21-WT). The TCGA disease codes and other abbreviations were listed in Supplementary Data 2.

Supplementary Figure 8

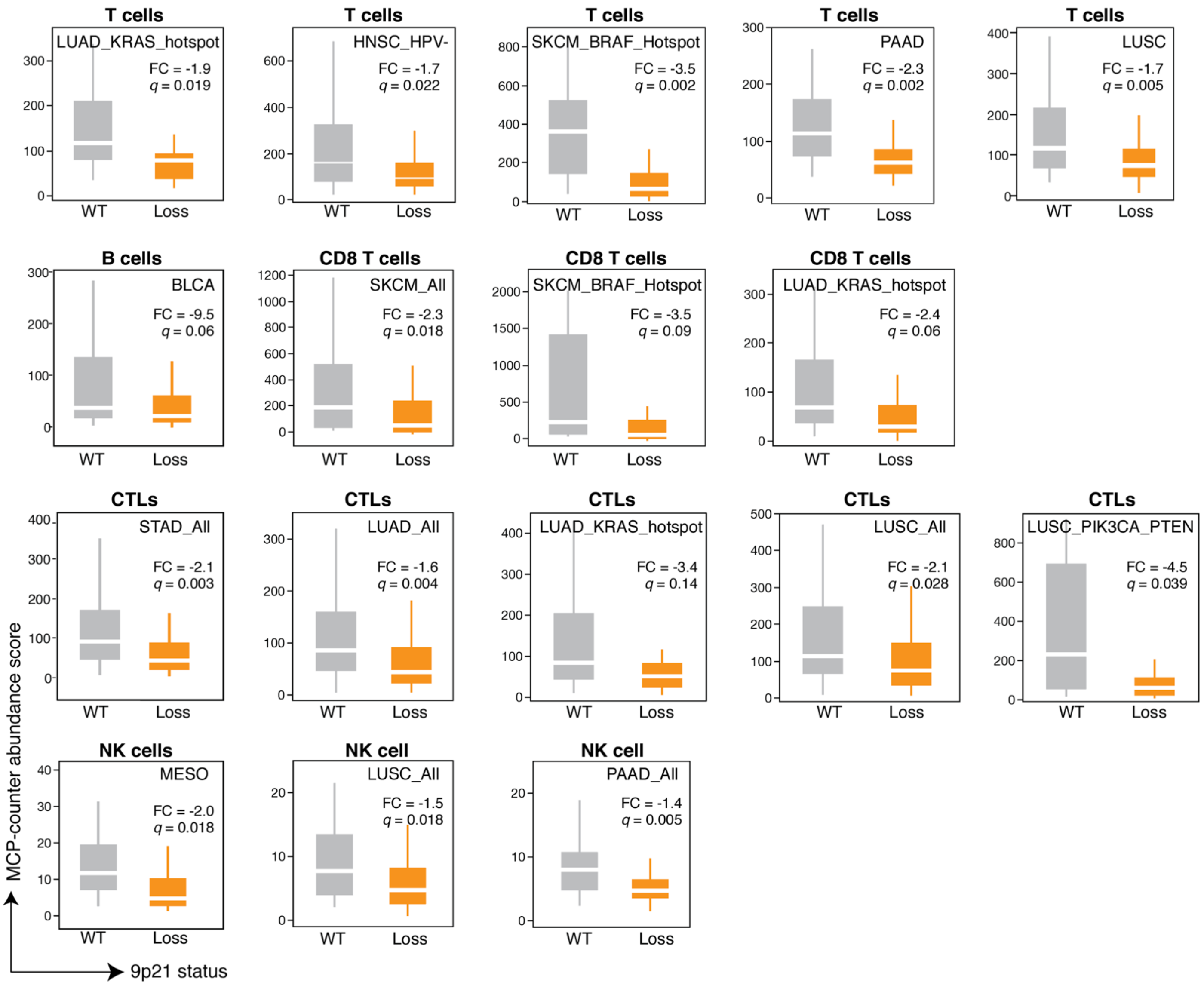

Supplementary Figure 8. The immune cell abundance (B, T, CD8 T, NK, and CTLs) was remarkably decreased in tumors with 9p21 loss. Box plots of representative examples selected from the Fig. 2c are shown. The MCP-counter derived immune cell abundance scores were compared between tumors with 9p21 loss and those with intact and wildtype 9p21. The fold change (FC) in immune cell abundance and adjusted p values (q values) were labeled on each plot. P values were calculated with two-sided Wilcoxon rank-sum tests. P values were adjusted for multiple testing using the Benjamini–Hochberg method. Box, median ± interquartile range; whiskers, 1.5× interquartile range. The TCGA disease codes and other abbreviations were listed in Supplementary Data 2. T cells of LUAD_KRAS_hotspot (WT: n = 47; Loss: n = 19), HNSC_HPV- (WT: n = 49; Loss: n = 131), SKCM_BRAF_hotspot (WT: n = 13; Loss: n = 46), PAAD (WT: n = 42; Loss: n = 44), LUSC (WT: n = 57; Loss: n = 123). B cells of BLCA (WT: n = 97; Loss: n = 127), SKCM (WT: n = 71, Loss: n = 112), SKCM_BRAF_hotspot (WT: n = 13; Loss: n = 46), LUAD_KRAS_hotspot (WT: n = 47, Loss: n = 19). CTLs of STAD (WT: n = 163; Loss: n = 48), LUAD (WT: n = 144; Loss: n = 78), LUAD_KRAS_hotspot (WT: n = 47; Loss: n = 19), LUSC (WT: n = 57; Loss: n = 123), LUSC_PI3KCA_PTEN (WT: n = 20; Loss: n = 58). NK cells of MESO (WT: n = 32; Loss: n = 37), LUSC (WT: n = 57; Loss: n = 123), PAAD (WT: n = 42; Loss: n = 44).

Supplementary Figure 9

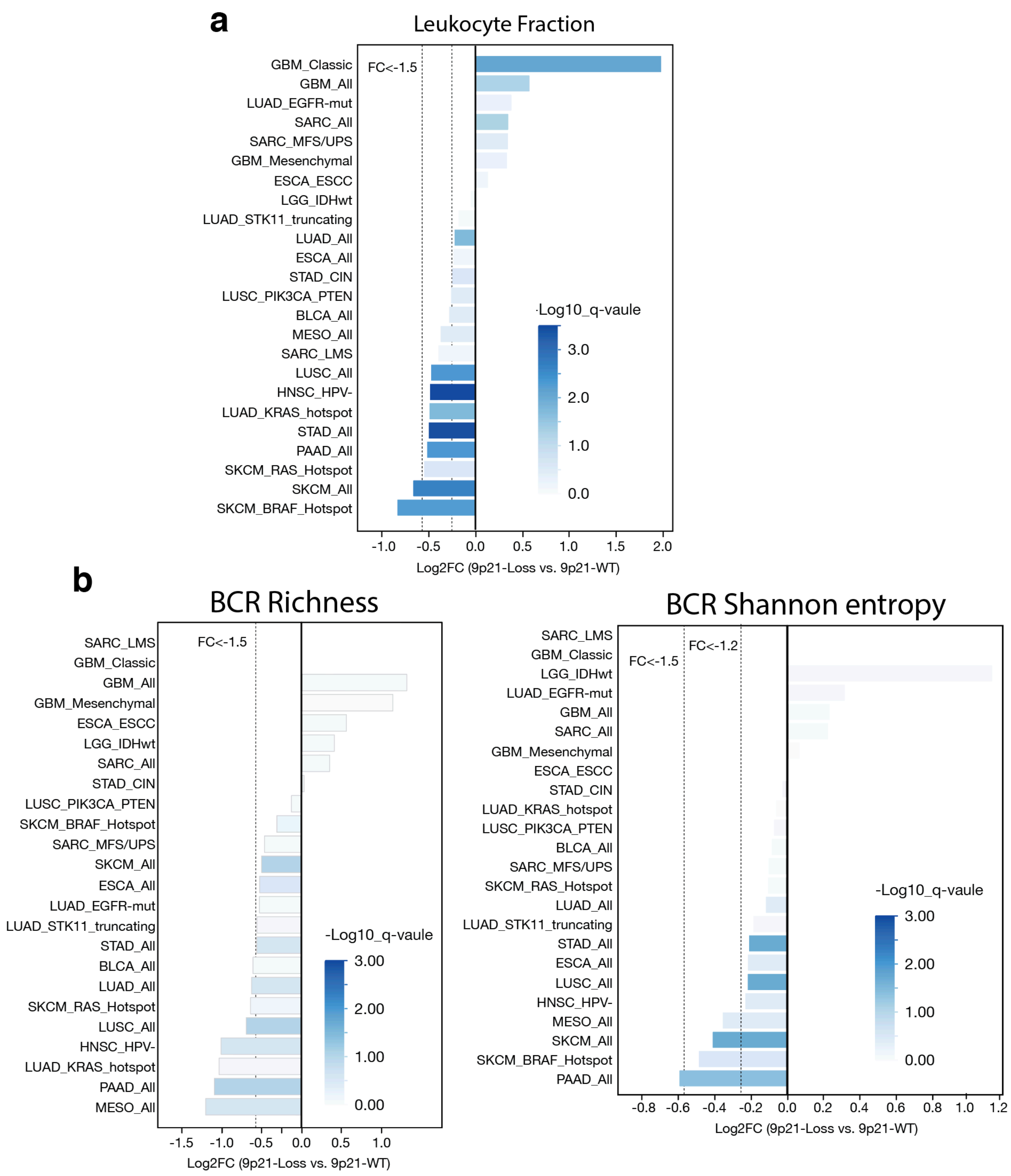

Supplementary Figure 9. 9p21 loss is associated with decreased leukocyte fraction, B cell receptor repertoire richness and clonotype diversity. a-b, The leukocyte fraction (**a**), BCR richness and Shannon entropy (**b**) were computed by TCGA working groups (Immunity, 2019). The scores were compared between tumors with 9p21 loss and tumors that had wildtype 9p21. The vertical dashed lines indicate fold change (9p21 loss vs. 9p21-WT) of <-1.2 and -1.5, respectively. Colors of bars indicate the significance level.

Supplementary Figure 10

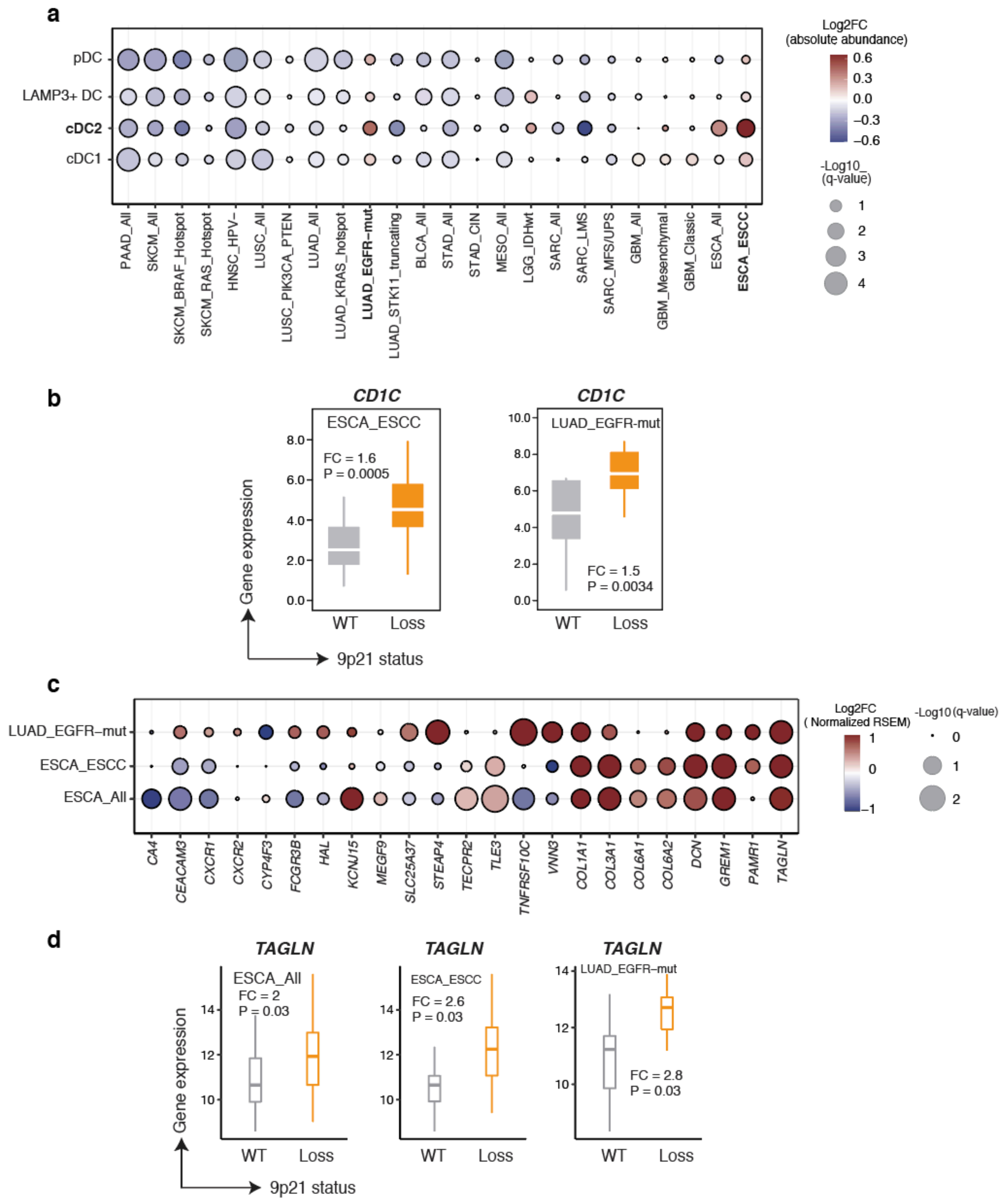

Supplementary Figure 10. 9p21 loss associated changes in the abundance level of myeloid cells (a, b), neutrophils (c) and CAFs (c, d). The bubble plot is drawn using computed abundance level fold change (**a**) and log2-transformed fold change (9p21-Loss vs. 9p21-WT, **c**) and adjusted p-values (FDR q-value). The size of the bubble indicates statistical difference. The colors of bubbles indicate significance level of changes in the myeloid cell subsets abundance (**a**) or marker genes expression level (**c**). The normalized expression (log-transformed TPM) values were compared between tumors with 9p21 loss (9p21-Loss) and tumors with wildtype 9p21 (9p21-WT). The sizes of bubbles indicate the level of changes in absolute abundance (**a**) or expression level (**c**). P values in panels B and D were calculated using two-sided Wilcoxon rank-sum test. Box, median ± interquartile range; whiskers, 1.5× interquartile range. Sample size: **b**: ESCA_ESCC WT (n = 12), ESCA_ESCC (Loss = 52), LUAD_EGFR-mut WT (n = 10), LUAD_EGFR-mut Loss (n = 18). **d**: ESCA WT (n = 32), ESCA Loss (n = 64), ESCA_ESCC WT (n = 12), ESCA_ESCC Loss (n = 52), LUAD_EGFR-mut WT (n = 10), ESCA Loss (n = 18).

Supplementary Figure 11

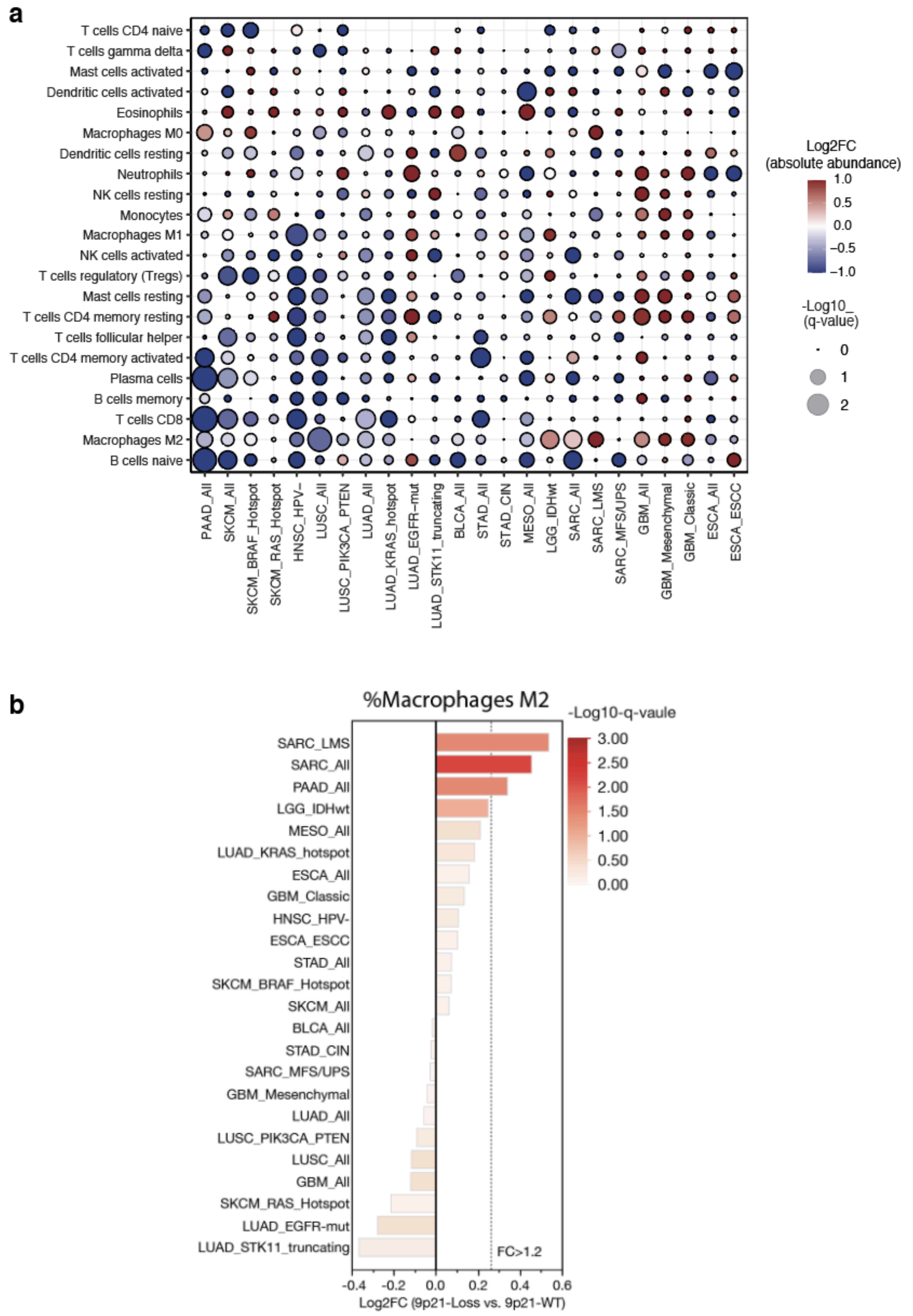

Supplementary Figure 11. Cell component changes associated with 9p21 loss. a, Absolute immune deconvolution level changes in 9p21-loss tumors (vs. 9p21-WT). Immune deconvolution was performed by applying CIBERSORTx to the bulk RNA-seq data. The data is shown for 12 TCGA cohorts (14 molecular subtypes) with frequent 9p21 loss. The size of the bubble indicates statistical significance level of the difference. The color of the bubble indicates change in the immune cell abundance in 9p21-loss tumors (vs. 9p21-WT). **b**, Increased fractions of M2-like macrophages in 9p21-loss tumors of GBM (vs. 9p21-WT). The CIBERSORT deconvoluted immune cell fractions were compared between 9p21-loss tumors and 9p21-WT tumors.

Supplementary Figure 12

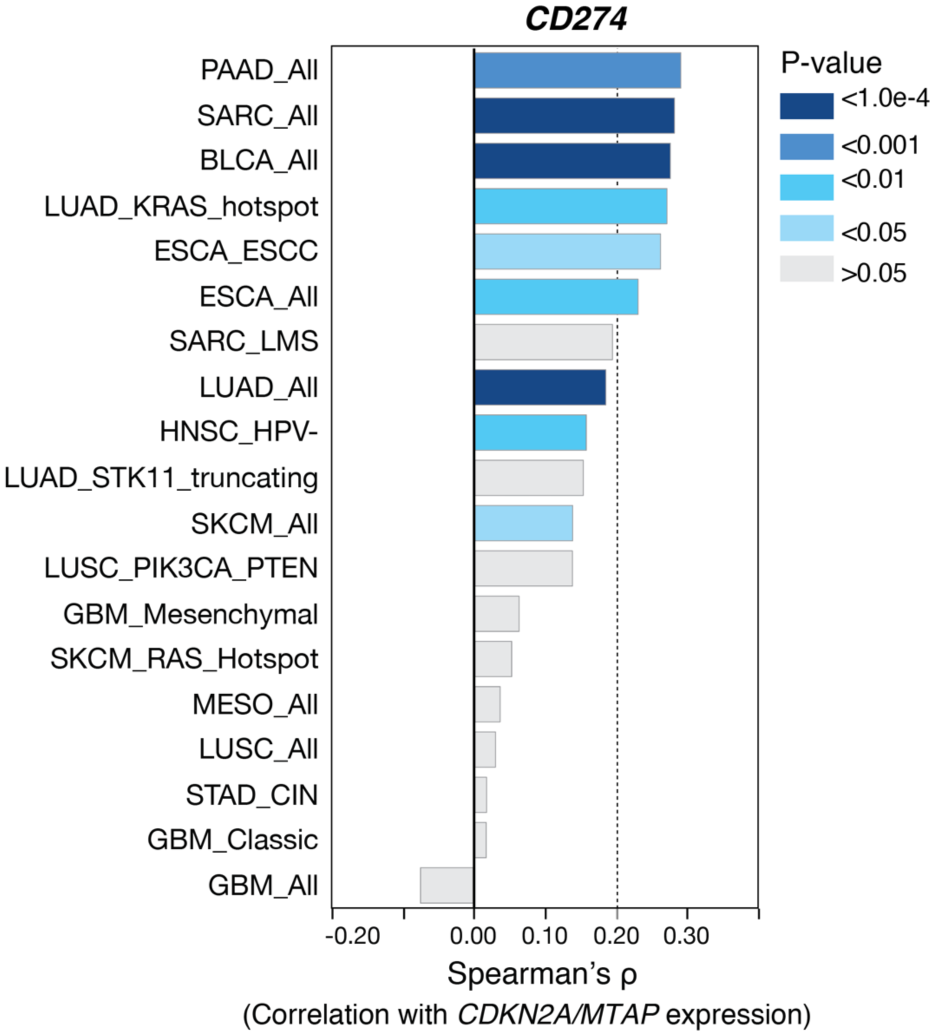

Supplementary Figure 12. The levels of *CD274 (PD-L1)* expression were correlated with that of *CDKN2A/MTAP* expression in TCGA cohorts. The Spearman correlation score is shown on the x axis and the bars are color coded by P-value. Colors of bars indicate the significance level.

Supplementary Figure 13

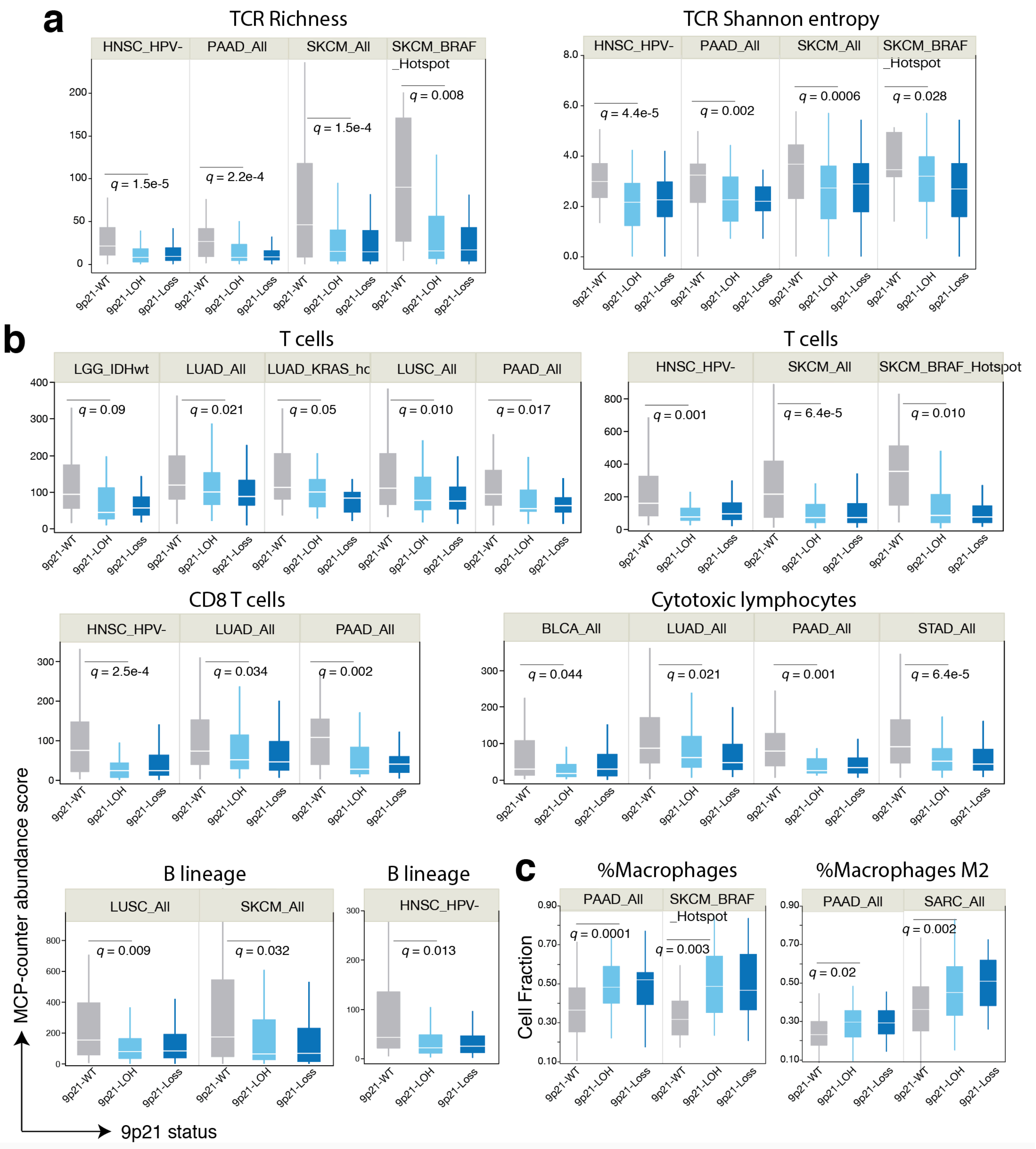

Supplementary Figure 13. Hemizygous deletion of 9p21 (9p21-LOH) is associated with "cold" TME in TCGA cohorts. a-c, For each tumor type and molecular subtype, the richness and diversity of TCR repertoire (**a**), the MCP-counter and CIBERSORT deconvoluted immune cell abundance/fractions (**b-c**) were compared across 3 groups: tumors with homozygous deletion of 9p21 *(*9p21-Loss*)*, tumors with hemizygous deletion of 9p21 (9p21-LOH), and tumors with wild-type 9p21 (9p21-WT). Box, median ± interquartile range; whiskers, 1.5× interquartile range. The list of 12 TCGA cohorts, the molecular subtypes and their corresponding sample size are shown in the Supplementary Data 3. Sample size: A: HNSC_HPV- (WT: n = 49, LOH: n = 119, Loss: n = 131), PAAD (WT: n = 60, LOH: n = 44, Loss: n = 47), SKCM (WT: n = 71, LOH: n = 144, Los: n = 112),

SKCM_BRAF (WT: n = 13, LOH: n = 43, Loss: n = 46). B: LGG_IDHwt (WT: n = 28, LOH: n = 21, Loss: n = 40), LUAD (WT: n = 162, LOH: n = 21, Loss: n = 40), LUAD_KRAS_hotspot (WT: n = 51, LOH: n = 54, Loss: n = 20), LUSC (WT: n = 57, LOH: n = 54, Loss: n = 20), PAAD (WT: n = 60, LOH: n = 44, Loss: n = 47), HNSC_HPV- (WT: n = 49, LOH: n = 119, Loss: n = 131), SKCM (WT: n = 71, LOH: n = 144, Loss: n = 112), SKCM_BRAF_hotspot (WT: n = 13, LOH: n = 43, Loss: n = 112), BLCA (WT: n = 102, LOH: n = 99, Loss: n = 129), STAD (WT: n = 171, LOH: n = 121, Loss: n = 50). C: PAAD (WT: n = 60, LOH: n = 44, Loss: n = 47), SKCM_BRAF (WT: n = 13, LOH: n = 43, Loss: n = 46), SARC (WT: n = 103, LOH: n = 36, Loss: n = 71).

Supplementary Figure 14

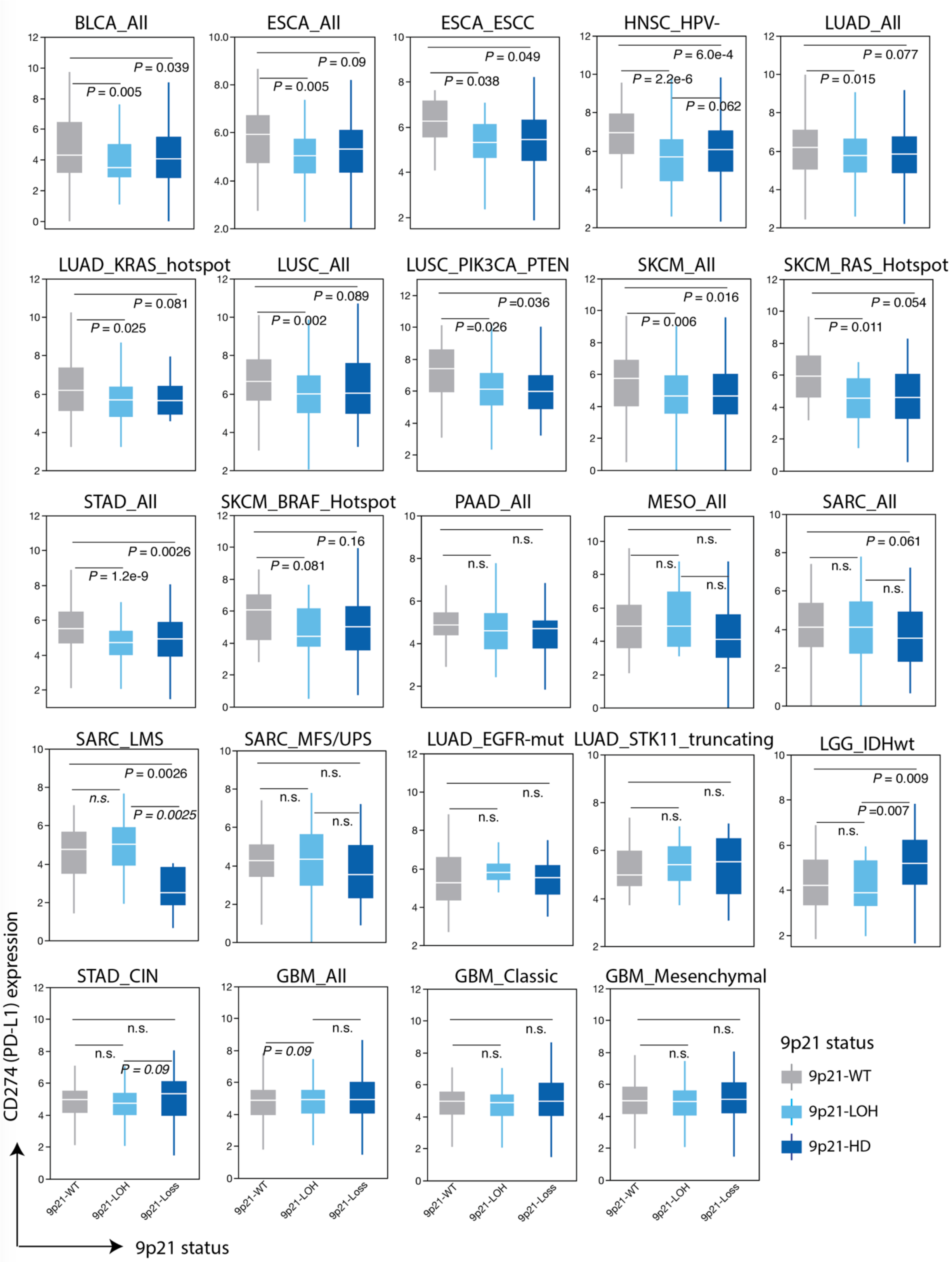

Supplementary Figure 14. Hemizygous deletion of 9p21 (9p21-LOH) is associated with decreased *CD274 (PD-L1)* expression in TCGA cohorts. For each tumor type and molecular subtype, the *CD274* mRNA expression levels were compared across 3 groups: tumors with homozygous deletion of 9p21 (9p21-Loss), tumors with hemizygous deletion of 9p21 (9p21-LOH), and tumors with intact and wild-type 9p21 (9p21-WT). P values were calculated by two-sided Wilcoxon rank-sum test. Box, median ± interquartile range; whiskers, 1.5× interquartile range. Sample size: BLCA (WT: n = 102, LOH: n = 99, Loss: n = 129), ESCA (WT: n = 32, LOH: n

= 59, Loss: n = 64), ESCA_ESCC (WT: n = 12, LOH: n = 15, Loss: n = 52), HNSC_HPV- (WT: n = 49, LOH: n = 119, Loss: n = 131), LUAD (WT: n = 162, LOH: n = 190, Loss: n = 87), LUAD_KRAS_hotspot (WT: n = 51, LOH: n = 54, Loss: n = 20), LUSC (WT: n = 57, LOH: n = 222, Loss: n = 123), LUSC_PI3KCA_PEN (WT: n = 20, LOH: n = 102, Loss: n = 58), SKCM (WT: n = 71, LOH: n = 144, Loss: n = 112), SKCM_RAS_hotspot (WT: n = 15, LOH: n = 40, Loss: n = 21), STAD (WT: n = 171, LOH: n = 121, Loss: n = 50), SKCM_BRAF_hotspot (WT: n = 13, LOH: n = 43, Loss: n = 46), PAAD (WT: n = 60, LOH: n = 44, Loss: n = 47), MESO (WT: n = 34, LOH: n = 12, Loss: n = 38), SARC (WT: n = 103, LOH: n = 71, Loss: n = 36), SARC_LMS (WT: n = 39, LOH: n = 22, Loss: n = 8), SARC_MFS/UPS (WT: n = 17, LOH: n = 25, Loss: n = 20), LUAD_EGFRmut (WT: n = 10, LOH: n = 26, Loss: n = 18), LUAD_STK11_truncating (WT: n = 19, LOH: n = 21, Loss: n = 5), LGG_IDHwt (WT: n = 28, LOH: n = 21, Loss: n = 40), STAD_CIN (WT: n = 45, LOH: n = 102, Loss: n = 39), GBM (WT: n = 34, LOH: n = 29, Loss: n = 77), GBM_classic (WT: n = 6, LOH: n = 7, Loss: n = 28), GMB_Mesenchymal (WT: n = 10, LOH: n = 12, Loss: n = 28).

Supplementary Figure 15

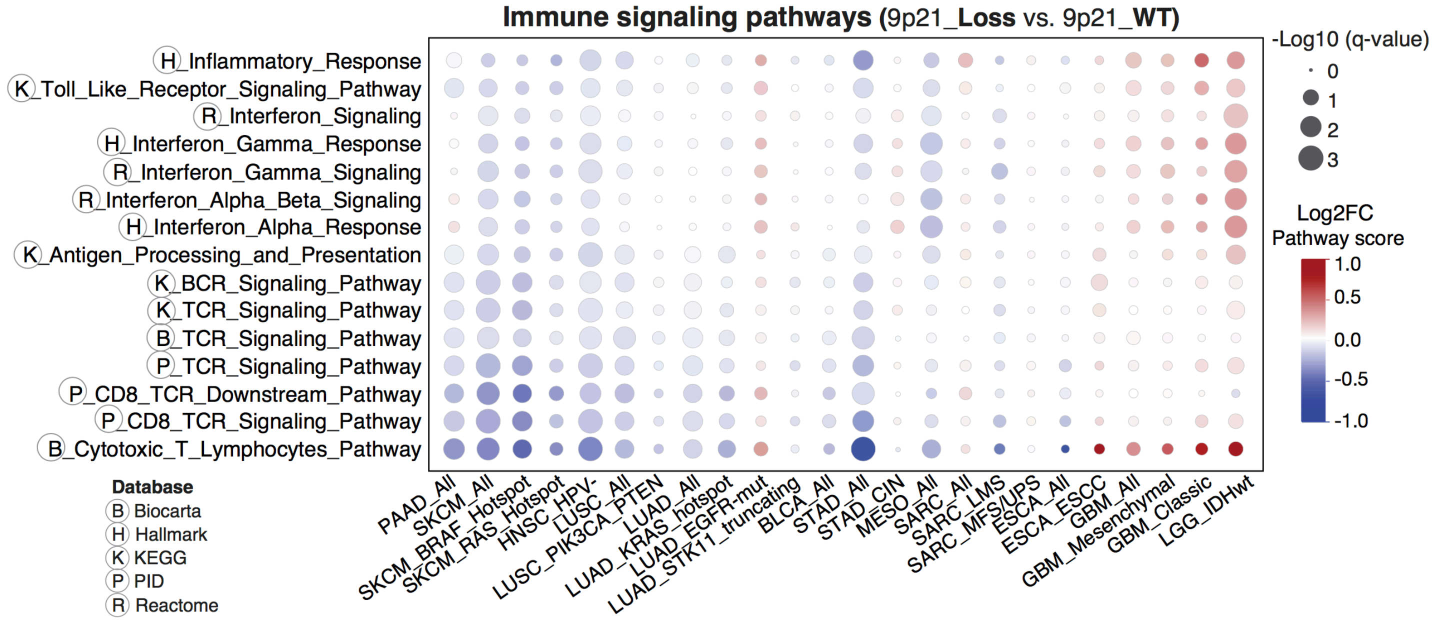

Supplementary Figure 15. The dysregulated immune signaling pathways in tumors with 9p21 loss. A list of 41 immune related pathways (collected from 5 databases, see a full list in Supplementary Data 7) were analyzed and the most significant ones were selected and shown. The size of the bubble indicates statistical significance level of the difference in 9p21-loss tumors (vs. 9p21-WT). The color of the bubble indicates change in the expression level of pathway genes in 9p21-loss tumors (vs. 9p21-WT).

Supplementary Figure 16

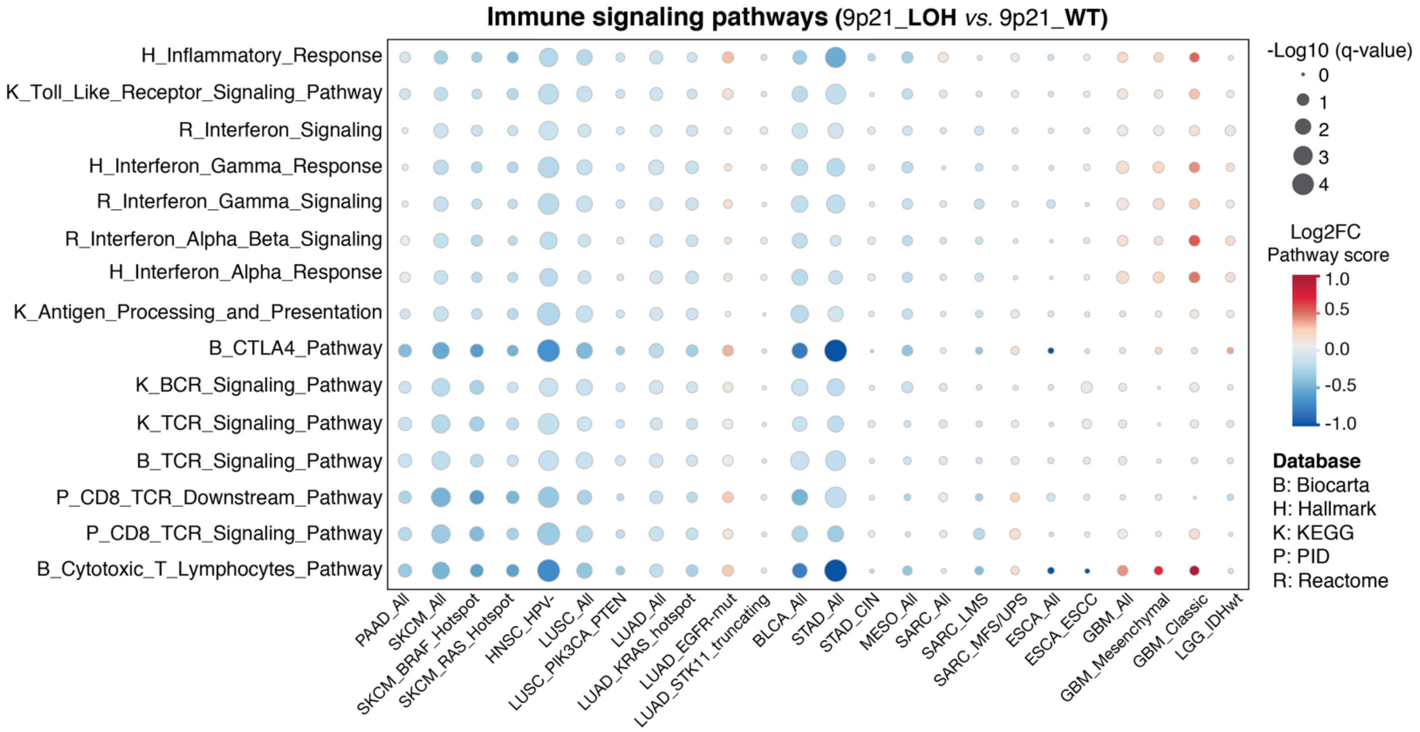

Supplementary Figure 16. Hemizygous deletion of 9p21 (9p21-LOH) is associated with decreased activity of TCR and interferon signaling pathways in TCGA cohorts. For each tumor type and molecular subtype, the pathway scores were compared between tumors with hemizygous deletion of 9p21 (9p21-LOH) and that with intact and wild-type 9p21 (9p21-WT). A list of immune related pathways (see Supplementary Data 7) were analyzed and the most significant ones were selected and shown here. The size of the bubble indicates statistical significance level of the difference in 9p21-loss tumors (vs. 9p21-WT). The color of the bubble indicates change in the expression level of pathway genes in 9p21-loss tumors (vs. 9p21-WT).

Supplementary Figure 17

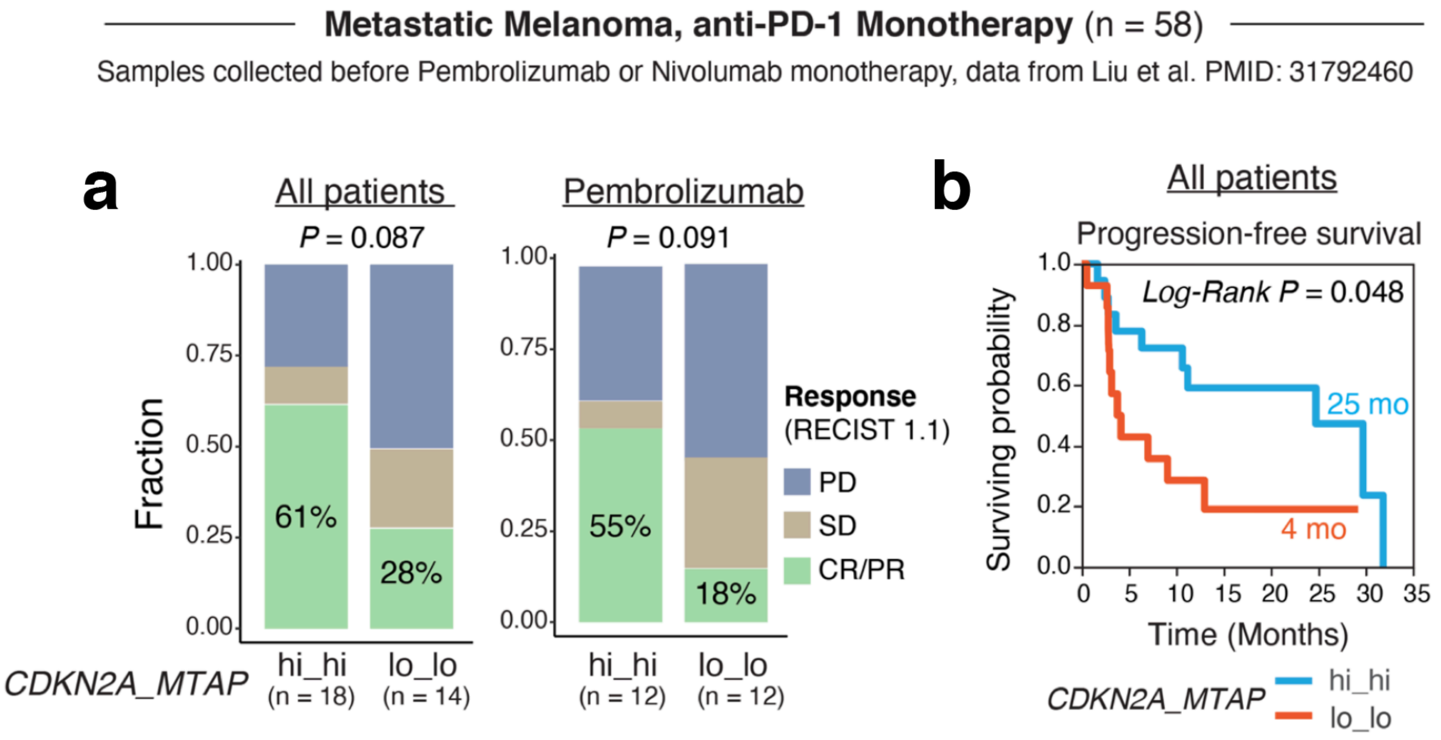

Supplementary Figure 17. The metastatic melanoma cohort from Liu et al. In total, 58 patients received Pembrolizumab or Nivolumab monotherapy were selected for analysis. The best overall responses (RECIST 1.1) were compared between patient groups stratified by 9p21 status. *hi_hi,* tumors with expression levels of both *CDKN2A* and *MTAP* above the group median and *lo_lo*, tumors with expression levels of both genes below the group median. The analysis was performed for all patients together, and for patients who received Pembrolizumab monotherapy. Sample size of each group was labeled on the plot. CR/PR, complete or partial response; SD, stable disease; PD progressive disease. Two-tailed Fisher's exact test was used to calculate the p values in panel **a**. Two-sided Logrank test was used to calculate the p value in panel **b**.

Supplementary Figure 18

Unresectable or advanced Melanoma, Nivolumab Monotherapy (n = 23)

Samples collected before Nivolumab monotherapy, data from Riaz et al. PMID: 29033130

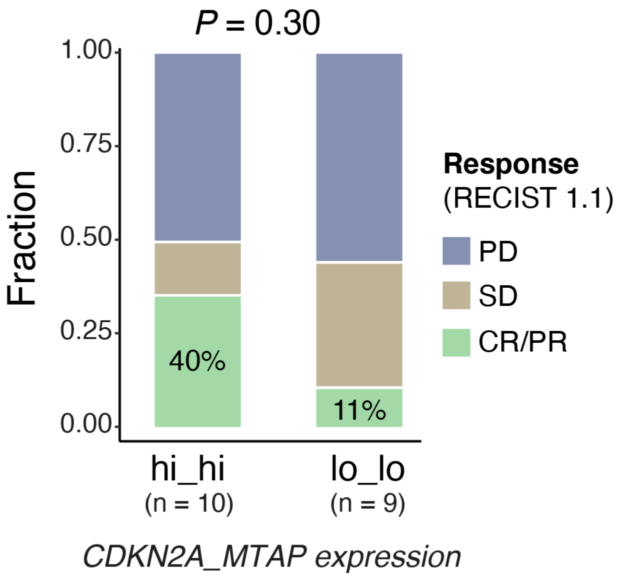

Metastatic Melanoma, anti-PD-1 Monotherapy (n = 41)

Samples collected before Pembrolizumab or Nivolumab monotherapy, data from Gide et al. PMID: 30753825

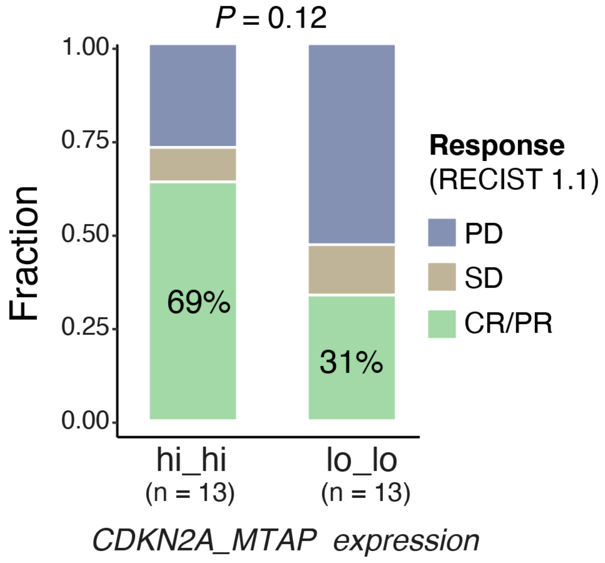

Supplementary Figure 18. The unresectable/advanced melanoma cohorts from Riaz et al. and metastatic melanoma cohort from Gide et al. The best overall responses (RECIST 1.1) were compared between patient groups stratified by 9p21 status. hi_hi, tumors with expression levels of both *CDKN2A* and *MTAP* above the group median and lo_lo, tumors with expression levels of both genes below the group median. Sample size of each group was labeled on the plot. CR/PR, complete or partial response; SD, stable disease; PD progressive disease. Two-tailed Fisher's exact test was used to calculate the p values. Two-tailed Fisher's exact test was used to calculate the p values.

Supplementary Figure 19

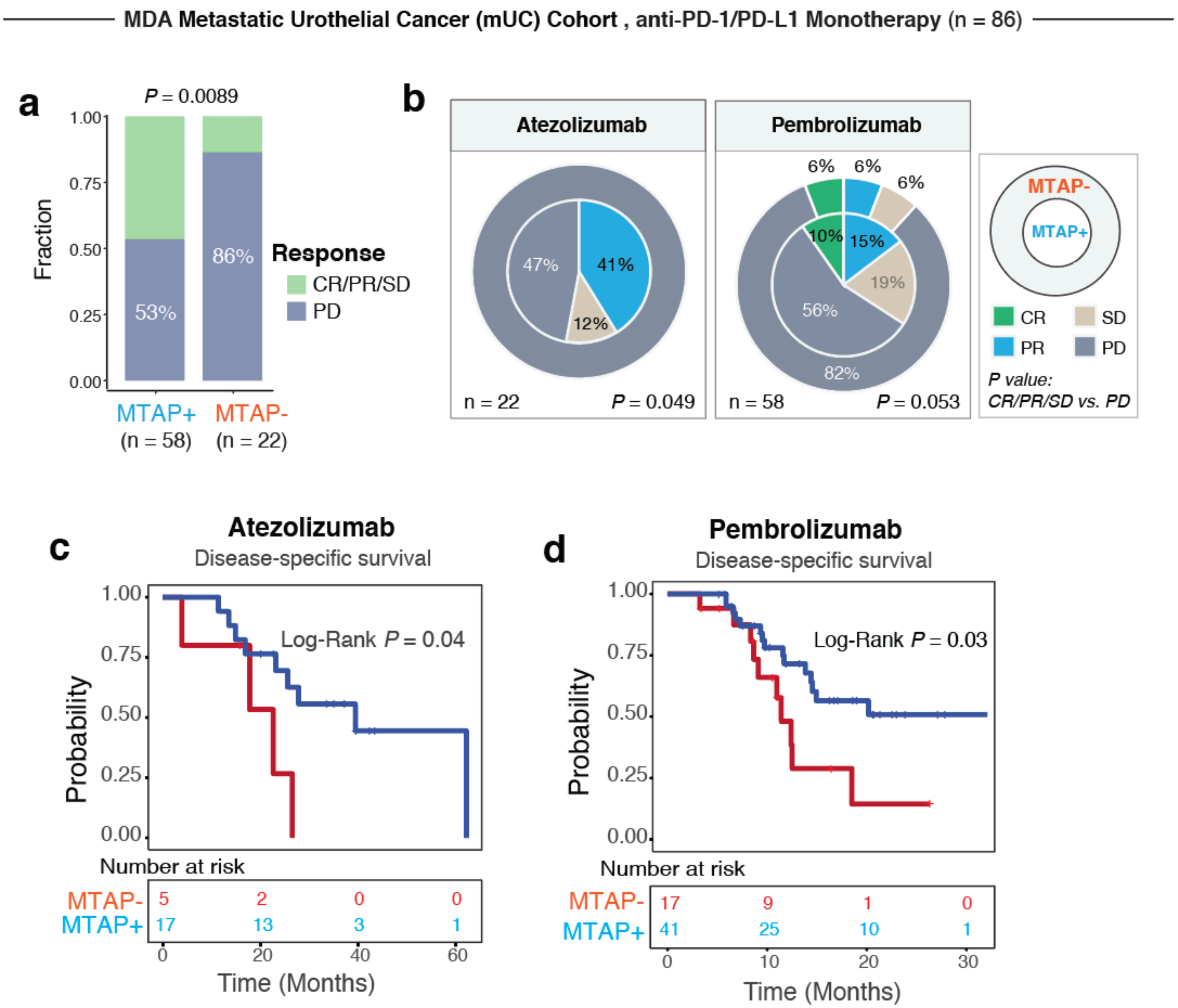

Supplementary Figure 19. MTAP protein loss is associated with poor response to anti-PD-1/L1 therapy in metastatic urothelial cancer (mUC) cohort (related to Fig. 4a-d)**.** A total of 80 patients with MTAP IHC staining and response data available were included. **a**, the fraction of patients experienced disease progression following PD-L1/L1 blockade with atezolizumab or pembrolizumab was increased in patients of the MTAP-negative group. P-value was calculated using the two-tailed Fisher's Exact test. **b,** the best overall response (RECIST 1.1) in individual cohort received atezolizumab or pembrolizumab, respectively. P values were calculated by two-sided Fisher-exact test. **c, d,** Disease-specific survival (DSS) analysis in individual atezolizumab and pembrolizumab cohorts, respectively, showing worse DSS in patients of the MTAP-negative group. P values in panels **c** and **d** were calculated with two-sided Logrank test.

Supplementary Figure 20

MSK NSCLC Cohort (LUAD, n = 151 patients, data from Rizvi et al. 2018. PMID: 29337640)

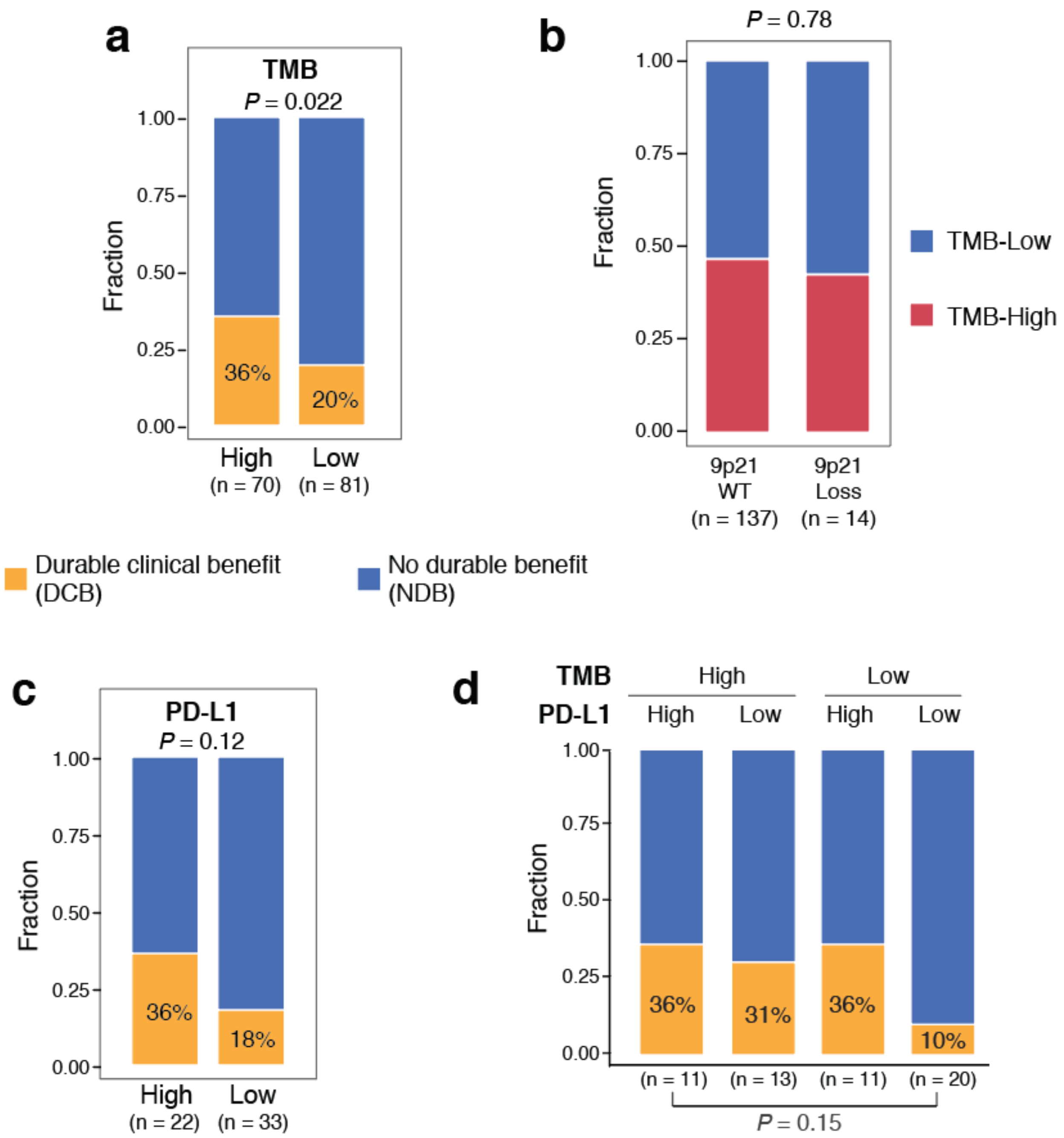

Supplementary Figure 20. The MSK NSCLC cohort from Rizvi et al. A total of 151 lung adenocarcinoma (LUAD) with response and genomic data available were included. 9p21 status was determined based on the genomic data from MSK-IMPACT panel. **a.** The fraction of patients achieved DCB was higher in TMB-high group. **b**. No association was observed between 9p21 status and TMB levels. **c**. The fraction of patients achieved DCB was higher in the group with higher immune cell PD-L1 expression. **d.** A composite of TMB plus immune cell PD-L1 expression for patient stratification. P values were calculated by two-sided Fisher-exact tests.

Supplementary Figure 21

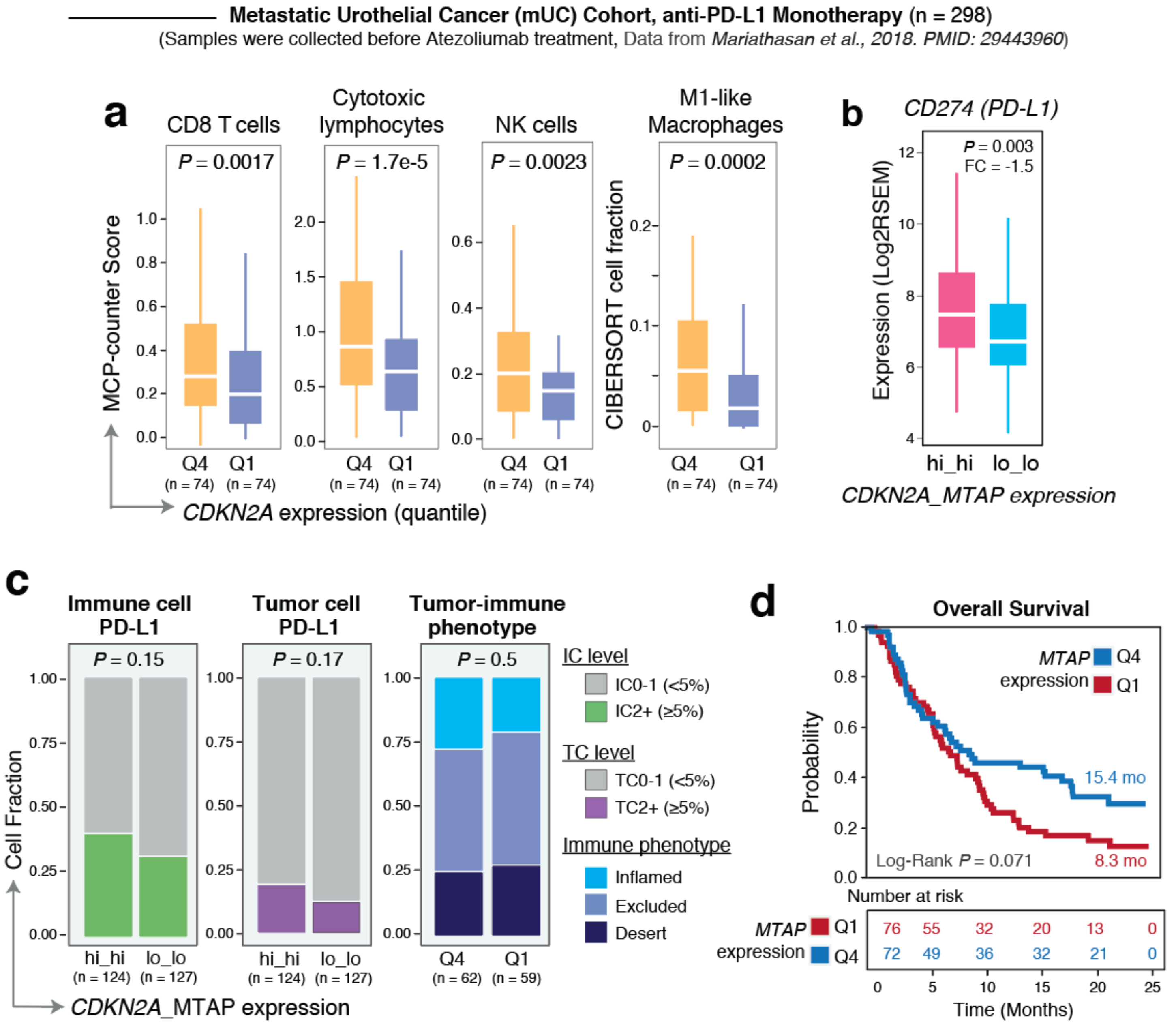

Supplementary Figure 21. The metastatic urothelial cancer (mUC) cohort from Mariathasan et al. A total of 298 mUC patients with response and transcriptome data available for whose pre-treatment tumors were included. **a**. *CDKN2A* expression was stratified into increasing quartiles and the abundance of CD8 T-cells, NK cells, cytotoxic lymphocytes inferred by MCP-counter and the relative proportion of M1-like macrophages estimated by CIBERSORT was significantly lower in tumors with low (Q1) than those with high (Q4) *CDKN2A* expression. P values were calculated by two-sided Wilcoxon rank-sum test. Box, median ± interquartile range; whiskers, 1.5× interquartile range. **b**. Box plot showing downregulated *CD274* (*PD-L1)* mRNA expression in tumors of the *CNKN2A_MTAP:* lo_lo group. hi_hi*,* tumors with expression levels of both *CDKN2A* and *MTAP* above the group median and lo_lo, tumors with expression levels of both genes below the group median. P values were calculated by two-sided Wilcoxon rank-sum test. Box, median ± interquartile range; whiskers, 1.5× interquartile range. hi_hi (n = 124), lo_lo (n = 127). **c**. PD-L1 protein expression levels on both immune and tumor cells measured by IHC staining, and the fraction of inflamed tumors were slightly decreased in the lo_lo group, but no statistical difference was observed. P values were calculated by two-sided Fisher-exact test. **d**. Patients whose pre-treatment tumors had low (Q1) *MTAP* expression showed significantly shortened overall survival than those with high (Q4) *MTAP* expression. P values were calculated with two-sided Logrank test.

Supplementary Figure 22

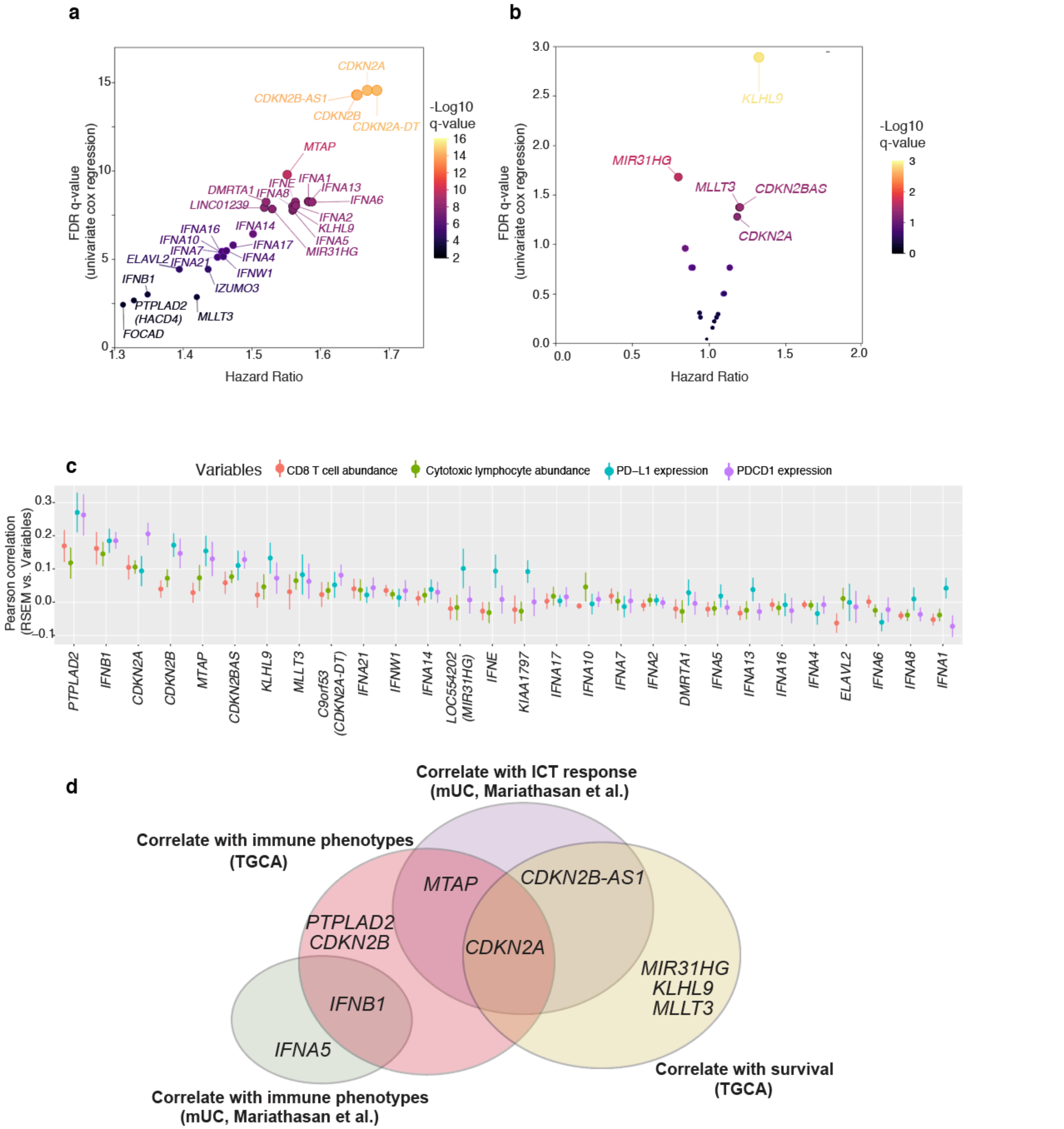

Supplementary Figure 22. Integrative correlation between the genes located at the 9p21.3 region and tumor immune phenotypes. **a**, Univariate Cox regression analysis for the survival significance grouped by the copy number status of genes in 9p21.3. **b**, Univariate Cox regression analysis for the survival significance grouped by the expression level of genes in 9p21.3. Samples were grouped into “high” and “low” based on corresponding gene expression levels (top and bottom quantile). Colors of dots in panels **a** and **b** indicate the significance level. **c**, Correlation of gene expression level and immune phenotypes in TCGA tumors. Error bars indicate the standard deviation and points indicate the mean level of the Pearson correlation coefficient. Sample size: n = 12 for genes including *C9orf53, CDKN2A, CDKN2B, CDKN2BAS, DMRTA1, ELAVL2, IFNA13, IFNA21, IFNB1, IFNE, IFNW1, KIAA1797, KLHL9, LOC554202, MLLT3, MTAP, PTPLAD*. n = 10 for

genes including *IFNA1, IFNA2, IFNA4, IFNA5, IFNA7, IFNA8*. n = 9 for genes including *IFNA10, IFNA14, IFNA17*. *IFNA6* (n = 8). *IFNA16* (n = 5). **d**, Venn diagram displaying significant correlation between genes located in 9p21.3 with tumor immune phenotypes, patient responses to ICT and patient survival.

Supplementary Figure 23

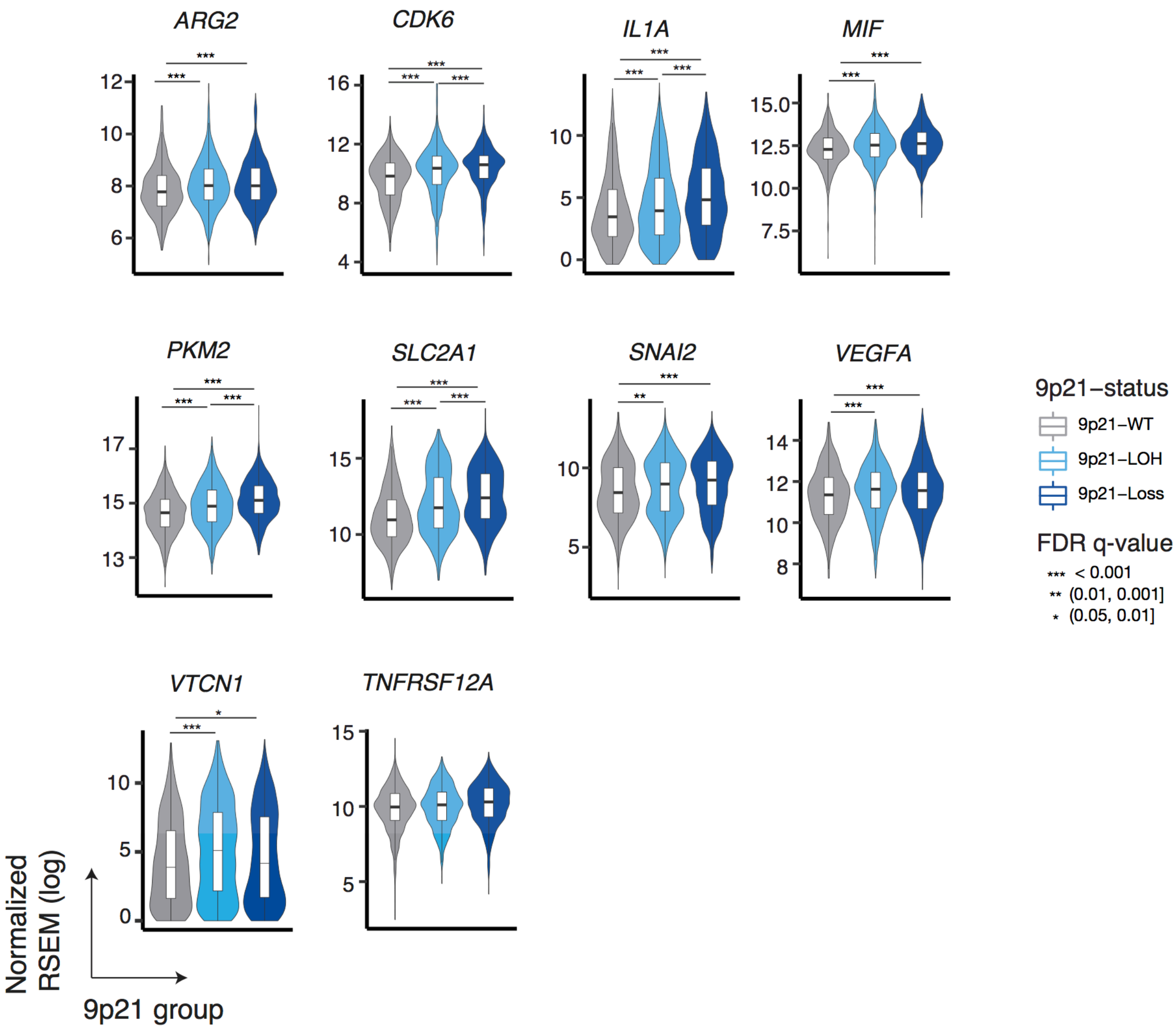

Supplementary Figure 23. Expression level changes of the potential druggable targets identified in 9p21-loss tumors were significantly increased in 9p21-LOH tumors. Significant changes were identified with adjusted q-value < 0.05 and fold change of expression level > 1.2 (9p21-loss vs. 9p21-WT or 9p21-LOH vs. 9p21-WT) as the cutoff. Two-sided Wilcoxon rank-sum tests were used to calculate the p values and were adjusted for multiple testing using the Benjamini–Hochberg method. Box, median ± interquartile range; whiskers, 1.5× interquartile range. Sample size: 9p21-WT (n = 1160), 9p21-LOH (n = 1600), 9p21-Loss (n = 1277).

Supplementary Figure 24

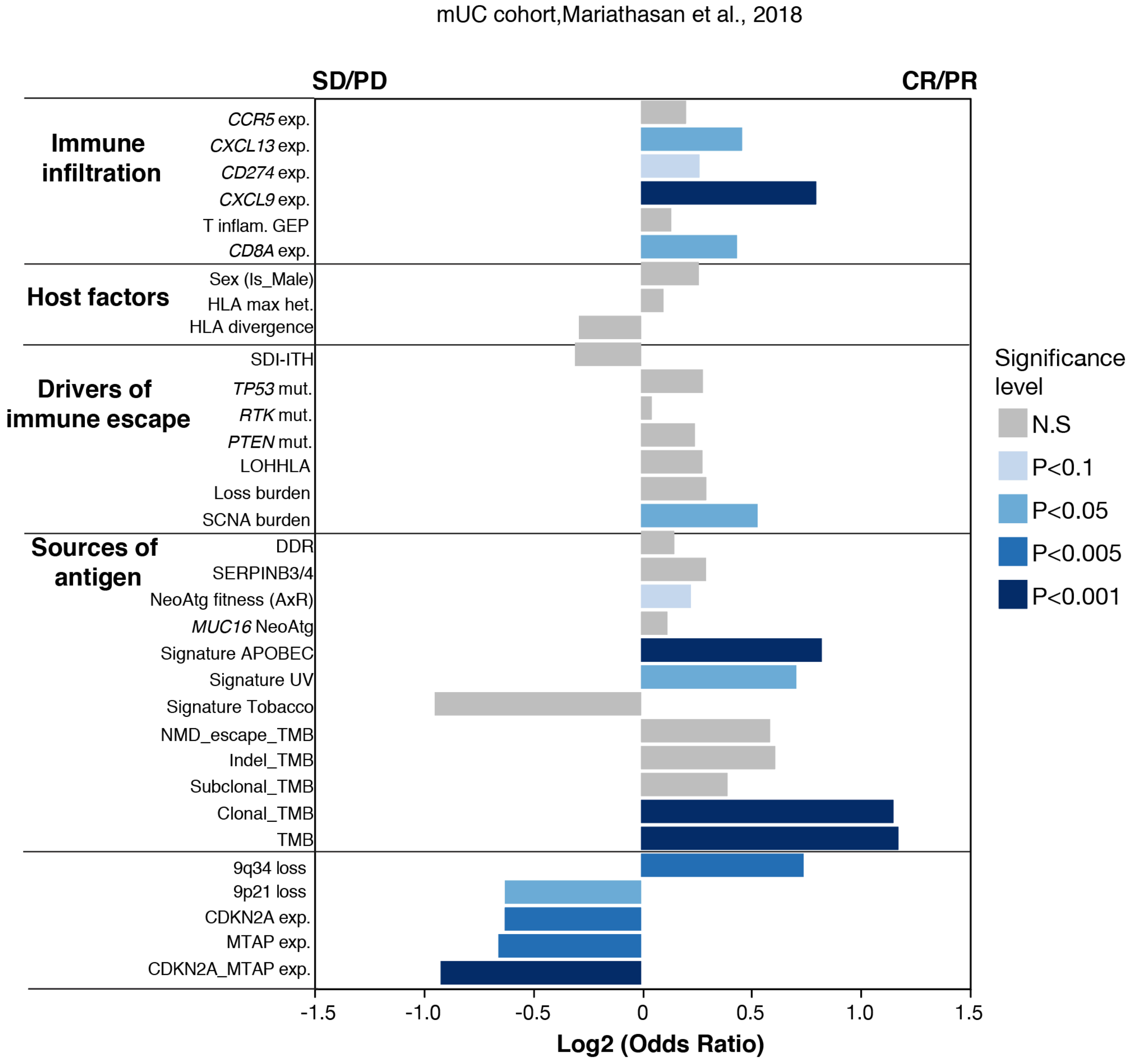

Supplementary Figure 24. Correlation between the potential biomarkers and patient response in a cohort with ICT. The association between the presence or expression levels of each biomarker in four categories (immune infiltration, host factors, drivers of immune escape, and sources of antigen, as defined by Litchfield et al. Cell. 2021) and patient response status were tested. 9q34 loss: copy number loss of 9p34; 9p21 loss: copy number loss of 9p21; CDKN2A_MTAP exp.: The tumors with low (below group median) expression of both *CDKN2A* and *MTAP*; TMB: tumor mutation burden; Indel_TMB: frameshift insertion/deletion (indel) mutation burden; NMD_escape_TMB: burden of indels escaping nonsense mediated decay; MUC16 NeoAtg: MUC16 neoantigens; NeoAtg figness (A×R): neoantigen fitness model; SERPINB3/4: SERPINB3/SERPINB4 mutations; DDR: DNA damage response pathway mutations; SCNA burden: somatic copy-number alteration measured using the weighted genome instability index; Loss burden: copy-number loss burden; LOHHLA: loss of heterozygosity at the HLA locus; SDI-ITH: Shannon diversity index for intratumor heterogeneity; HLA max het.: maximal HLA heterozygosity. T inflam. GEP: T cell inflamed gene expression signatures. exp.: expression. mut.: mutation status. P values were calculated with logistic regression model. Colors of bars indicate the significance level. Sample size: n = 345.
