# Supplementary material for: 9p21 loss confers a cold tumor immune microenvironment and primary resistance to immune checkpoint therapy
Source: Nat Commun. 2021 Sep 23;12:5606. doi: 10.1038/s41467-021-25894-9 (PMC8460828; doi:10.1038/s41467-021-25894-9)
Supplement: Supplementary file 2 — Description of Additional Supplementary Files [file 41467_2021_25894_MOESM2_ESM.pdf]

**Description of Additional Supplementary Files**

Supplementary Data 1
Description: The TCGA samples included in this study and their corresponding 9p21 status. 9p21-WT: tumors with diploid/wildtype 9p21; 9p21-LOH: tumors with LOH (loss of heterozygosity) at both *CDKN2A* and *MTAP* locus. 9p21-Gain: tumors with copy number gain or amplification at 9p21. 9p21-Loss: tumors with homozygous deletion of 9p21.

Supplementary Data 2
Description: The TCGA disease codes and other abbreviations.

Supplementary Data 3
Description: The TCGA cohorts with frequent 9p21 loss (>10%).

Supplementary Data 4
Description: The spatial immune cell patterns and 9p21 loss.

Supplementary Data 5
Description: Summary of TCGA samples with image data.

Supplementary Data 6
Description: The immune cell abundance and 9p21 loss. P values were calculated by two-sided Wilcoxon rank-sum test and adjusted with the Benjamini-Hochberg method.

Supplementary Data 7
Description: The curated gene sets for immune signaling pathway analysis.

Supplementary Data 8
Description: The aberrant immune signaling pathways associated with 9p21 loss. P values were calculated by two-sided Wilcoxon rank-sum test and adjusted with the Benjamini-Hochberg method.

Supplementary Data 9
Description: The immune modulatory genes used in this study.

Supplementary Data 10
Description: Summary of the 8 ICT trials included in this study.

Supplementary Data 11
Description: The MDA solid tumor cohort, anti-PD-1/L1 monotherapy.

Supplementary Data 12
Description: The MDA mUC cohort, anti-PD-1/L1 monotherapy. DSS (days): Disease-specific survival interval in days. PFS (days): Progression-free survival interval in days.

Supplementary Data 13
Description: The MSK NSCLC cohort from Rizvi et al.

Supplementary Data 14

Description: The Metastatic urothelial cancer (mUC) cohort from Mariathasan et al.

Supplementary Data 15
Description: The MCP-counter immune deconvolution results for the mUC cohort from Mariathasan et al..

Supplementary Data 16
Description: The CIBERSORT immune deconvolution results for the mUC cohort from Mariathasan et al..

Supplementary Data 17
Description: List of immune-related genes and therapeutic targets.

Supplementary Data 18
Description: The differentially expressed genes (DEGs) in 9p21-loss tumors in the mUC cohort from Mariathasan et al..

Supplementary Data 19
Description: Genes of interest (Supplementary Data 17) that showed significant correlation with *CDKN2A*/*MTAP* expression in the mUC cohort from Mariathasan et al..

Supplementary Data 20
Description: Genes of interest (Supplementary Data 17) that showed significant correlation with *CDKN2A*/*MTAP* expression in TCGA cohorts.

Supplementary Data 21
Description: Genes located at 9p21.3 loci.
