# Supplementary material for: 9p21 loss confers a cold tumor immune microenvironment and primary resistance to immune checkpoint therapy
Source: Nat Commun. 2021 Sep 23;12:5606. doi: 10.1038/s41467-021-25894-9 (PMC8460828; doi:10.1038/s41467-021-25894-9)
Supplement: Supplementary file 4 — Reporting Summary [file 41467_2021_25894_MOESM4_ESM.pdf]

nature research

Corresponding author(s): Linghua Wang

Last updated by author(s): Aug 19, 2021

Reporting Summary

Nature Research wishes to improve the reproducibility of the work that we publish. This form provides structure for consistency and transparency in reporting. For further information on Nature Research policies, see our Editorial Policies and the Editorial Policy Checklist.

Statistics

For all statistical analyses, confirm that the following items are present in the figure legend, table legend, main text, or Methods section.

n/a	Confirmed	
☐	☒	The exact sample size (*n*) for each experimental group/condition, given as a discrete number and unit of measurement
☐	☒	A statement on whether measurements were taken from distinct samples or whether the same sample was measured repeatedly
☐	☒	The statistical test(s) used AND whether they are one- or two-sided<br>*Only common tests should be described solely by name; describe more complex techniques in the Methods section.*
☐	☒	A description of all covariates tested
☐	☒	A description of any assumptions or corrections, such as tests of normality and adjustment for multiple comparisons
☐	☒	A full description of the statistical parameters including central tendency (e.g. means) or other basic estimates (e.g. regression coefficient) AND variation (e.g. standard deviation) or associated estimates of uncertainty (e.g. confidence intervals)
☐	☒	For null hypothesis testing, the test statistic (e.g. *F*, *t*, *r*) with confidence intervals, effect sizes, degrees of freedom and *P* value noted<br>*Give P values as exact values whenever suitable.*
☒	☐	For Bayesian analysis, information on the choice of priors and Markov chain Monte Carlo settings
☐	☒	For hierarchical and complex designs, identification of the appropriate level for tests and full reporting of outcomes
☐	☒	Estimates of effect sizes (e.g. Cohen's *d*, Pearson's *r*), indicating how they were calculated

Our web collection on statistics for biologists contains articles on many of the points above.

Software and code

Policy information about availability of computer code

Data collection	No software was used for data collection. All softwares and code used in this study are publicly available as described in METHODS. A detailed description of patient cohorts, clinical characteristics, data collection and filtering was included in the Methods.
Data analysis	R version 3.4.3 was used for the statistical analysis. JMP Pro (v14) was used for visualization and illustration. STAR (v2.7.2b) was used for RNA-seq data sequence alignment. Gene level expression quantification was performed using HTSeq-count (v0.11.0). GSVA r package (v1.32.0) was used for the sample level gene set analysis. MCP-counter (v1.1.0) r package was used to infer the absolute abundance score of immune cell types. Cibersort (https://cibersort.stanford.edu/) was used to infer the relative abundance scores of immune cell types. R package survival (v3.1-11) was used for survival analysis. Further details are provided in the Methods.

For manuscripts utilizing custom algorithms or software that are central to the research but not yet described in published literature, software must be made available to editors and reviewers. We strongly encourage code deposition in a community repository (e.g. GitHub). See the Nature Research guidelines for submitting code & software for further information.

Data

Policy information about availability of data

All manuscripts must include a data availability statement. This statement should provide the following information, where applicable:

- Accession codes, unique identifiers, or web links for publicly available datasets
- A list of figures that have associated raw data
- A description of any restrictions on data availability

For TCGA cohorts, the genomic and clinical data can be retrieved from NCI Genomic Data Commons (NCI-GDC: https://gdc.cancer.gov). For the melanoma cohorts from Helmink et al. (GSE120575[https://www.ncbi.nlm.nih.gov/geo/query/acc.cgi?acc=GSE120575]) and Riaz et al. (GSE91061[https://www.ncbi.nlm.nih.gov/geo/query/acc.cgi?acc=GSE91061]), the data can be obtained from the Gene Expression Omnibus (GEO) database[https://www.ncbi.nlm.nih.gov/geo/]. Data of the

April 2020

Urothelial cancer cohort from Mariathasan et al. (mUC IMvigor210 trial) can be downloaded from http://research-pub.gene.com/IMvigor210CoreBiologies. Data of the MSKCC advanced NSCLC cohort from Rizvi et al. can be obtained from the cBioPortal[https://www.cbioportal.org/]. The clinical response data of MDA solid tumor cohort and MDA metastatic urothelial cancer cohort were shared in Supplementary Data 11 and Supplementary Data 12, respectively. The data that support the main findings of this study are provided in Supplementary Data 3, 4, 6, 8,11-16, 18-20. For MDA mUC cohort and MDA solid tumor cohort, the patient related data (sex, age, diagnosis, date of last follow up) not included in the paper are subjected to patient confidentiality. Further information and requests should be directed to and will be fulfilled by the Lead Contact, Dr. Linghua Wang (LWang22@mdanderson.org). All requests for data and materials will be promptly reviewed by The University of Texas MD Anderson Cancer Center to verify if the request is subject to any intellectual property or confidentiality obligations. Any data and materials that can be shared will be released via a Material Transfer Agreement.

Field-specific reporting

Please select the one below that is the best fit for your research. If you are not sure, read the appropriate sections before making your selection.

☒ Life sciences ☐ Behavioural & social sciences ☐ Ecological, evolutionary & environmental sciences

For a reference copy of the document with all sections, see nature.com/documents/nr-reporting-summary-flat.pdf

Life sciences study design

All studies must disclose on these points even when the disclosure is negative.

Sample size	The TCGA cohorts included 104,35 patients across 33 tumor types. Among them, 12 cancer types with frequent (>10%, n = 3,355) 9p21 loss were selected for downstream analyses. For ICT cohorts from public datasets (n = 889), further filtering was applied (e.g., therapy received, diagnosis, gene expression, genomic data availability, etc.) and 583 samples were retained for subsequent analyses. The sample size for each figure panel was clearly labeled either in the figure or in its figure legend.
Data exclusions	For TCGA cohorts, we focused on cancers with frequent 9p21 loss and cancer types with less frequent 9p21 loss (<10%) were filtered out and excluded from downstream analysis. For ICT cohorts, to reduce the potential confounding effects, patients received combined ICT therapy or had prior ICT therapy were excluded. For the MDA solid tumor cohort, the following criteria were applied for data exclusions: 1) Patients treated with other therapy instead of anti-PD-1/PD-L1 monotherapy. 2) Lack of clinical response data. 3) Data records without matched controls. For the MDA mUC cohort, patients without PET/CT images for response evaluation were excluded. For the MSK Advanced NSCLC cohort, data exclusion criteria included: 1) Patients were not diagnosed with lung adenocarcinoma. 2) Patients did not receive anti-PD-1/PD-L1 monotherapy. 3) Corresponding response data were not available. 4) Samples were collected post treatment or samples had truncating CDKN2A/MTAP mutations. For the mUC cohort from the IMvigor210 Trial, all 298 samples were included. Data exclusions for the melanoma cohorts: 1) Patients treated with other therapy instead of anti-PD-1 monotherapy. 2) Specimens collected after treatment. 3) Patients with mixed response. 4) Lack of RNA-seq data. 5) Lack of response data.
Replication	The findings of 9p21 as potential biomarker for poor outcome was observed in TCGA cohort and other 4 published melanoma cohorts and then validated in 1) two metastatic urothelial cancer (mUC) cohorts (n = 80 and n = 298, respectively) and 2) LUAD cohort (n = 151).
Randomization	This is not a study involving human subjects in clinical trials. This study utilized de-identified genomic and clinical data derived from retrospective ICT trials, and thus randomization does not apply to this study.
Blinding	This is not a study involving human subjects in clinical trials, blinding does not apply to this study.

Reporting for specific materials, systems and methods

We require information from authors about some types of materials, experimental systems and methods used in many studies. Here, indicate whether each material, system or method listed is relevant to your study. If you are not sure if a list item applies to your research, read the appropriate section before selecting a response.

Materials & experimental systems

n/a	Involved in the study	
☒	☐	Antibodies
☒	☐	Eukaryotic cell lines
☒	☐	Palaeontology and archaeology
☒	☐	Animals and other organisms
☒	☐	Human research participants
☒	☐	Clinical data
☒	☐	Dual use research of concern

Methods

n/a	Involved in the study	
☒	☐	ChIP-seq
☒	☐	Flow cytometry
☒	☐	MRI-based neuroimaging
